# Dynamic pH responsivity of triazole-based self-immolative linkers†

**DOI:** 10.1039/d0sc00532k

**Published:** 2020-03-03

**Authors:** Derrick A. Roberts, Ben S. Pilgrim, Tristan N. Dell, Molly M. Stevens

**Affiliations:** Key Center for Polymers and Colloids, School of Chemistry, The University of Sydney Sydney NSW 2006 Australia derrick.roberts@sydney.edu.au; Department of Medical Biochemistry and Biophysics, Karolinska Institutet 171 77 Stockholm Sweden; School of Chemistry, The University of Nottingham Nottingham NG7 2RD UK; Department of Materials, Department of Bioengineering, Institute for Biomedical Engineering, Imperial College London London SW7 2AZ UK m.stevens@imperial.ac.uk

## Abstract

Gating the release of chemical payloads in response to transient signals is an important feature of ‘smart’ delivery systems. Herein, we report a triazole-based self-immolative linker that can be reversibly paused or slowed and restarted throughout its elimination cascade in response to pH changes in both organic and organic-aqueous solvents. The linker is conveniently prepared using the alkyne–azide cycloaddition reaction, which introduces a 1,4-triazole ring that expresses a pH-sensitive intermediate during its elimination sequence. Using a series of model compounds, we demonstrate that this intermediate can be switched between active and dormant states depending on the presence of acid or base, cleanly gating the release of payload in response to a fluctuating external stimulus.

## Abstract 2

Triazole-based self-immolative linkers can be reversibly paused and restarted throughout their elimination cascades in response to environmental pH changes.

## Graphical abstract

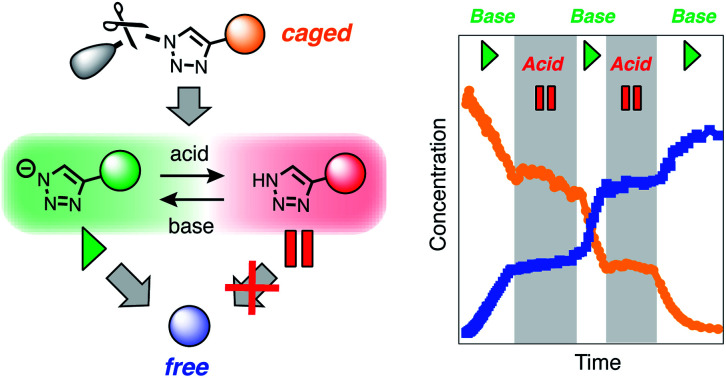

## Introduction

Chemists have long pursued stimuli-specific strategies for activating latent molecules, motivated by applications in controlled release,^1^ sensing,^2^ imaging,^3^ and signal amplification.^4^ To this end, ‘self-immolation’ has emerged as a powerful tool. Originally conceived in the 1980s to improve prodrug activation,^5^ self-immolation is the spontaneous and irreversible fragmentation of a multicomponent compound into small molecules through a cascade of cyclisation or elimination reactions—a process often likened to the falling of dominoes or the burning of a fuse.^6^ Molecules that undergo self-immolation have been adapted as linkers for temporarily connecting a cleavable protecting group (designated as the ‘trigger’) to a chemical payload.^7^ These linkers maintain space between the trigger and payload and enhance the entropic driving force that promotes complete and traceless deprotection.^8^ These unique qualities have inspired numerous studies into the development of self-immolative linkers for the controlled release of small molecules^9^ and, more recently, for constructing self-immolative polymers that undergo complete head-to-tail depolymerisation upon trigger cleavage.^10^

Self-immolative linkers are ideally activated by removal of the trigger group in response to a specific chemical or biological stimulus.^11^ After trigger cleavage, the ensuing self-immolation sequence proceeds along an uninterrupted kinetics trajectory that is largely predetermined by steric and electronic properties of the linker.^12^ By contrast, remarkably few studies have investigated ways to dynamically control the kinetics of self-immolation using external signals after removal of the trigger. Recently, de Alaniz and co-workers reported the first example of reversible ‘pausing’ of a self-immolative depolymerisation reaction in response to temperature changes.^13^ This work highlights an important advantage of dynamically responsive self-immolation: the ability to switch the cascade between active and paused states ensures that payload delivery proceeds only under specific environmental conditions and slows or ceases entirely if those conditions suddenly change. Considering the paucity of reported examples, there are unexplored opportunities for dynamically controlling self-immolation kinetics using transient or fluctuating signals.

It is well known that self-immolative linkers containing basic residues are sensitive to changes in acidity.^12^ This feature is exploited during the synthesis of cyclisation–elimination self-immolative polymers^14^ and, most recently, for tuning the initial release kinetics of a self-immolative H_2_S delivery agent.^15^ However, to the best of our knowledge there are no studies that use transient changes in acidity to dynamically switch a self-immolative linker between active and dormant states following trigger removal. Herein we investigate triazole-based self-immolative linkers that can be reversibly paused or slowed and restarted throughout their self-immolation sequences upon addition of acid and base to the reaction medium. The pausing mechanism relies on the formation of a metastable intermediate during linker degradation that eliminates under mild basic conditions but remains stable in more acidic conditions. By alternating between acidic and basic conditions, the cascade can be switched rapidly between active and paused states to control the payload release rate even after the trigger group is removed.

## Results and discussion

**Scheme 1:**
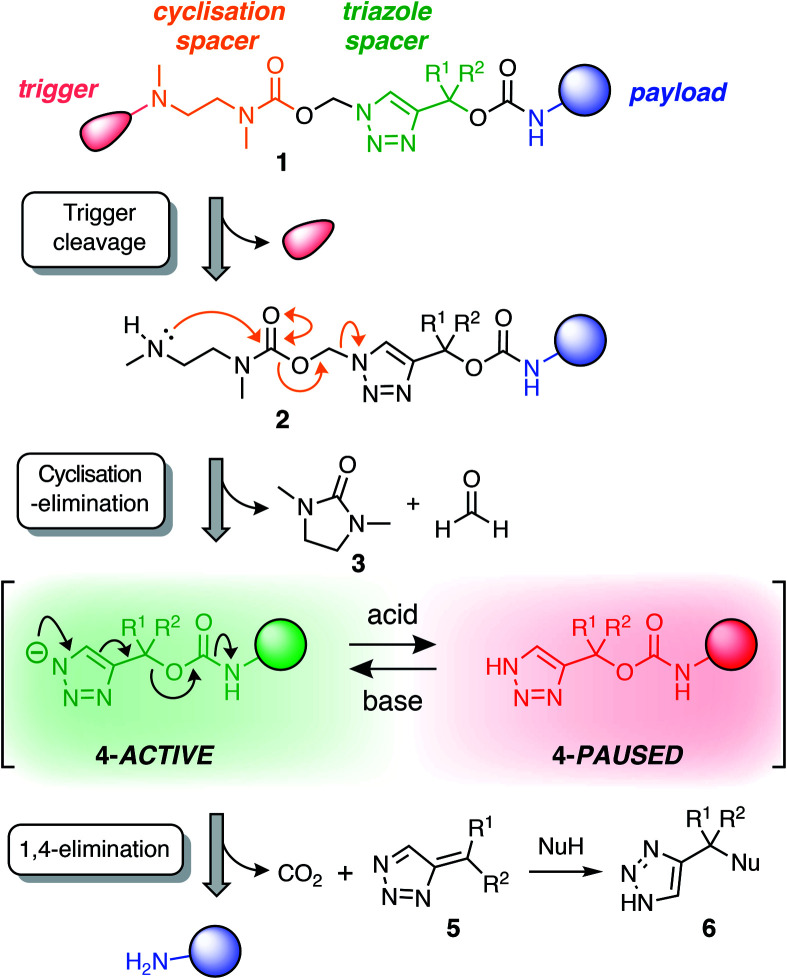
Overview of the self-immolation sequence for hybrid diamine–triazole linkers reported herein. Trigger removal from **1** leads to the formation of 1*H*-triazole **4**, which can be reversibly switched between active and paused states to gate the release of the payload molecule. ‘Nu’ denotes a generic nucleophile. R-groups are specified in Scheme 2.

Our linker system is comprised of a diamine-derived cyclisation spacer^14^ connected in series to a triazole 1,4-elimination spacer (Scheme 1).^16^ Incorporating a diamine-derived spacer next to the triazole ring permits the use of carbamate trigger groups,^17^ which drastically expands the scope of ester triggers reported previously.^18^ Removal of the trigger group from **1** under basic conditions exposes a secondary amine nucleophile (**2**) that undergoes a cyclisation–elimination reaction to afford a cyclic urea (**3**) and a short-lived triazolyl-1-methanol species. Rapid elimination of formaldehyde affords a metastable 1*H*-triazole intermediate (**4**). Under basic conditions, triazole **4** undergoes 1,4-elimination *via* a triazolide anion to release the payload molecule, carbon dioxide and triazafulvene **5**, which is rapidly converted to **6***via* a Michael addition. However, addition of acid to the medium hinders deprotonation of 1*H*-triazole **4**, thereby pausing or slowing (depending on the position of equilibrium) self-immolation. Addition of a suitable base reactivates the cascade, which proceeds to completion along the same kinetics profile as before acidification.

**Scheme 2:**
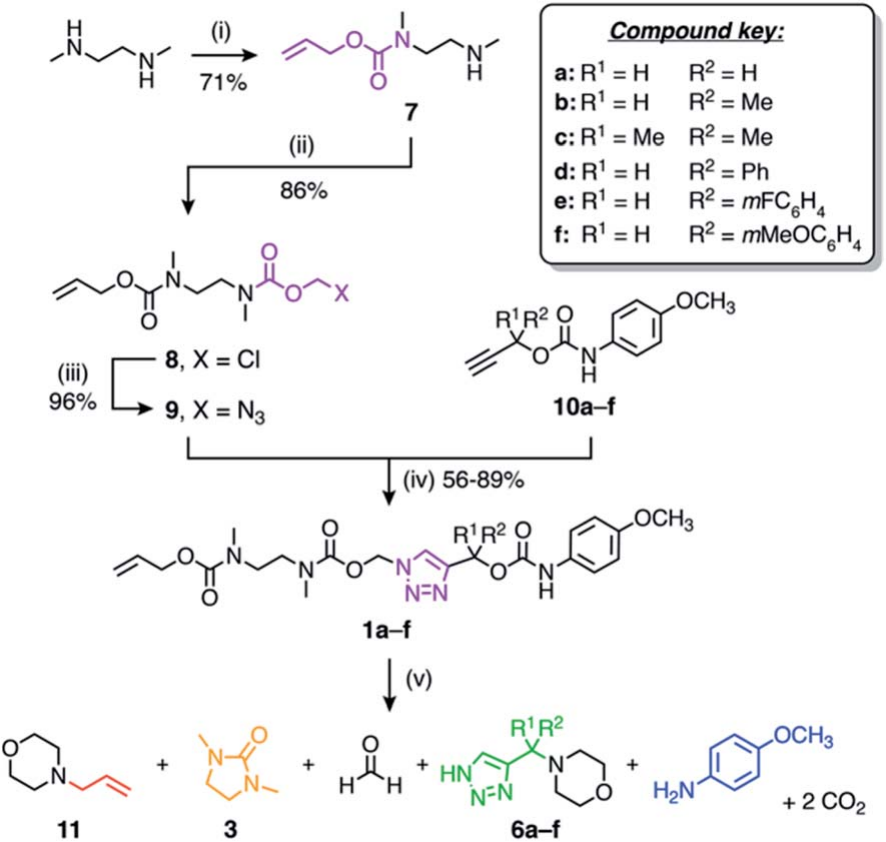
Synthesis and self-immolation of model compounds **1a–f**. *Conditions*: (i) allyl phenyl carbonate, EtOH, rt; (ii) chloromethyl chloroformate, pyridine, CHCl_3_, 0 °C to rt; (iii) NaN_3_, DMF, 50 °C; (iv) CuSO_4_, sodium ascorbate, DMF, 50 °C; (v) Pd(PPh_3_)_4_, morpholine (50 equiv.), DMSO-*d*_6_, 60 °C.

The self-immolative linker is formed by a copper(i)-catalysed azide–alkyne cycloaddition (CuAAC) reaction between azide **9** and a propargylic carbamate-protected payload molecule (**10a–f**) (Scheme 2). To standardise the initial trigger activation step, the model systems studied herein feature an allyl carbamate trigger group that is cleaved rapidly in the presence of palladium(0) under basic conditions. Allyl-protected azide **9** was synthesised in ∼60% yield over three steps starting with the treatment of 1,2-dimethylethylenediamine with allyl phenyl carbonate to furnish mono-protected amine **7** in good yield. Subsequent treatment with chloromethyl chloroformate gave **8**, followed by azidation with sodium azide in DMF to afford azide **9**.

Russell and co-workers established that the rate of 1,4-elimination across a triazole ring is sensitive to substituents at the triazole α-methine position, enabling tuning of the payload release rate.^18^ To ascertain if this sensitivity is preserved in our system, we synthesised a series of α-substituted propargylic carbamates (**10a–f**) carrying a *p*-anisidine model payload. Model compounds **1a–f** were subsequently prepared by treating alkynes **10a–f** with azide **9** under copper(i) catalysis in DMF at 50 °C (Scheme 2). NMR and MS analyses confirmed successful formation of **1a–f**, and FTIR confirmed loss of the alkyne and azide stretching frequencies following CuAAC coupling (ESI, Section S5†).

Detailed analyses of the self-immolation kinetics of model compounds **1a–1f** were performed in DMSO-*d*_6_ to elucidate the self-immolation mechanism in a non-nucleophilic solvent. Reactions were monitored by *in situ*^1^H NMR spectroscopy at 60 °C (ESI, Section S6†).^19^ Linkers were activated by adding a suspension of Pd(PPh_3_)_4_ in DMSO-*d*_6_ to a solution of the model compound (10–30 mM) containing excess morpholine (50 equiv.) to render all base-mediated reaction steps pseudo-first-order. Control experiments confirmed that the model compounds were stable towards base in the absence of Pd(PPh_3_)_4_ (ESI, Fig. S70†).

**Scheme 3:**
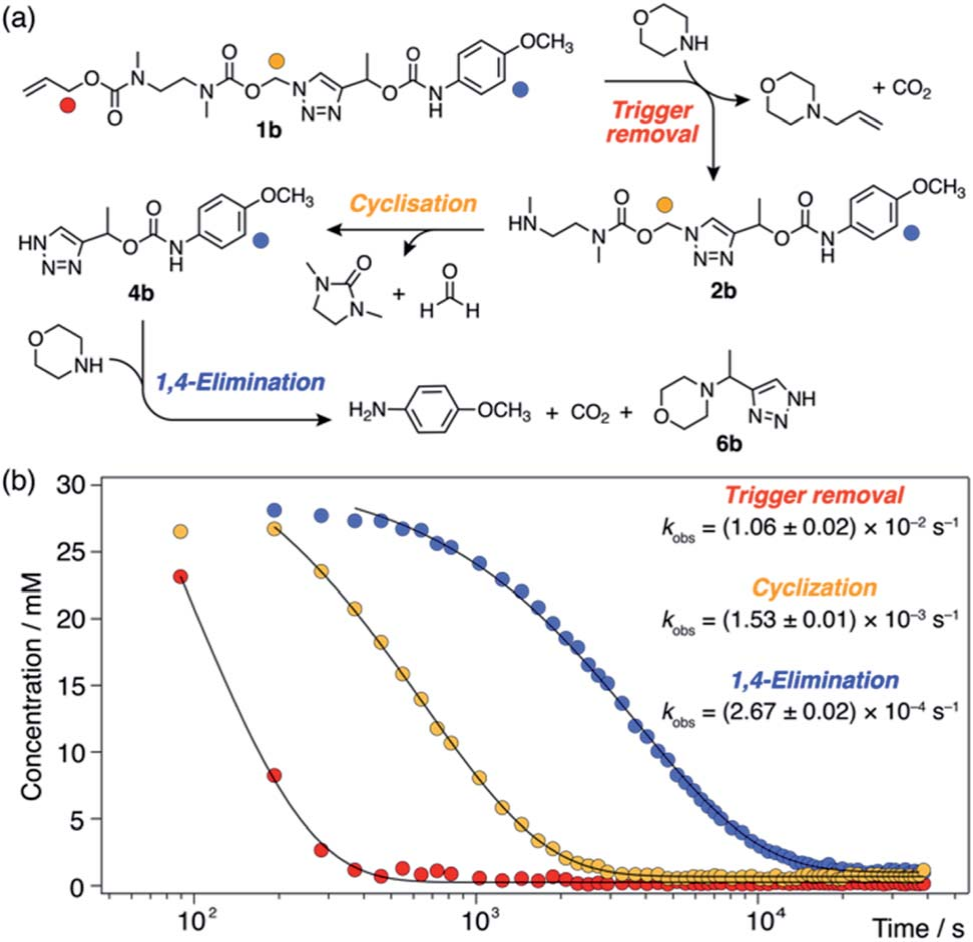
Base-mediated self-immolation kinetics of model **1b** (DMSO-*d*_6_, 60 °C), shown as a representative example. (a) Reaction scheme illustrating the three distinguishable steps in the self-immolation reaction. Markers denote proton environments tracked in (b). (b) Kinetics profiles showing different stages of the cascade. Pseudo-first-order rate constants (*k*_obs_) were calculated by fitting experimental data to monoexponential decays.

**Fig. 1:**
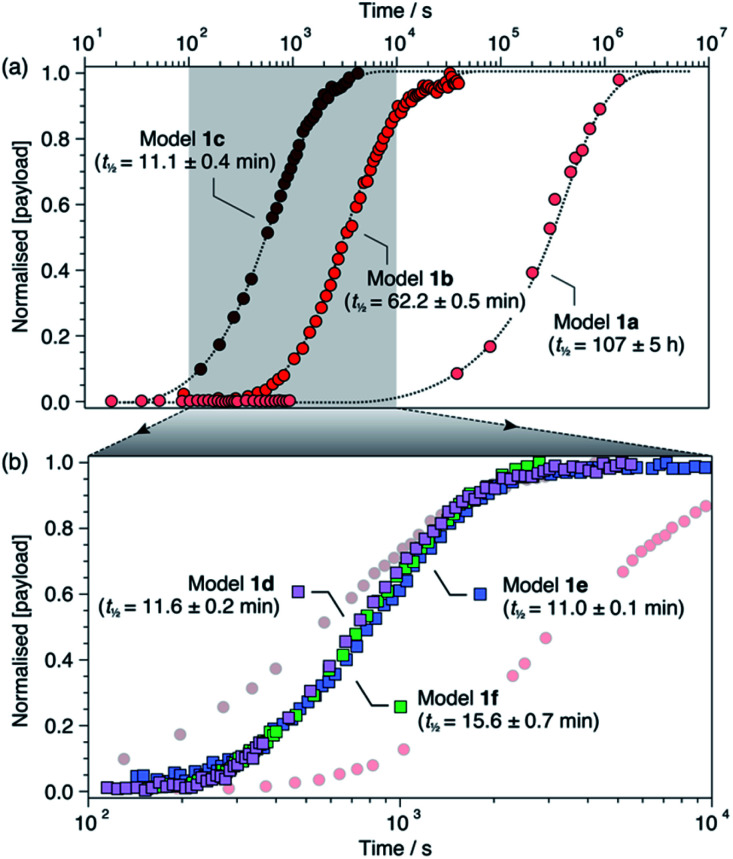
Normalised payload release profiles for (a) **1a–c** and (b) **1d–f** (**1b** and **1c** shown in background) in DMSO-*d*_6_ at 60 °C with 50 equiv. morpholine. Release half-lives are shown in parentheses and were calculated from fitting analyses (ESI, Section S6†). Dotted lines are included as visual guides.

For each model compound, the three stages of the self-immolation cascade were distinguishable by NMR, allowing calculation of pseudo-first-order rate constants (*k*_obs_) for each step (Scheme 3; ESI, Section S6.6†). Rate constants for trigger removal and cyclisation–elimination were consistent between compounds, proceeding on the order of 10^−2^ s^−1^ and 10^−3^ s^−1^, respectively. For both stages, elimination products (urea **3** and amine **11**, Scheme 2) were identified by comparison with reference ^1^H NMR spectra (Section S6.4†). By contrast, collapse of 1*H*-triazole **4***via* 1,4-elimination varied widely between the different compounds, ranging from minutes (**1c–f**) and hours (**1b**) up to several days (**1a**) to achieve maximum (*ca.* 90%) ^20^ payload release (Fig. 1). As anticipated, the rate of 1,4-elimination was influenced by the triazole α-methine substituent to a degree, whereby rates increased with the degree of substitution, and thus stability, of triazafulvene **5**. However, we did not observe appreciable differences between 1,4-elimination rates of **1c–f** due to competition between the cyclisation and 1,4-elimination steps for these compounds (Fig. 4b; ESI, Section S6.6 for details†).^21^ Nonetheless, rate differences between **1a**, **1b** and **1c–f** illustrate payload delivery across a wide range of release lifetimes.

For all models except **1c**^22^ we observed transient formation of 1*H*-triazole intermediate **4** (identified by a ^1^H signal around 7.7 ppm), which formed at the same rate as the cyclisation products and was consumed at the same rate as payload release (ESI, Fig. S76†). The triazolide anion, which has been detected in strongly basic media,^18^ was not directly observed under the mildly basic conditions provided by morpholine. This is consistent with the p*K*_a_ difference between morpholinium (∼9.2 in DMSO^23^) and 1*H*-1,2,3,-triazole (∼13.9 in DMSO^24^), which suggests that intermediate **4** exists predominantly in its 1*H*-triazole form during the cascade with only a small fraction dissociated at any time. Despite limited dissociation, **4** undergoes efficient 1,4-elimination due to irreversible loss of CO_2_, leading to efficient release of the anisidine payload.

Having established that models **1a–f** undergo successful self-immolation in DMSO-*d*_6_, we sought to evaluate their behaviour in the presence of water. *In situ*^1^H NMR kinetics using models **1a**, **1b** and **1d** (chosen to cover the slow, medium and fast kinetics regimes observed in DMSO-*d*_6_) were performed in DMSO-*d*_6_/D_2_O (8 : 2, v/v) at 60 °C. Poor solubility of the compounds in neat D_2_O precluded higher aqueous fractions. Control experiments showed that no background hydrolysis occurred in DMSO-*d*_6_/D_2_O (8 : 2, v/v) containing morpholine (50 equiv.), confirming the excellent stability of all three compounds (ESI, Section 7.1†). Pd(PPh_3_)_4_ successfully initiated the cascades following brief induction periods of 1–3 min reflecting delayed trigger removal (ESI, Section 7.2†), most likely due to reduced solubility of Pd(PPh_3_)_4_ in the presence of water. Following the induction periods, self-immolation proceeded smoothly for all compounds. Interestingly, pseudo-first-order rate constants for trigger removal and cyclisation–elimination were similar to those recorded in DMSO-*d*_6_. However, 1,4-elimination was four-fold (**1d**), 11-fold (**1b**) and 30-fold (**1a**) faster in the presence of D_2_O, which we attribute to rapid nucleophilic attack of triazafulvene **5** by the solvent (ESI, Table S3†). A similar trend in payload release rates was observed, with half-lives of 3.45 ± 0.06 h (**1a**), 5.85 ± 0.14 min (**1b**) and 2.94 ± 0.07 min (**1d**).

**Scheme 4:**
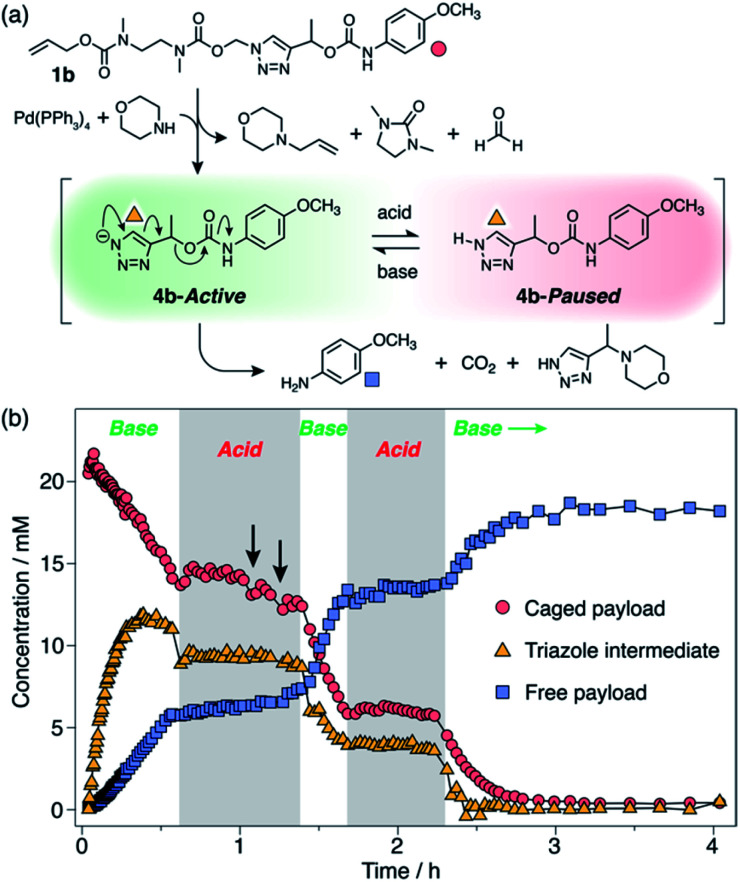
Representative acid-mediated pausing and reactivation of model **1b** (DMSO-*d*_6_, 60 °C). (a) Scheme illustrating the switching equilibrium. Markers denote proton environments tracked in (b). The base is morpholine or Cs_2_CO_3_. (b) Kinetics profiles of key species during self-immolation. Arrows denote attempts to restart the cascade using sub-stoichiometric Cs_2_CO_3_.

Observation of 1*H*-triazole **4** as a key intermediate in the self-immolation cascade led us to hypothesise that acidifying the system would hinder deprotonation of **4**, thereby interrupting the 1,4-elimination step and slowing or even pausing payload release mid-cascade. To establish proof-of-concept of switchable self-immolation, we employed model **1b** due to its moderately fast 1,4-elimination rate and its relatively long-lived 1*H*-triazole intermediate (Scheme 4). Palladium-activated self-immolation of **1b** with excess morpholine proceeded with rapid trigger removal followed by cyclisation–elimination. After completion of the cyclisation step (∼35 min), trifluoroacetic acid (TFA, 5 equiv.) was added, which immediately halted payload release and stabilised the concentrations of all species, confirming that the system had entered a paused state (Scheme 4b). Crucially, the concentration of intermediate **6b** remained stable at ∼9 mM for least 45 min, confirming that 1,4-elimination effectively ceases in the absence of base. ^1^H NMR analysis indicated that the 1*H*-triazole form of **4b** was the main dormant species, rather than its protonated triazolium congener (ESI, Section S8†), which is consistent with the poor basicity of 1,2,3-triazoles.^25^

Attempts to reactivate self-immolation by the addition of sub-stoichiometric Cs_2_CO_3_ ^26^ (2 × 2 equiv., arrows in Scheme 4b) produced only slight changes in the self-immolation rate. Only upon complete neutralization of the acid (6 equiv. total Cs_2_CO_3_) did the cascade return to its active state. In contrast to morpholine, Cs_2_CO_3_ facilitated more complete deprotonation of **4b** leading to formation of the triazolide anion, as evidenced in the ^1^H NMR spectra by the up-field shift of the aromatic triazole peak from ∼7.78 ppm to ∼7.73 ppm (ESI, Fig. S96†).

To ascertain if multiple switching was possible, a second aliquot of TFA (8 equiv.) was added after a further 30 min of self-immolation. Once again, the system entered its paused state, characterised by a plateau in the kinetics profiles of key species that lasted at least 55 min, until the addition of further Cs_2_CO_3_ (9.5 equiv.) returned the system to its active state (Scheme 4b). Upon completion of the cascade, the system reached a comparable degree of payload release to the non-switched experiments, indicating that switching between protonated and deprotonated states occurred cleanly and without appreciable deleterious side reactions.

Dynamic switching experiments were also conducted on models **1a** and **1e** in DMSO-*d*_6_ to study the degree of rate control in the ‘slow’ and ‘fast’ kinetics regimes. In both cases, addition of TFA (5 equiv.) to the activated linker systems drastically altered their self-immolation kinetics. Model **1a** exhibited a paused phase similar to **1b** that remained stable over ∼50 h (ESI, Fig. S94†). By contrast, **1e** slowed drastically but did not pause completely (ESI, Fig. S98†). This can be attributed to the instability of 1*H*-triazole intermediate **4e**, which undergoes rapid 1,4-elimination due to the stabilizing influence of its aromatic α-methine substituent. In both cases, neutralization of the acid with Cs_2_CO_3_ returned both systems to their original active phases to engender complete payload delivery. These results confirm that the rate of payload release can be influenced under all three kinetics regimes, with stable pausing achieved for **1a** and **1b**, and significant rate modulation without pausing for **1e**.

Finally, pH switching experiments were performed in DMSO-*d*_6_/D_2_O (8 : 2, v/v) using models **1a** and **1b**^27^ to demonstrate that the pausing mechanism could operate successfully in aqueous solvent mixtures. Switching was achieved by sequential additions of aqueous HCl and NaOH (20–30 equiv. each) to the reaction mixtures. Similar to their behaviour in neat DMSO, both model systems could be switched cleanly between active and paused phases multiple times during their cascade sequences (ESI, Section S9†). In both cases, stable paused phases were observed and final payload release efficiencies of >90% were achieved upon completion of the cascades, thus demonstrating that pH-mediated switching performs well in aqueous mixtures.

## Conclusions

In conclusion, we have demonstrated a mechanism for dynamically pausing and restarting an activated self-immolation cascade in response to pH changes. The pausing mechanism exploits the formation of a metastable 1*H*-triazole intermediate, whose deprotonation dictates the overall payload release profile. The system is remarkably stable under acidic conditions, allowing repeated switching in both organic and aqueous-organic solvent mixtures. The ability to dynamically regulate self-immolation using external signals has high relevance to ‘smart’ payload release systems featuring stimulus-responsive kinetics.^28^ Our findings thus highlight a new application of self-immolative triazoles, which have found remarkably little use in small molecule linker designs and are yet to feature in self-immolative polymers (SIPs). We believe the modularity of this system offers broad scope for developing new stimuli-responsive controlled-release systems, especially in materials that operate under mildly basic conditions such as paints, resins and surface coatings.

## Conflicts of interest

There are no conflicts to declare.

## Supplementary Material

SC-011-D0SC00532K-s001
