# Supplementary material for: Dynamic pH responsivity of triazole-based self-immolative linkers
Source: Chem Sci. 2020 Mar 3;11(14):3713–8. doi: 10.1039/d0sc00532k (PMC8152797; doi:10.1039/d0sc00532k)
Supplement: SC-011-D0SC00532K-s001 [file SC-011-D0SC00532K-s001.pdf]

Electronic Supplementary Material (ESI) for Chemical Science.
This journal is © The Royal Society of Chemistry 2020

Supporting Information for:

Dynamic pH responsivity of triazole-based self-immolative linkers

Derrick A. Roberts,*[,†,‡] Ben S. Pilgrim,[§] Tristan N. Dell,[⊥] and Molly M. Stevens*[,‡,⊥]

[†] Key Center for Polymers and Colloids, School of Chemistry, The University of Sydney, Sydney NSW 2006, Australia

[‡]Department of Medical Biochemistry and Biophysics, Karolinska Institutet, 171 77 Stockholm, Sweden

[§]School of Chemistry, The University of Nottingham, Nottingham NG7 2RD, UK

[⊥]Department of Materials, Department of Bioengineering, and Institute for Biomedical Engineering, Imperial College London, London SW7 2AZ, UK

E-mail: derrick.roberts@sydney.edu.au, m.stevens@imperial.ac.uk

Table of Contents

S1. Materials 2
S2. Analytical methods 2
S2.1. NMR Spectroscopy 2
S2.2. Low-resolution mass spectrometry 2
S2.3. High-resolution mass spectrometry 3
S2.4. FTIR 3
S2.5. Software 3
S2.6. Access to raw data 3
S3. Synthesis of Trigger Azide Precursor (9) 4
S3.1. Synthesis of allyl phenyl carbonate (S1) 4
S3.2. Synthesis of Compound 7 5
S3.3. Synthesis of Compound 8 6
S3.4. Synthesis of Compound 9 9
S4. Synthesis of Anisidine Propargyl Carbamates (10a–f) 12
S4.1. Synthesis of 3-substituted propargyl alcohols 12
S1.1.1. Characterisation data for 1-(3-fluorophenyl)prop-2-yn-1-ol (Compound S2) 12
S1.1.2. Characterisation data for 1-(3-methoxyphenyl)prop-2-yn-1-ol (Compound S3) 13
S4.2. Synthesis of Alkynes 10a-f 14
S1.1.3. Characterization data for Alkyne 10a 14
S1.1.4. Characterization data for Alkyne 10b 15
S1.1.5. Characterization data for Alkyne 10c 17
S1.1.6. Characterization data for Alkyne 10d 19
S1.1.7. Characterization data for Alkyne 10e 21
S1.1.8. Characterization data for Alkyne 10f 23
S5. Synthesis of Self-immolative Model Compounds (1a–f) 26
S5.1. Characterization data for Model 1a 26
S5.2. Characterization data for Model 1b 29
S5.3. Characterization data for Model 1c 31
S5.4. Characterization data for Model 1d 33
S5.5. Characterization data for Model 1e 35
S5.6. Characterization data for Model 1f 38
S6. Self-Immolation Kinetics of Models 1a–f in DMSO-d_6 40
S6.1. General method 40
S6.2. Kinetics methodology for model 1a 40
S6.3. Kinetics methodology for models 1b-f 41
S6.4. Kinetics analysis methodology 41
S6.5. Reference NMR spectra (reagents and products) 43
S6.6. Self-immolation control experiments for 1c 43
S6.7. Self-immolation kinetics data for 1a–f 44
S7. Self-Immolation Kinetics of Models 1a, 1b and 1d in DMSO-d_6/D_2O 59
S7.1. Self-immolation control data for 1a, 1b and 1d in DMSO-d_6/D_2O 59
S7.2. Self-immolation kinetics data for 1a, 1b and 1d in DMSO-d_6/D_2O 61
S8. Acid/base-mediated switching in DMSO-d_6 (Models 1a, 1b and 1e) 68
S9. Acid/base-mediated switching in DMSO-d_6/D_2O 8:2 (Models 1a and 1b) 74
S10. References 77

S1. Materials

Commercial solvents and reagents were used without further purification unless specified otherwise. Flash column chromatography was performed using silica gel high purity grade (pore size 60 Å, 230–400 mesh particle size, Fluorochem UK). TLC analyses were performed on Merck TLC silica gel 60 F254 aluminum-backed plates. Product spots were visualized under UV light (λ_{max} = 254 nm) and/or by staining with potassium permanganate dip. All anhydrous reactions were carried out in flame-dried (butane blowtorch) glassware dried under an inert atmosphere of N_2 provided by a double manifold. All reactions were stirred with Teflon-coated magnetic followers. Room temperature is taken as 293 K. Brine refers to a saturated aqueous solution of sodium chloride.

S2. Analytical methods

S2.1. NMR Spectroscopy

NMR spectra were recorded at the University of Cambridge using Bruker Avance DPX 400 MHz Avance III HD Smart Probe (VT-NMR, ^{19}F NMR and kinetics experiments) and 500 MHz TCI cryoprobe NMR spectrometers (high-resolution characterization). NMR spectra were also collected at The Karolinska Institute, Sweden, using a Bruker 400 MHz Ultrashield spectrometer. All spectrometers were automatically tuned and matched to the correct operating frequencies. For ^{1}H experiments requiring quantitative integration, 90° pulse calibration and T_1 estimations were performed to ensure complete relaxation between pulses. T_1 values were estimated by the inversion-recovery method using the standard Bruker pulse program *t1ir1d*. ^{1}H NMR kinetics experiments were carried out using a modified *zg30* pulse program (30° pulse) with a recycle delay (D1) of 2T_1. ^{1}H and ^{13}C NMR spectra are referenced to the residual solvent peak for DMSO-d_6 (^{1}H: 1.94 ppm, ^{13}C: 118.26 ppm) or $CDCl_3$ (^{1}H: 7.26 ppm, ^{13}C: 77.16 ppm), as appropriate. ^{19}F NMR spectra were referenced to hexafluorobenzene (−164.9 ppm), which was added directly to the NMR samples. Deuterated solvents were obtained from Fluorochem UK and Sigma Aldrich and used without any further purification.

Samples were prepared by centrifugation prior to analysis, or filtration through a glass fiber plug (~0.7 µm pore size) if suspended solids were present. NMR signals are reported in terms of chemical shift (δ) in parts-per-million (ppm), multiplicity, coupling constants (in Hz) and relative integral, in that order. The following abbreviations for multiplicity are used: s = singlet, d = doublet, t = triplet, q = quartet, quint = quintet, m = multiplet (denotes complex pattern), dd = doublet of doublets, dt = doublet of triplets, td = triplet of doublets, and br = broad signal. Spectra were digitally processed (phase and baseline corrections, integration, peak analysis) using Varian Topspin 3 and Mestrenova 14.0.1-23559 (Licensed to Dr. Derrick Roberts). Exponential window functions were applied to all 1D spectra at 0.3–2 Hz to digitally enhance signal-to-noise where required. 1D spectra were baseline corrected with the ablative (^{1}H) and Whittaker smoother (^{13}C) correction functions. Figures of spectra were exported as PDFs and edited in Adobe Illustrator CC 23.0.6 (Licensed for use by Dr Derrick Roberts through The University of Sydney, Australia).

S2.2. Low-resolution mass spectrometry

Low resolution electrospray ionization (ESI) mass spectra were obtained by routine LCMS analysis using an Agilent 1100 series Liquid Chromatography/Mass Selective Detector (LC/MSD) system fitted with an ACE3 C_8 column (50 × 3.00 mm) running a gradient of 10-90% CH_3CN in water containing 0.1% trifluoroacetic acid (CF_3COOH) at a flow rate of 1 mL/min on a 1.5 min analysis run. UV intensities were recorded at 220, 254 and 305 nm. ESI mass spectra were collected using an Agilent Technologies LC/MSD Trap VL (mass range m/z 50–1500) mass analyzer module. The instrument

was controlled, and LCMS traces subsequently processed using Agilent Technologies LC/MSD ChemStation software (1990-2003). This instrument was configured for communal/walk-up use and was maintained by Dr Birger Sjöberg, Senior Laboratory Manager at SciLifeLab within the Karolinska Institute, Sweden.

S2.3. High-resolution mass spectrometry

High resolution ESI mass spectra were obtained through the mass spectrometry facility at the Department of Chemistry, The University of Cambridge, using a Waters' Xevo G2-S bench top QTOF or a Waters' Vion IMS QTOF Ion Mobility Quadrupole Time-of-flight Mass Spectrometer, which were both calibrated to tolerances of 5.0 ppm (calibration regularly performed by the Mass Spectrometry Facility). Mass spectra were processed automatically using MassLynx 4.1.

S2.4. FTIR

FTIR spectra were recorded on a Perkin Elmer Spectrum One FTIR spectrometer (serial #59208), calibrated by Spectra Science Ltd. (Cambridge) on 17 October 2018. Spectra were collected and processed using Perkin Elmer Spectrum software.

S2.5. Software

All chemical structures were prepared using ChemDraw Professional 16.0.1.4 (61) for Mac, licensed for use by Dr Derrick Roberts through The University of Sydney. Figures were prepared using Adobe Illustrator CC 23.0.6, licensed for use by Dr Derrick Roberts through The University of Sydney, Australia. The manuscript and supporting information were prepared in Microsoft Word 365 and data were processed in Microsoft Excel 365 using a personal license to Dr Derrick Roberts. Kinetics data were fitted to monoexponential decay models using a trial version of IgorPro 7 for Mac.

S2.6. Access to raw data

All raw and processed spectroscopic data (NMR, LRMS, LCMS, FTIR), kinetics calculations and plotted data are available upon request to the corresponding authors.

S3. Synthesis of Trigger Azide Precursor (9)

S3.1. Synthesis of allyl phenyl carbonate (S1)

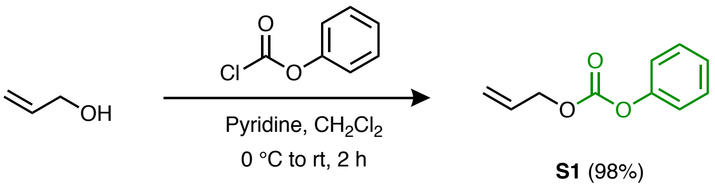

S1 (98%)

Allyl phenyl carbonate (**S1**) was synthesized according to a modified literature procedure.[1] A flame-dried 100 mL 2-necked flask fitted with a dropping funnel was charged with allyl alcohol (6.0 mL, 88 mmol), anhydrous pyridine (9.0 mL, 0.11 mol, 1.27 equiv.) and anhydrous CH_2Cl_2 (25 mL). The flask was cooled to 0 °C then phenyl chloroformate (11 mL, 88 mmol) was added dropwise over 30 min with vigorous stirring. After complete addition, a colorless solid had formed in the flask. The ice bath was removed and the reaction mixture stirred for a further 2 h. Ethyl acetate (200 mL) was added and the organic phase washed with water (100 mL), aqueous HCl (1 M; 3 × 50 mL), brine (100 mL), the organic layer dried over $MgSO_4$, filtered and the solvents removed by rotary evaporation. Allyl phenyl carbonate was obtained as a colorless liquid (15.3 g, 86 mmol, 98%) that was sufficiently pure to be used in the next reaction step. Characterization data were consistent with literature values.[1]

^{1}H NMR (400 MHz, $CDCl_3$) δ_H 7.43 – 7.35 (m, 2H), 7.28 – 7.22 (m, 1H), 7.22 – 7.16 (m, 2H), 6.01 (ddt, J = 17.2, 10.4, 5.9 Hz, 1H), 5.44 (dq, J = 17.2, 1.5 Hz, 1H), 5.34 (dq, J = 10.4, 1.2 Hz, 1H), 4.74 (dt, J = 5.9, 1.3 Hz, 2H). **LRMS** (+*ve* ESI-LCMS, CH_3CN/water/CF_3COOH) m/z 179.1 ($[M+H]^+$ 100%), 196.1 ($[M+NH_4]^+$, 45).

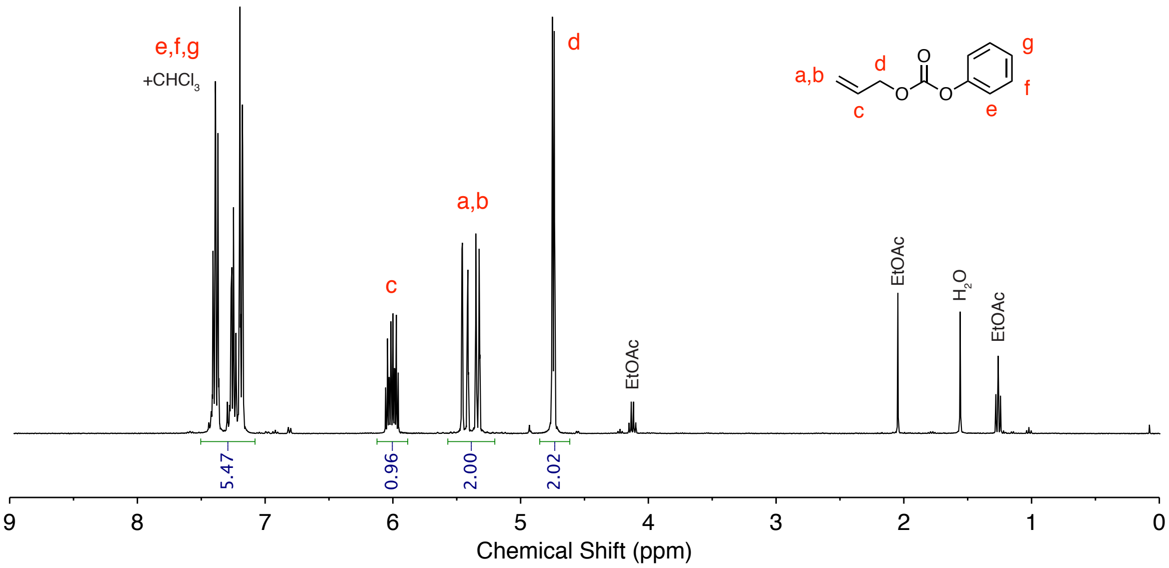

Figure S1. ^{1}H NMR spectrum (400 MHz, 298 K, $CDCl_3$) of compound **S1**, allyl phenyl carbonate.

S3.2. Synthesis of Compound 7

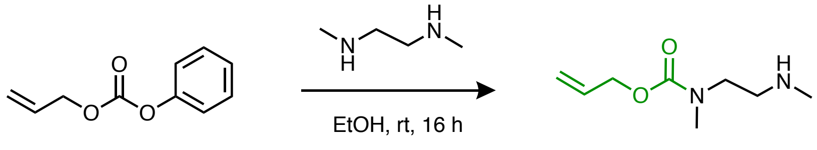

Compound **7** (*N*-allyloxycarbonyl-*N,N*-dimethyl-ethylenediamine) was synthesized according to a modified literature procedure.[2] A solution of allyl phenyl carbonate **S1** (6.1 g, 34 mmol) in EtOH (50 mL) was added dropwise to a solution of *N,N*-dimethylethylenediamine (6.0 mL, 56 mmol) in EtOH (230 mL). After stirring the reaction mixture at room temperature overnight (~16 h), the solvent was removed by rotary evaporation and the residue re-dissolved in water (200 mL). The solution was adjusted to ~pH 3 by addition of aqueous HCl (1 M) and the aqueous phase was extracted with CH_2Cl_2 (4 × 100 mL) to remove any doubly-protected amine. These initial organic extracts were discarded. The aqueous layer was then adjusted to ~pH 14 with the addition of aqueous NaOH (1 M) and extracted with CH_2Cl_2 (4 × 100 mL) to isolate monoprotected compound **7**, leaving any unreacted *N,N*-dimethylethylenediamine in the alkaline aqueous phase. The organic extracts containing **7** were washed again with aqueous NaOH (2 M, 2 × 200 mL), dried over Na_2SO_4 and the solvent removed by rotary evaporation to furnish compound **7** as a colorless oil (4.2 g, 24 mmol, 71%) that was sufficiently pure to be used in the next reaction step. Characterization data were consistent with published values.[2] **^{1}H NMR** (400 MHz, $CDCl_3$) δ_H 5.92 (ddt, J = 17.3, 10.7, 5.5 Hz, 1H), 5.28 (dq, J = 17.2, 1.6 Hz, 1H), 5.18 (dq, J = 10.4, 1.3 Hz, 1H), 4.57 (dt, J = 5.5, 1.5 Hz, 2H), 3.39 (t, J = 6.5 Hz, 2H), 2.93 (s, 3H), 2.83 – 2.68 (m, 2H), 2.43 (s, 3H). **LRMS** (+*ve* ESI-LCMS, CH_3CN/water/CF_3COOH) *m/z* 173.1 ($[M+H]^+$ 100%).

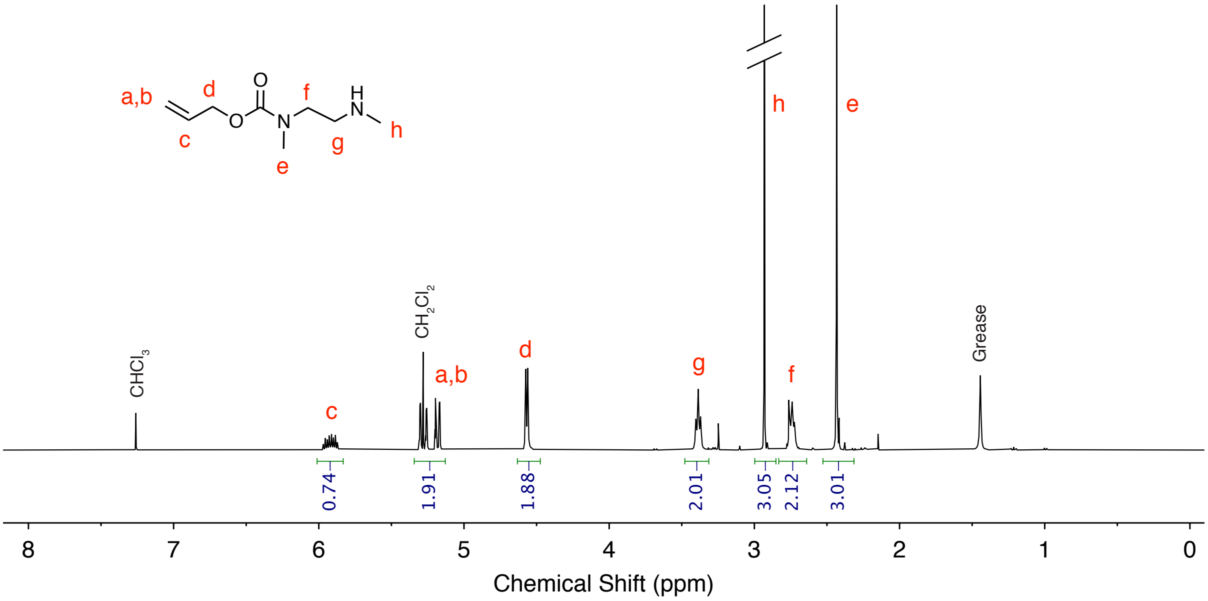

Figure S2. ^{1}H NMR spectrum (400 MHz, 298 K, $CDCl_3$) of compound **7**.

S3.3. Synthesis of Compound 8

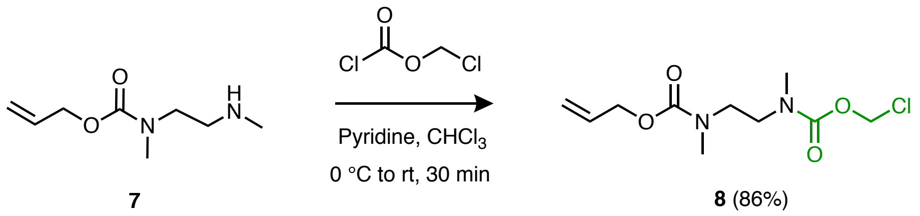

A flame-dried 100 mL flask fitted with a dropping funnel was charged with compound **7** (5.42 g, 31.4 mmol), anhydrous pyridine (3.05 mL, 37.7 mmol) and anhydrous $CHCl_3$ (60 mL). The flask was cooled to 0 °C and chloromethyl chloroformate (3.35 mL, 37.7 mmol) was added dropwise with stirring over 15 min. The reaction mixture changed from colorless to deep yellow upon complete addition. The ice bath was removed, and the reaction stirred for a further 30 min, monitoring by TLC (hexane/EtOAc = 7:3; product $R_f \sim 0.3$). After complete consumption of the starting material, the reaction was quenched with water (50 mL) and the chloroform layer isolated. The aqueous layer was extracted with CH_2Cl_2 (3 × 30 mL) and the combined organic extracts washed with sat. $NaHCO_3$ (3 × 100 mL), brine (100 mL), dried over $MgSO_4$, filtered and the solvents removed by rotary evaporation. Compound **8** was obtained as a pale-yellow oil (7.18 g, 27.1 mmol, 86%) that required no further purification.

^{1}H NMR (500 MHz, $CDCl_3$) δ_H 6.02 – 5.82 (m, 1H), 5.82 – 5.66 (m, 2H), 5.33 – 5.10 (m, 2H), 4.68 – 4.40 (m, 2H), 3.55 – 3.33 (m, 4H), 3.04 – 2.86 (m, 6H). **^{13}C NMR** (126 MHz, $CDCl_3$) δ_C 156.90 – 155.54, 154.66 – 152.56, 133.95 – 131.49, 118.86 – 115.90, 72.01 – 69.23, 67.33 – 64.98, 50.08 – 44.76, 36.90 – 32.50. **FTIR** (ATR, liquid film) ν_{max} 2939, 1722, 1695, 1649, 1472, 1400, 1290, 1260, 1206, 1162, 1116, 1073, 973, 928, 762, 696 cm^{-1}. **LRMS** (+*ve* ESI-LCMS, CH_3CN/water/CF_3COOH) *m*/*z* 265.0 ($[M+H]^+$ 100%). **HRMS** (TOF MS ASAP +*ve*) *m*/*z* calculated for $C_{10}H_{18}N_2O_4[^{35}Cl]$ 265.0955, found 265.0959.

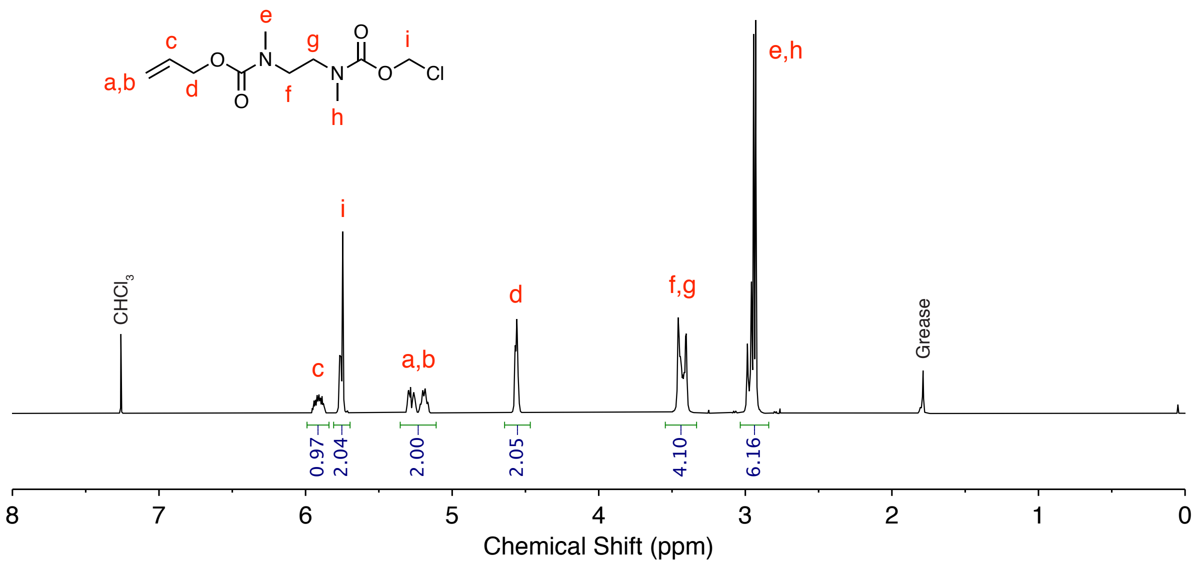

Figure S3. ^{1}H NMR spectrum (500 MHz, 298 K, $CDCl_3$) of compound **8**.

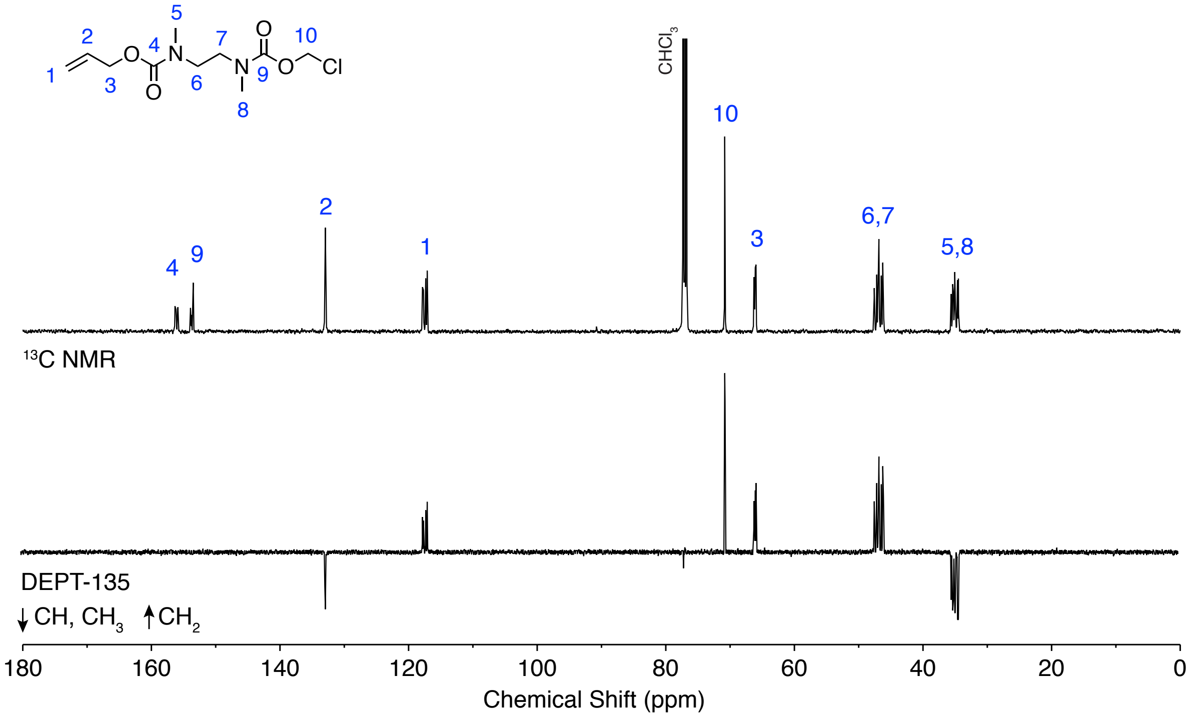

Figure S4. ^{13}C NMR and DEPT-135 spectra (126 MHz, 298 K, $CDCl_3$) of compound **8**.

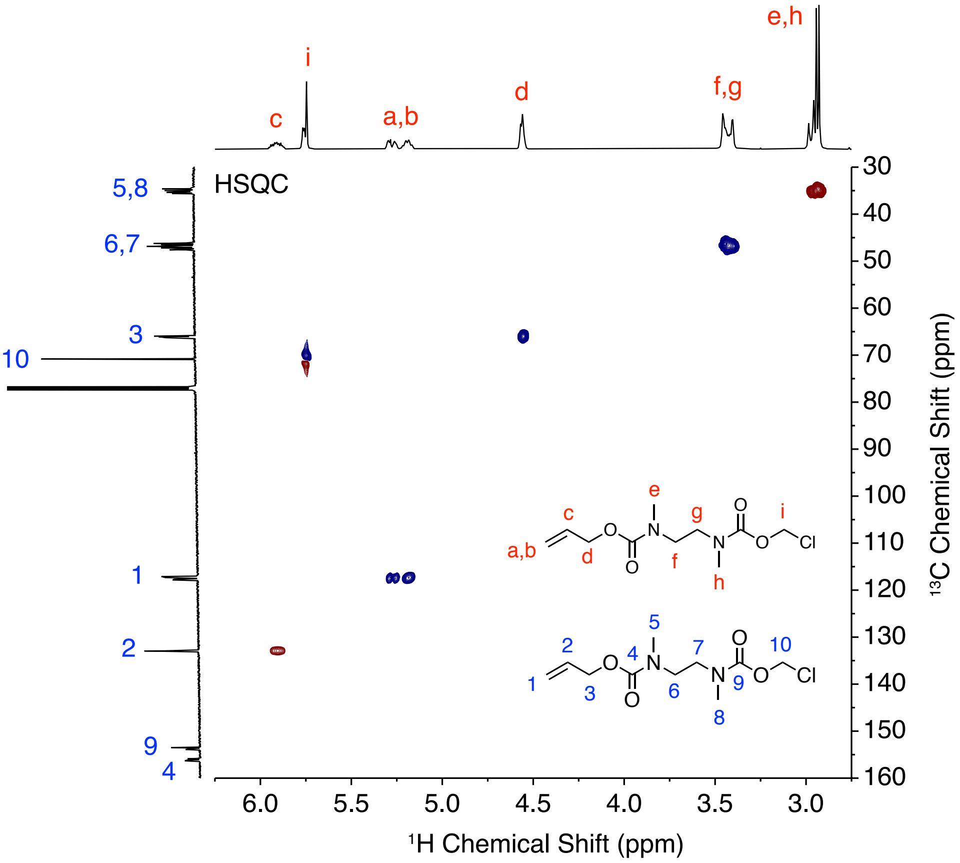

Figure S5. ^{1}H–^{13}C phase-sensitive HSQC spectrum (500/126 MHz, 298 K, $CDCl_3$) of compound **8**. Blue contours denote CH_2 groups and red contours denote CH and CH_3 groups. ^{1}H environments are assigned with letters, and ^{13}C environments are assigned with numbers.

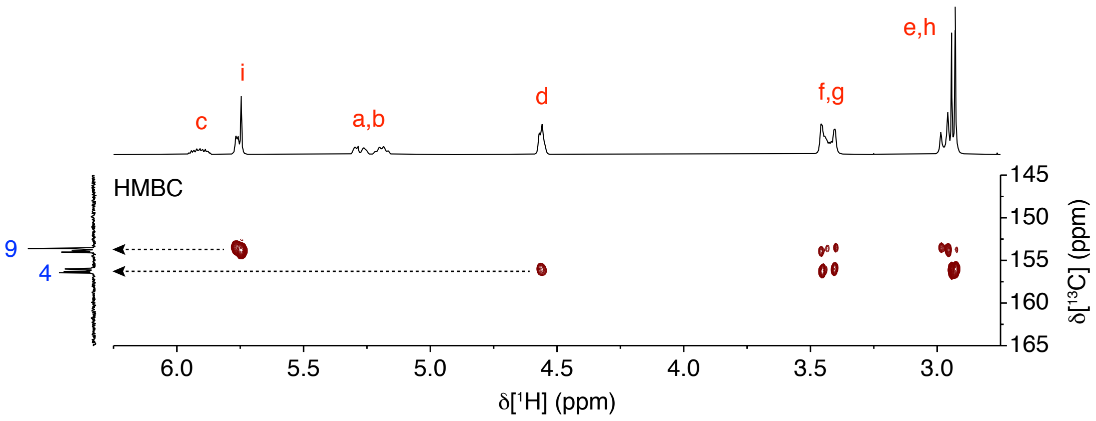

Figure S6. Partial ^{1}H–^{13}C HMBC spectrum (500/126 MHz, 298 K, $CDCl_3$) of compound **8**, confirming the assignment of carbons C9 and C4.

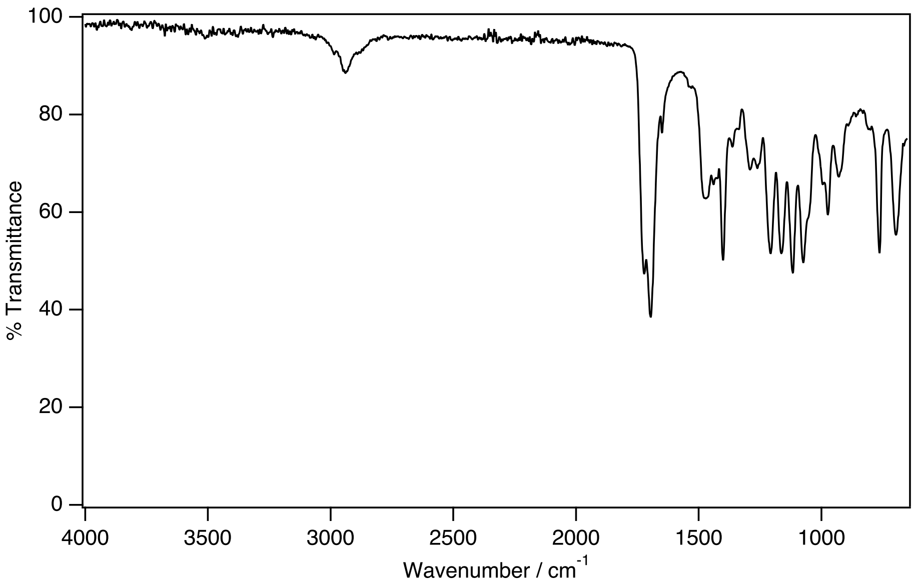

Figure S7. FTIR (ATR, liquid film) spectrum of compound **8**.

Monoisotopic Mass, Even Electron Ions
3 formula(e) evaluated with 1 results within limits (all results (up to 1000) for each mass)
Elements Used:
C: 0-10 H: 1-18 N: 1-2 O: 3-4 Cl: 1-1

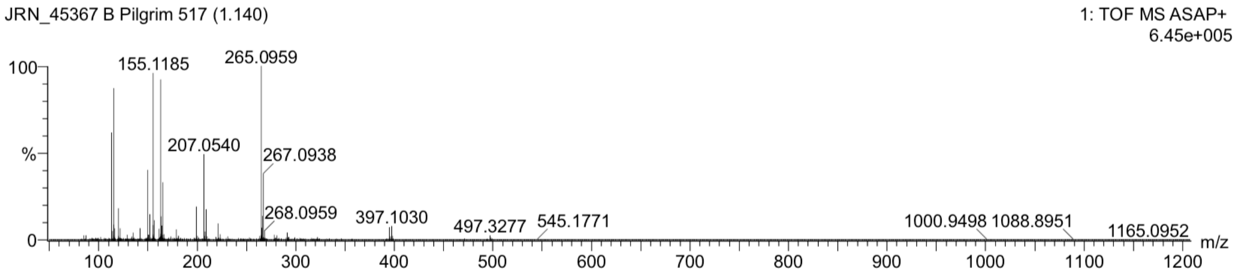

Minimum:				-1.5				
Maximum:		5.0	5.0	50.0				
Mass	Calc. Mass	mDa	PPM	DBE	i-FIT	Norm	Conf(%)	Formula
265.0959	265.0955	0.4	1.5	2.5	1558.1	n/a	n/a	C10 H18 N2 O4 Cl

Figure S8. HRMS spectrum (TOF MS ASAP +*ve*) and analysis report for compound **8**.

S3.4. Synthesis of Compound 9

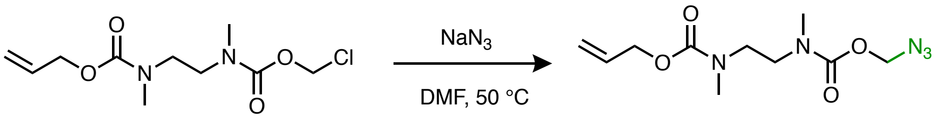

Sodium azide (121 mg, 1.87 mmol) was added to a solution of compound **8** (471 mg, 1.78 mmol) in DMF (2 mL). The reaction mixture was heated at 50 °C for 1.5 h (NB: chloride **8** and azide **9** have coincident retention factors in hexane/ethyl acetate mixtures. Optimal reaction time was determined by ^{1}H NMR reaction monitoring. Extended reaction times led to decomposition of the azide). Upon completion, the reaction mixture was diluted with EtOAc (30 mL) and washed with water (3 × 30 mL), brine (30 mL), dried over Na_2SO_4 then filtered and solvents removed to furnish azide **9** as a pale-yellow oil (463 mg, 1.71 mmol, 96%) that was sufficiently pure to be used for CuAAC coupling.

^{1}H NMR (500 MHz, $CDCl_3$) δ_H 5.93 (ddt, *J* = 17.3, 10.8, 5.6 Hz, 1H), 5.36 – 5.26 (m, 1H), 5.25 – 5.18 (m, 1H), 5.18 – 5.11 (m, 2H), 4.59 (dt, *J* = 5.7, 1.5 Hz, 2H), 3.56 – 3.34 (m, 4H), 3.07 – 2.90 (m, 6H). **^{13}C NMR** (101 MHz, $CDCl_3$) δ_C 158.05 – 154.24 (m), 135.26 – 130.20 (m), 118.75 – 114.84 (m), 76.06 – 75.05 (m), 67.98 – 65.50 (m), 47.68 – 43.33 (m), 36.22 – 33.74 (m). **FTIR** (ATR, liquid film) ν_{max} 2943, 2106 (N_3 stretch), 1694, 1477, 1402, 1293, 1240, 1205, 1162, 1117, 1038, 966, 913, 764 cm^{-1}. **LRMS** (+*ve* ESI-LCMS, CH_3CN/water/CF_3COOH) *m*/*z* 173.1 ($[M-C_2N_3O_2]^+$ 100%), 272.1 ($[M+H]^+$, 20). **HRMS** (TOF MS ASAP +*ve*) *m*/*z* calculated for $C_{10}H_{17}N_5O_4Na$ 294.1178, found 294.1180.

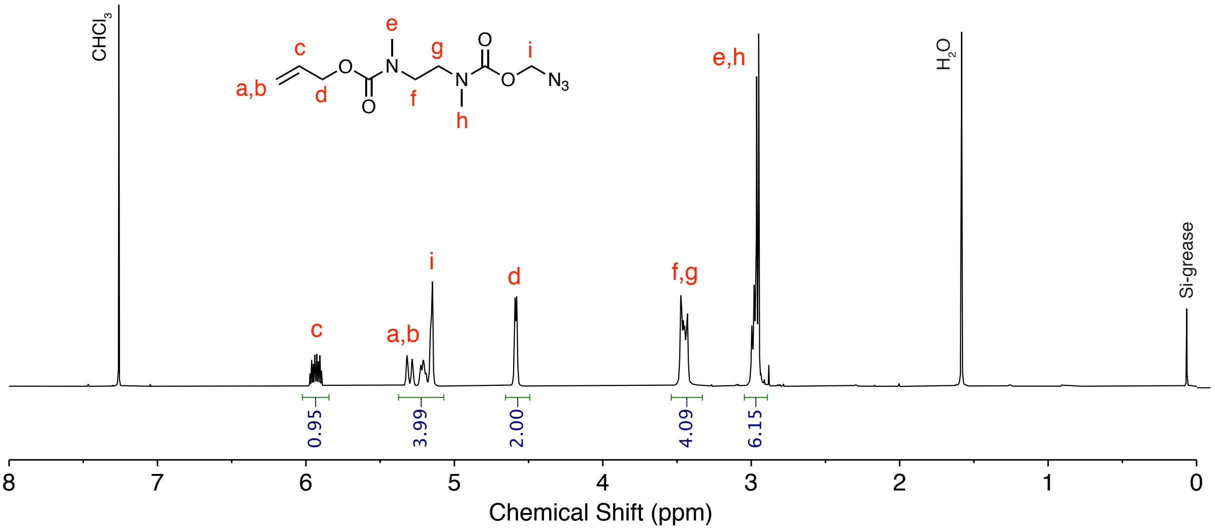

Figure S9. ^{1}H NMR spectrum (500 MHz, 298 K, $CDCl_3$) of compound **9**.

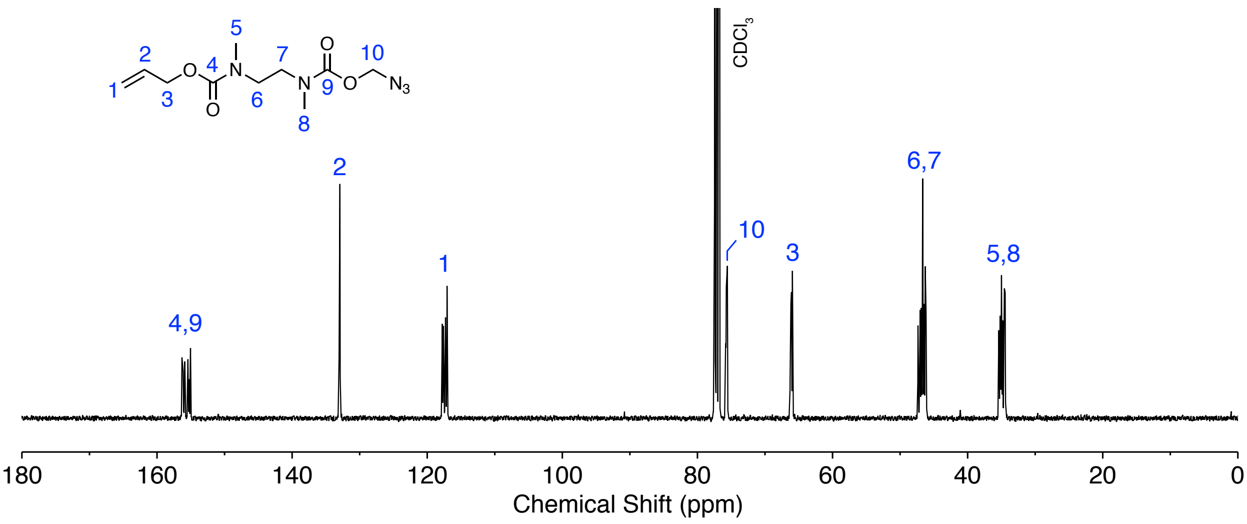

Figure S10. ^{13}C NMR spectrum (101 MHz, 298 K, $CDCl_3$) of compound **9**.

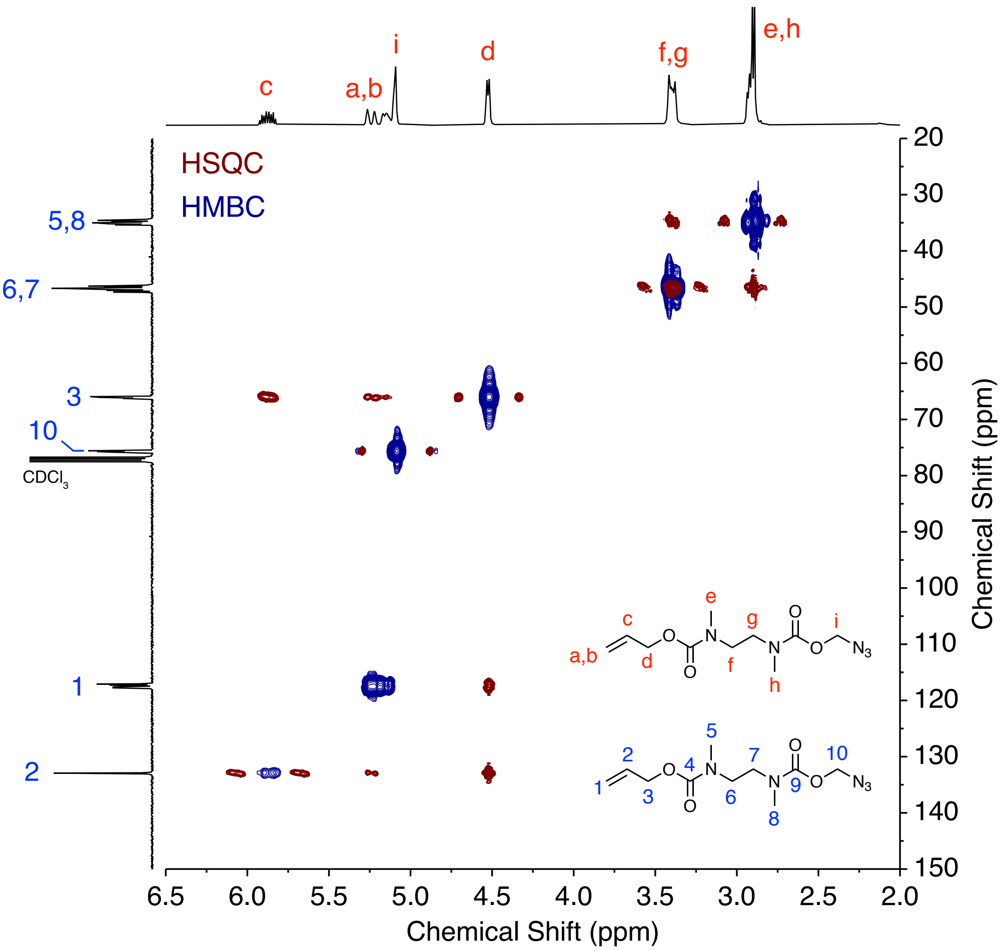

Figure S11. Overlaid ^{1}H–^{13}C HSQC/HMBC spectra (400/101 MHz, 298 K, $CDCl_3$) of compound **9**.

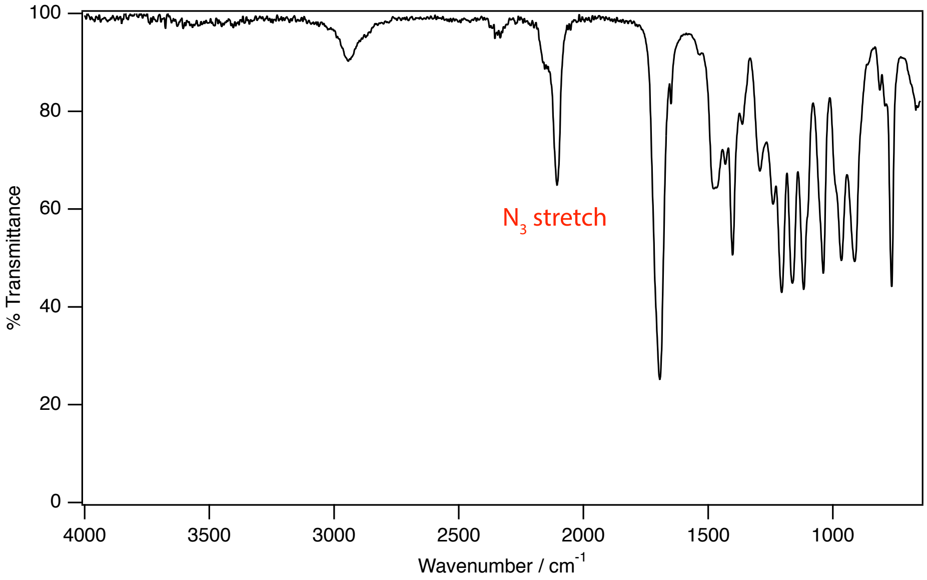

Figure S12. FTIR spectrum of compound **9**, with the characteristic azide stretching frequency highlighted.

Monoisotopic Mass, Even Electron Ions
58 formula(e) evaluated with 1 results within limits (all results (up to 1000) for each mass)
Elements Used:
C: 0-10 H: 1-17 N: 0-5 O: 0-4 Na: 0-1

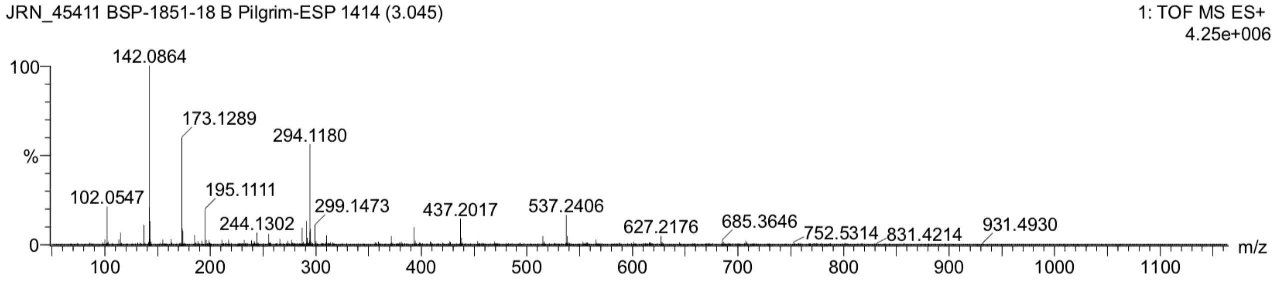

Minimum:				-1.5				
Maximum:		5.0	5.0	50.0				
Mass	Calc. Mass	mDa	PPM	DBE	i-FIT	Norm	Conf(%)	Formula
294.1180	294.1178	0.2	0.7	4.5	1667.5	n/a	n/a	C10 H17 N5 O4 Na

Figure S13. HRMS spectrum (TOF MS ASAP +*ve*) and analysis report for compound **9**.

S4. Synthesis of Anisidine Propargyl Carbamates (10a–f)

S4.1. Synthesis of 3-substituted propargyl alcohols

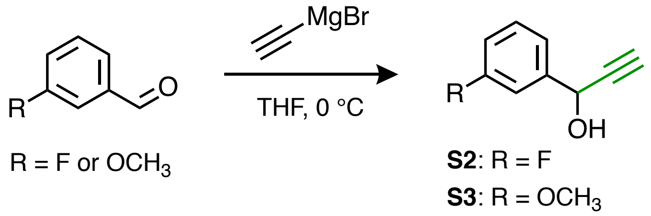

Figure S14. General scheme for the synthesis of 3-substituted propargyl alcohols **S2** and **S3**.

General procedure:

Substituted propargyl alcohols were synthesized according to a literature procedure.[3] A solution of ethynylmagnesium bromide (0.5 M in THF, 26 mL, 13 mmol) was added at 0 °C to a solution of the corresponding benzaldehyde (10 mmol) in THF (20 mL). After stirring at room temperature for 2 h, a saturated solution of NH_4Cl (20 mL) was added to the solution and the THF was removed by rotary evaporation. The aqueous phase was extracted three times with ethyl acetate and the organic layers were washed with water and brine and then dried over Na_2SO_4. After evaporation of the solvent, the resulting crude product was purified by column chromatography (hexane/EtOAc = 8:2) to give the 3-substituted propargyl alcohol.

S1.1.1. Characterisation data for 1-(3-fluorophenyl)prop-2-yn-1-ol (Compound S2)

Data were consistent with literature values.[4] **^{1}H NMR** (400 MHz, $CDCl_3$) δ_H 7.44 – 7.22 (m, 3H), 7.16 – 6.97 (m, 1H), 5.47 (d, J = 2.3 Hz, 1H), 2.70 (d, J = 2.3 Hz, 1H), 2.59 (s, 1H). **^{19}F NMR** (376 MHz, $CDCl_3 + C_6F_6$) δ_F −115.61. **LRMS** (+*ve* ESI-LCMS, CH_3CN/water/CF_3COOH) *m/z* 133.1 ([M-OH]$^+$ 100).

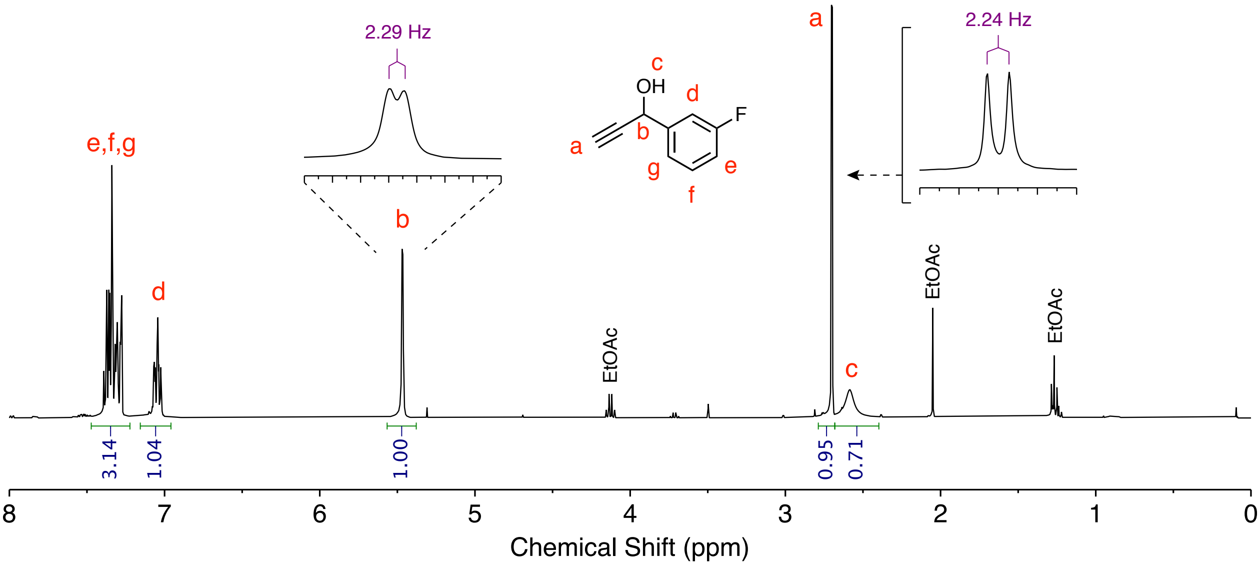

Figure S15. ^{1}H NMR spectrum (400 MHz, 298 K, $CDCl_3$) of compound **S2**.

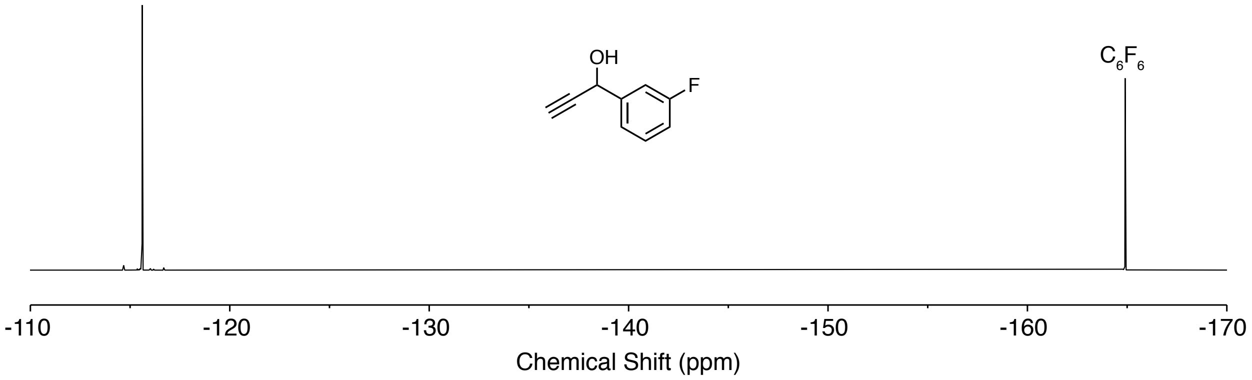

Figure S16. ^{19}F NMR spectrum (376 MHz, 298 K, $CDCl_3$) of compound **S2**.

S1.1.2. Characterisation data for 1-(3-methoxyphenyl)prop-2-yn-1-ol (Compound S3)

Data were consistent with literature values.[5] **^1H NMR** (400 MHz, $CDCl_3$) δ_H 7.35 – 7.28 (m, 1H), 7.17 – 7.09 (m, 2H), 6.88 (ddd, *J* = 8.3, 2.6, 1.0 Hz, 1H), 5.44 (d, *J* = 2.3 Hz, 1H), 3.83 (s, 3H), 2.67 (d, *J* = 2.3 Hz, 1H), 2.39 (br s, 1H). **LRMS** (+*ve* ESI-LCMS, CH_3CN/water/CF_3COOH) *m*/*z* 145.1 ($[M-OH]^+$ 100).

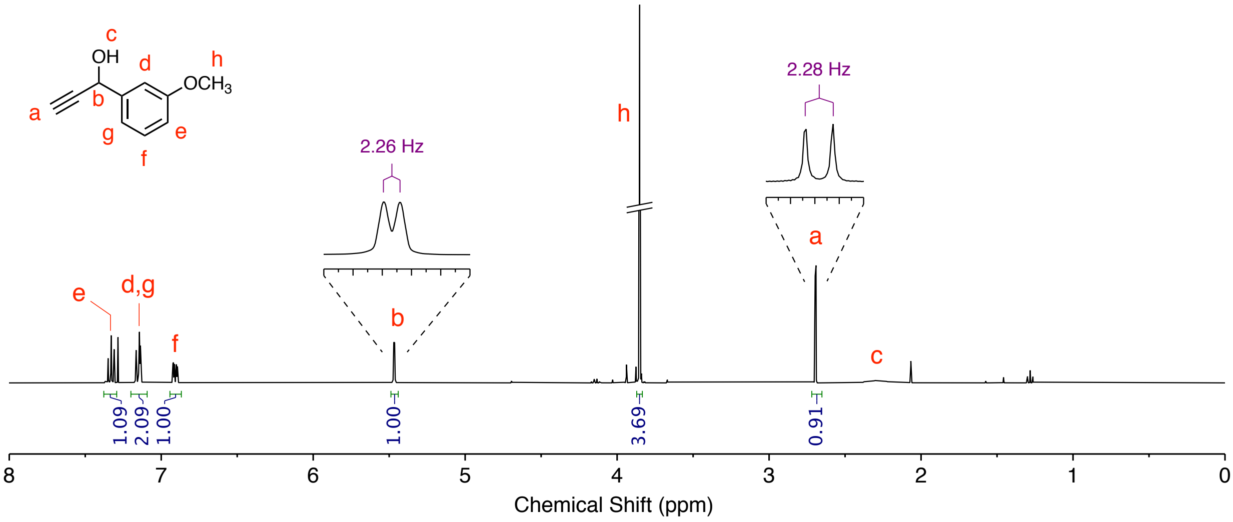

Figure S17. ^1H NMR spectrum (400 MHz, 298 K, $CDCl_3$) of compound **S3**.

S4.2. Synthesis of Alkynes 10a-f

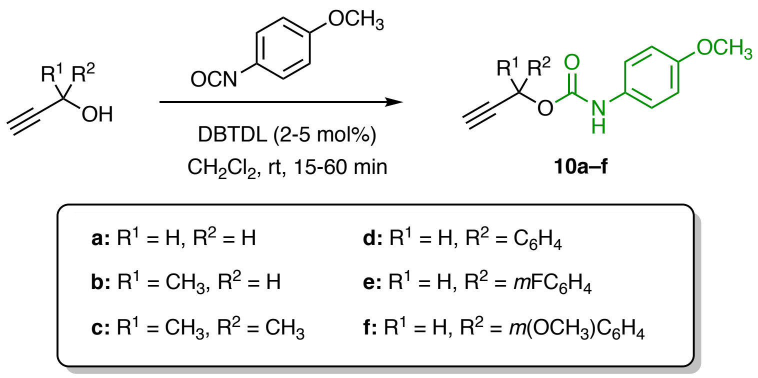

Scheme S1. General scheme for the synthesis of alkynes **10a–f**. DBTDL refers to dibutyltin dilaurate.

Representative procedure:

A dry reaction vessel containing a magnetic follower was charged with propargyl alcohol (3.7 mmol, 1.1 equiv.), dibutyltin dilaurate (70 µmol; typically, 2-5 mol%) and anhydrous CH_2Cl_2 (5 mL). The solution was cooled to 0 °C, sparged with N_2 for 5 min, then 4-methoxyisocyanate (3.4 mmol, 1 equiv.) was added dropwise at 0 °C. The cold bath was removed and the reaction mixture stirred at room temperature, monitoring by TLC. Upon completion (typically <2 h), the reaction was quenched by addition of saturated NH_4Cl solution (5 mL), diluted with CH_2Cl_2 (30 mL), washed with water (3 × 30 mL), brine (30 mL), the organic layer dried over Na_2SO_4, filtered and the solvents removed to afford the crude product. Purification by silica chromatography using hexane/EtOAc (see details below) afforded the purified product.

S1.1.3. Characterization data for Alkyne 10a

After purification by silica gel chromatography in hexane/EtOAc 7:3 to 1:1, alkyne **10a** was obtained as a pale yellow solid (685 mg, 3.34 mmol, 97%). Characterisation data were consistent with reported values.[6] **^{1}H NMR** (400 MHz, DMSO-d_6) δ_H 9.65 (s, 1H), 7.35 (d, J = 8.4 Hz, 2H), 6.87 (d, J = 9.1 Hz, 2H), 4.73 (d, J = 2.4 Hz, 2H), 3.70 (s, 3H), 3.55 (t, J = 2.4 Hz, 1H). **LRMS** (+*ve* ESI-LCMS, CH_3CN/water/CF_3COOH) m/z 206.0 ($[M+H]^+$ 100%).

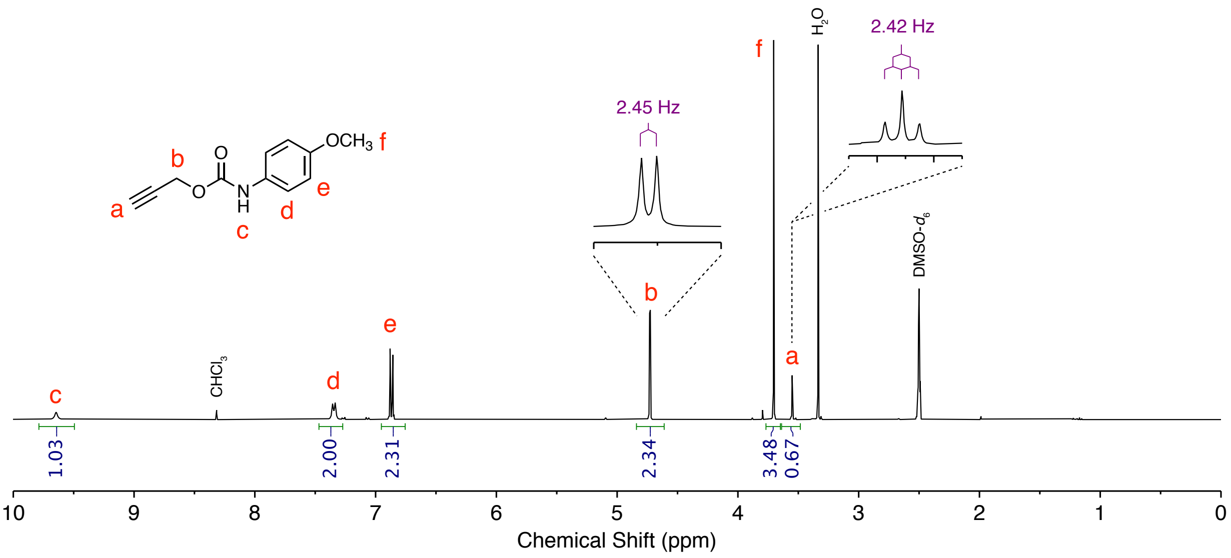

Figure S18. ^{1}H NMR spectrum (400 MHz, 298 K, DMSO-d_6) of compound **10a**.

S1.1.4. Characterization data for Alkyne 10b

After purification by silica gel chromatography in hexane/EtOAc 9:1 to 8:2, alkyne **10b** was obtained as a pale yellow microcrystalline solid (693 mg, 3.16 mmol, 93%).

^{1}H NMR (500 MHz, $CDCl_3$) δ_H 7.29 (d, *J* = 8.6 Hz, 2H), 6.84 (d, *J* = 9.1 Hz, 2H), 6.67 (s, 1H), 5.48 (qd, *J* = 6.7, 2.1 Hz, 1H), 3.77 (s, 3H), 2.49 (d, *J* = 2.2 Hz, 1H), 1.55 (d, *J* = 6.8 Hz, 3H). **^{13}C NMR** (126 MHz, $CDCl_3$) δ_C 156.19, 152.71, 130.72, 120.83, 114.35, 82.46, 73.18, 61.03, 55.59, 21.63. **FTIR** (ATR, solid powder) ν_{max} 3306, 2991, 2939, 2120 (weak, C≡C stretch), 1701, 1599, 1536, 1512, 1416, 1315, 1232, 1177, 1112, 1092, 1054, 1025, 932, 919, 867, 810, 777, 765, 735 cm^{-1}. **LRMS** (+*ve* ESI-LCMS, CH_3CN/water/CF_3COOH) *m*/*z* 220.0 ($[M+H]^+$ 100%). **HRMS** (TOF MS ASAP +*ve*) *m*/*z* calculated for $C_{12}H_{14}NO_3$ 220.0974, found 220.9070.

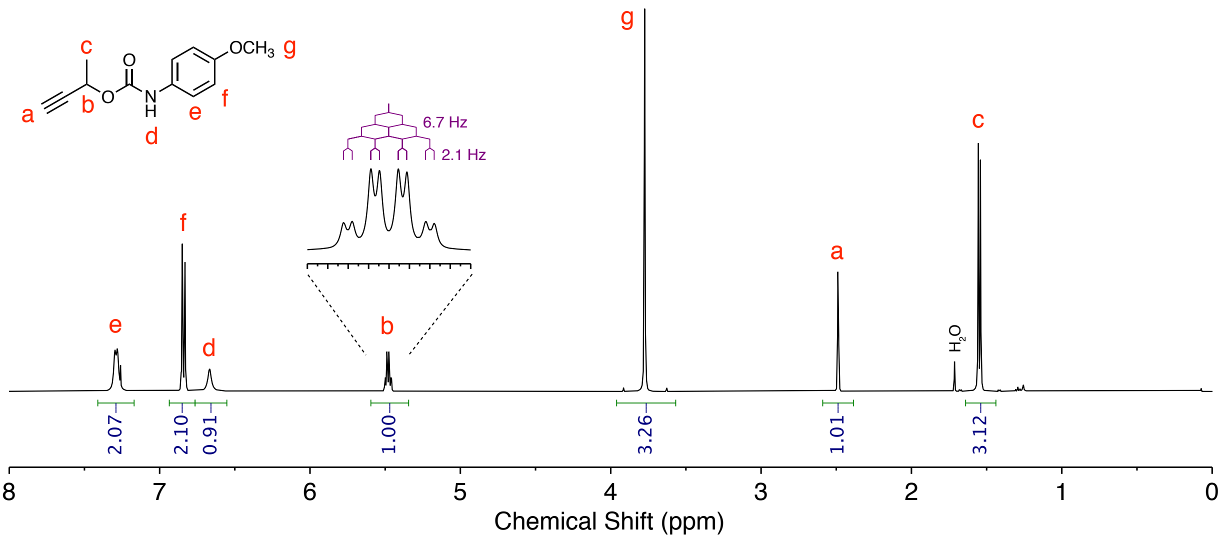

Figure S19. ^{1}H NMR spectrum (500 MHz, 298 K, $CDCl_3$) of compound **10b**.

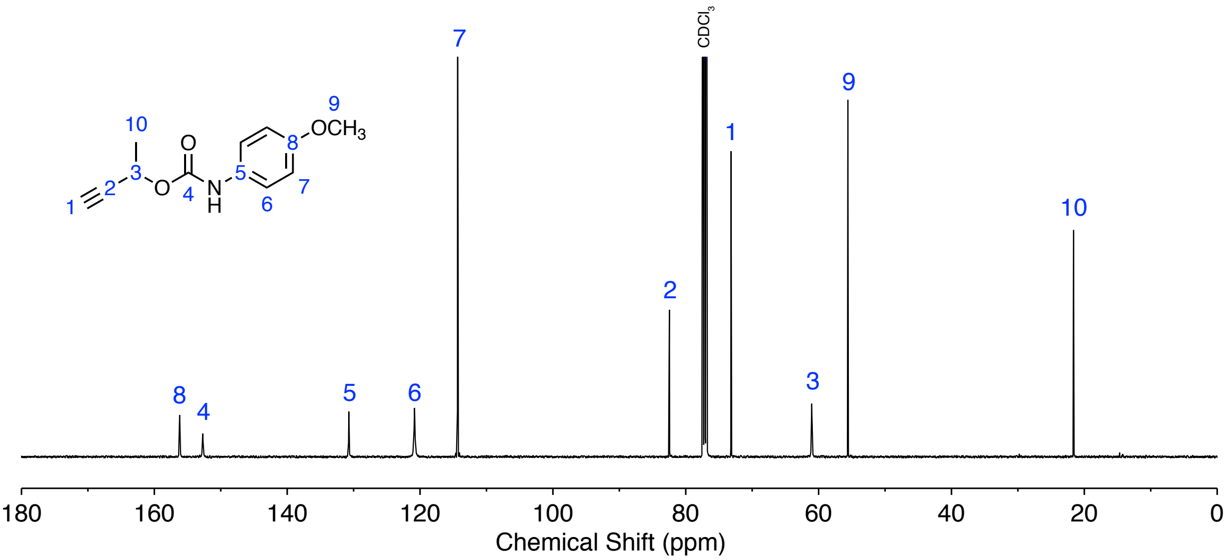

Figure S20. ^{13}C NMR spectrum (126 MHz, 298 K, $CDCl_3$) of compound **10b**.

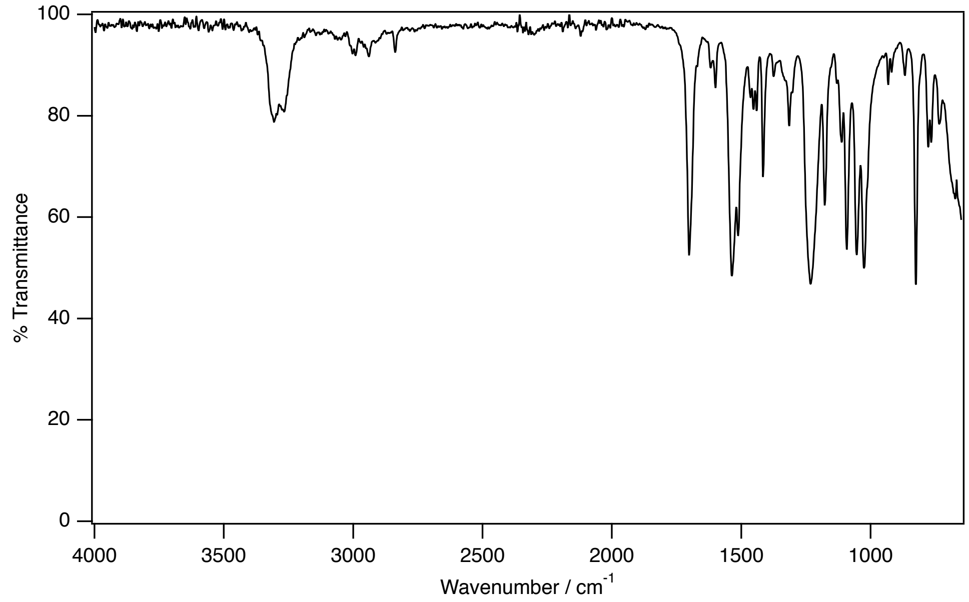

Figure S21. FTIR spectrum (ATR, solid powder) of compound **10b**. The alkyne stretching frequency (~2100 cm^{-1}) was observed to be extremely weak by this technique.

Monoisotopic Mass, Even Electron Ions
3 formula(e) evaluated with 1 results within limits (up to 50 closest results for each mass)
Elements Used:
C: 1-12 H: 1-14 B: 0-1 N: 1-1 O: 1-3
JRN_45574 B Pilgrim
JRN_45574 B Pilgrim 1694 (3.638) Cm (1669:1892)

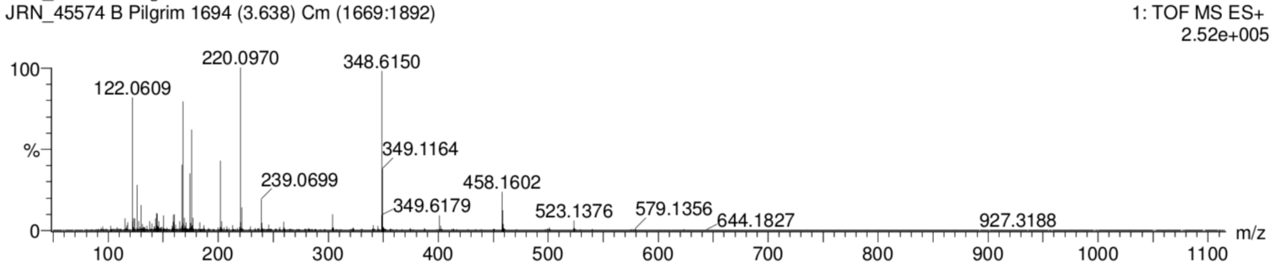

Minimum:				-1.5				
Maximum:		5.0	5.0	50.0				
Mass	Calc. Mass	mDa	PPM	DBE	i-FIT	Norm	Conf(%)	Formula
220.0970	220.0974	-0.4	-1.8	6.5	1044.2	n/a	n/a	C12 H14 N O3

Figure S22. HRMS spectrum (TOF MS ASAP +*ve*) and analysis report for compound **10b**.

S1.1.5. Characterization data for Alkyne 10c

After purification by silica gel chromatography in hexane/EtOAc 8:2 to 6:4, alkyne **10c** was obtained as pale-yellow fluffy needles (1.93 g, 8.3 mmol, 97%).

^{1}H NMR (400 MHz, $CDCl_3$) δ_H 7.30 (d, J = 8.3 Hz, 2H), 6.95 – 6.70 (m, 2H), 6.46 (s, 1H), 3.77 (t, J = 1.4 Hz, 3H), 2.57 (s, 1H), 1.74 (s, 6H). **^{13}C NMR** (101 MHz, $CDCl_3$) δ_C 156.02, 152.13, 131.03, 120.60, 114.32, 85.15, 72.40, 72.20, 55.63, 29.36. **FTIR** (ATR, solid powder) ν_{max} 3350, 3267, 2337, 2159 (weak, C≡C stretch), 1705, 1597, 1540, 1469, 1414, 1311, 1297, 1264, 1225, 1182, 1136, 1055, 1026, 952, 829, 780, 755, 704 cm^{-1}. **LRMS** (+*ve* ESI-LCMS, CH_3CN/water/CF_3COOH) *m*/*z* 489.2 ($[2M+Na]^+$ 10%), 234.1.2 ($[M+H]^+$ 15), 168.0 ($[M-C_5H_5]^+$ 100). **HRMS** (TOF MS ASAP +*ve*) *m*/*z* calculated for $C_{13}H_{16}NO_3$ 234.1130, found 234.1134.

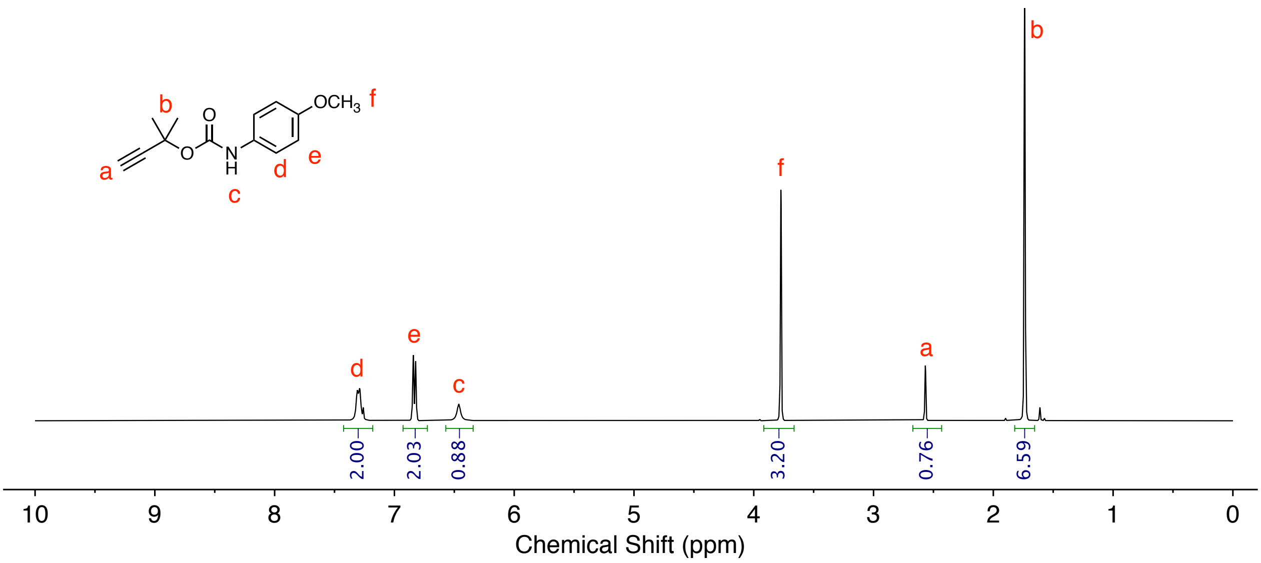

Figure S23. ^{1}H NMR spectrum (400 MHz, 295 K, $CDCl_3$) of compound **10c**.

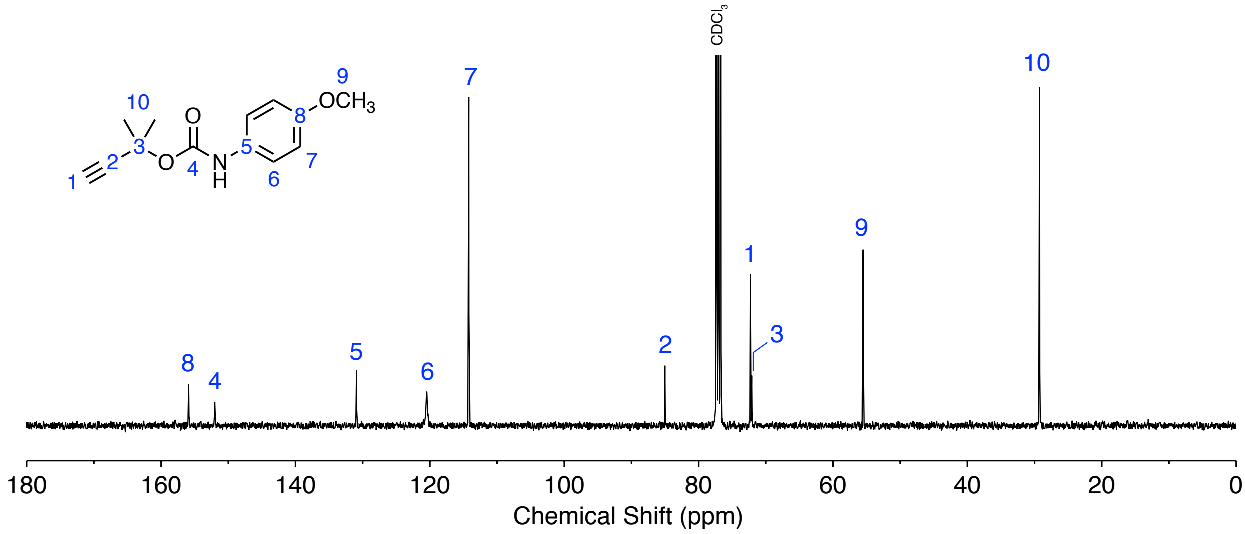

Figure S24. ^{13}C NMR spectrum (101 MHz, 295 K, $CDCl_3$) of compound **10c**.

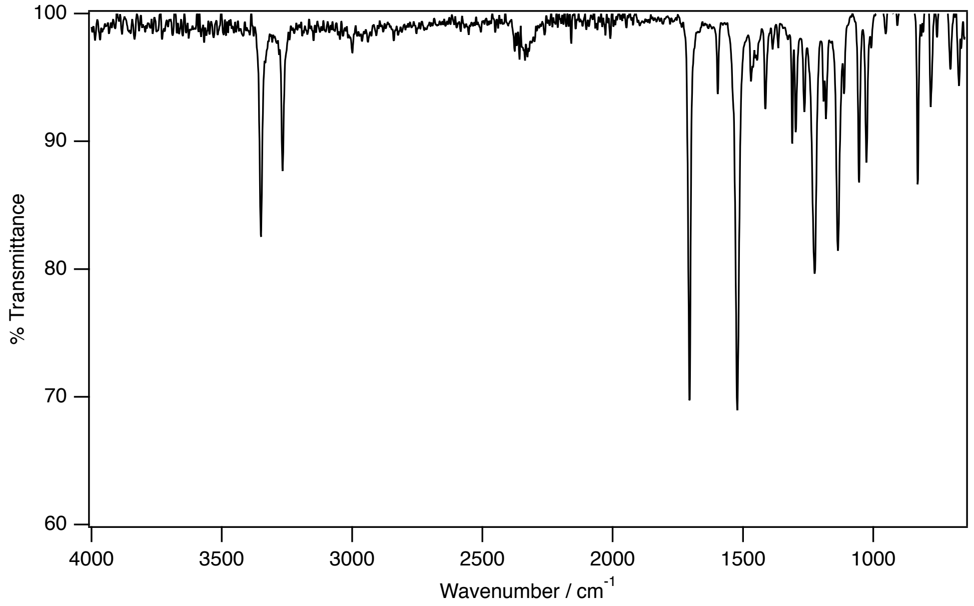

Figure S25. FTIR spectrum (ATR, solid powder) of compound **10c**. The alkyne stretching frequency (~2100 cm^{-1}) was observed to be extremely weak by this technique.

Single Mass Analysis
Tolerance = 5.0 PPM / DBE: min = -1.5, max = 50.0
Element prediction: Off
Number of isotope peaks used for i-FIT = 3

Monoisotopic Mass, Even Electron Ions
8 formula(e) evaluated with 1 results within limits (up to 50 closest results for each mass)
Elements Used:
C: 1-17 H: 1-16 B: 0-1 N: 1-1 O: 1-3
JRN_45340 B Pilgrim
JRN_45340 B Pilgrim 861 (1.874) Cm (851:1026) 1: TOF MS ASAP+ 1.28e+006

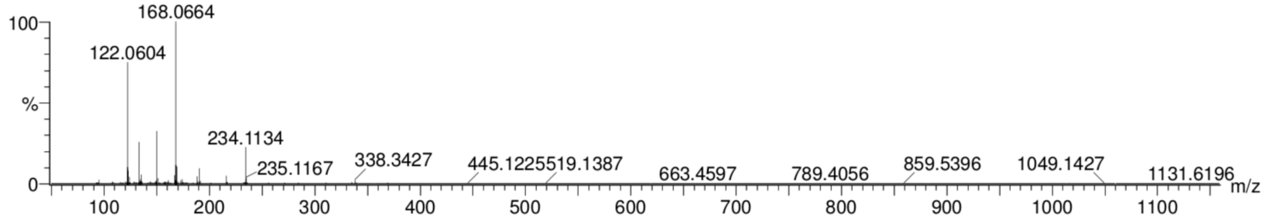

Minimum:				-1.5				
Maximum:		5.0	5.0	50.0				
Mass	Calc. Mass	mDa	PPM	DBE	i-FIT	Norm	Conf(%)	Formula
234.1134	234.1130	0.4	1.7	6.5	1852.5	n/a	n/a	C13 H16 N O3

Figure S26. HRMS spectrum (TOF MS ASAP +*ve*) and analysis report for compound **10c**.

S1.1.6. Characterization data for Alkyne 10d

After purification by silica gel chromatography in hexane/EtOAc 8:2, alkyne **10d** was obtained as yellow microcrystals (412 mg, 1.46 mmol, 96%).

^{1}H NMR (500 MHz, $CDCl_3$) δ_H 7.69 – 7.52 (m, 2H), 7.46 – 7.35 (m, 3H), 7.29 (d, J = 8.4 Hz, 2H), 6.85 (d, J = 9.0 Hz, 2H), 6.60 (s, 1H), 6.50 (d, J = 2.3 Hz, 1H), 3.78 (s, 3H), 2.70 (d, J = 2.3 Hz, 1H). **^{13}C NMR** (126 MHz, $CDCl_3$) δ_C 156.32, 152.55, 136.69, 130.58, 129.27, 128.87, 127.84, 120.83, 114.41, 80.48, 75.87, 66.35, 55.63. **FTIR** (ATR, solid powder) ν_{max} 3301, 3002, 2936, 2837, 2126 (weak, C≡C stretch), 1692, 1614, 1597, 1528, 1453, 1322, 1303, 1251, 1228, 1173, 1111, 1049, 1025, 994, 953, 918, 864, 828, 789, 754, 726, 695 cm^{-1}. **LRMS** (+*ve* ESI-LCMS, CH_3CN/water/CF_3COOH) *m*/*z* 279.0 ([M-2H]$^+$ 100%). **HRMS** (TOF MS ASAP +*ve*) *m*/*z* calculated for $C_{17}H_{16}NO_3$ 282.1130, found 282.1128.

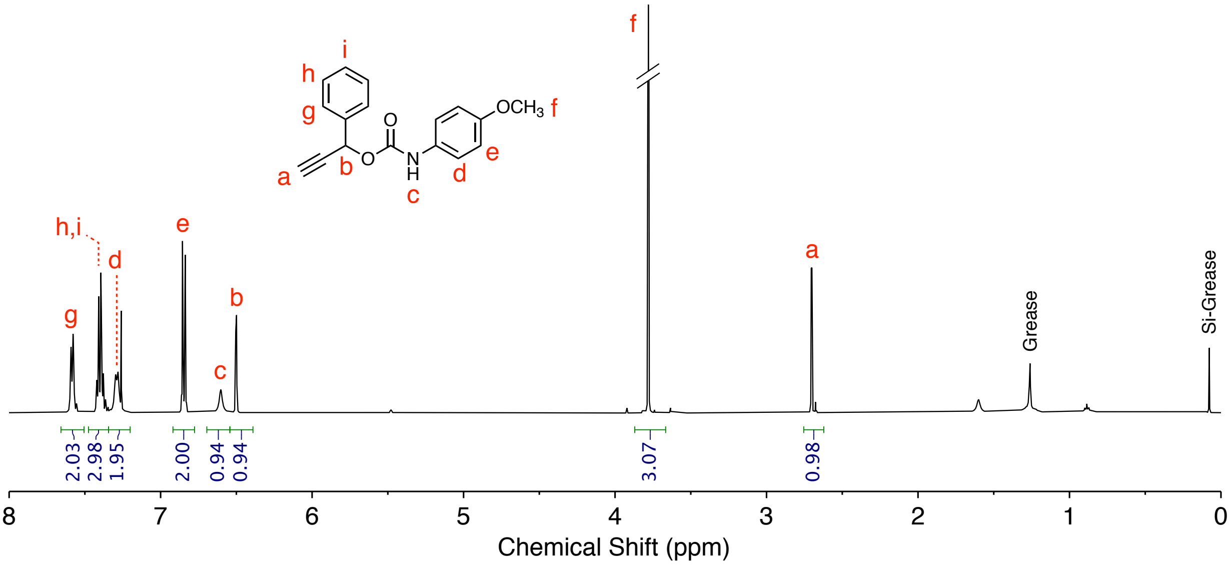

Figure S27. ^{1}H NMR spectrum (500 MHz, 298 K, $CDCl_3$) of compound **10d.**

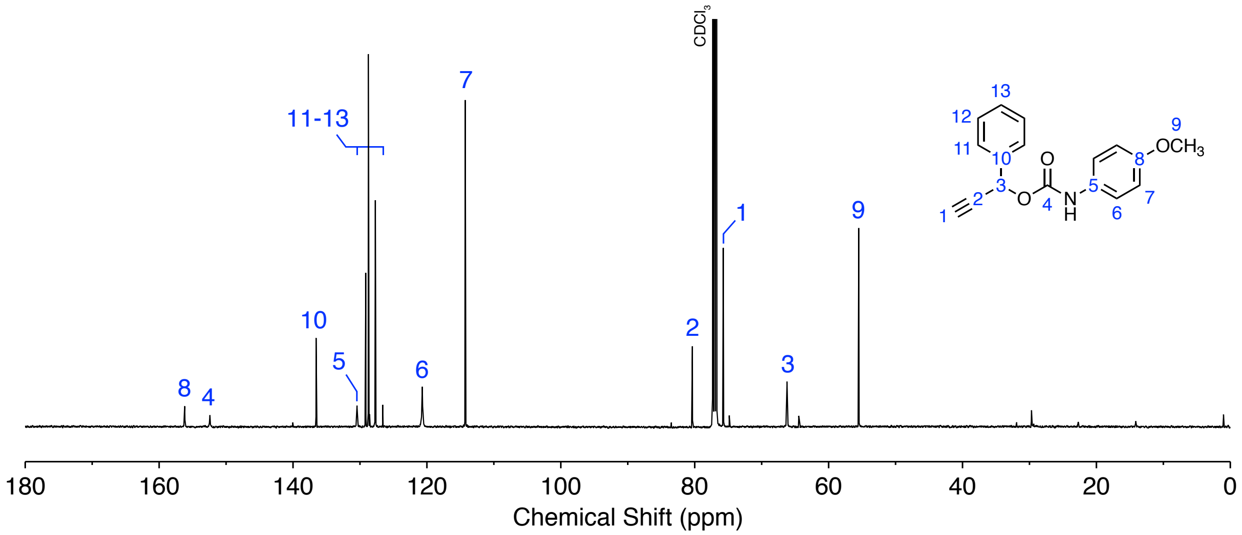

Figure S28. ^{13}C NMR spectrum (126 MHz, 298 K, $CDCl_3$) of compound **10d**.

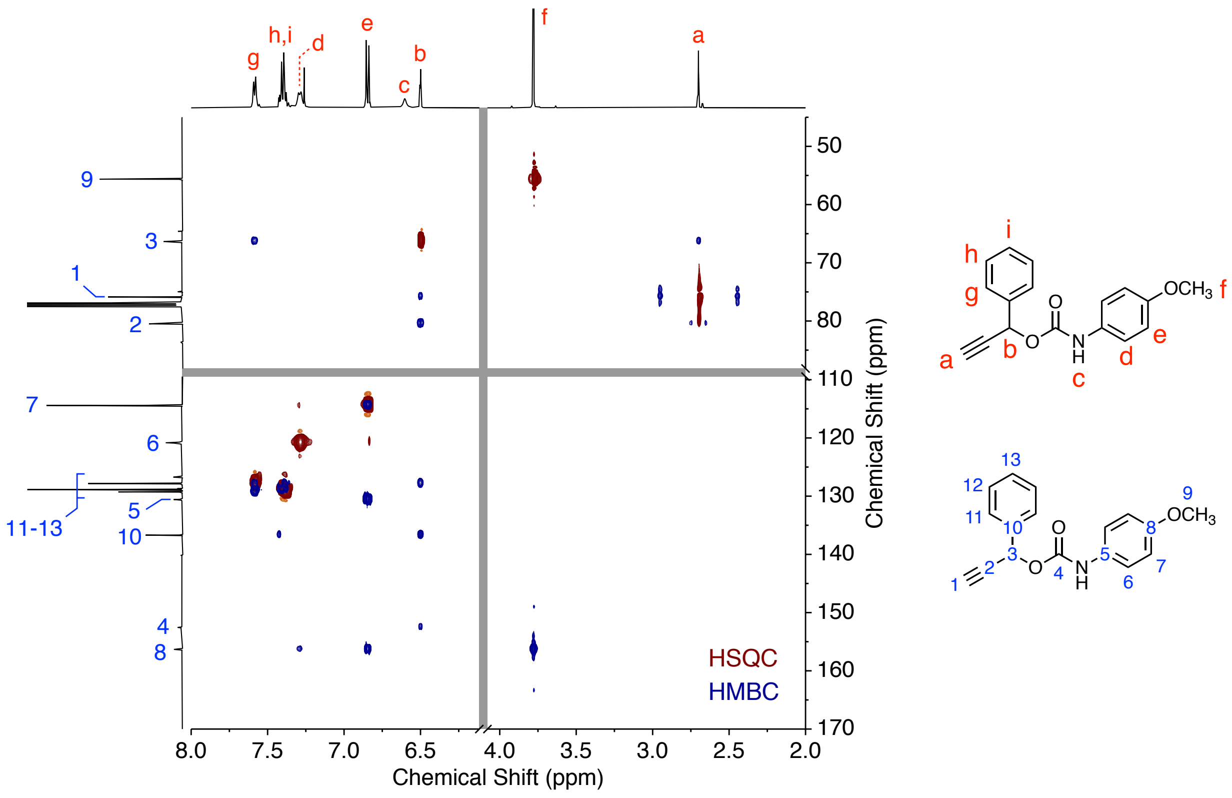

Figure S29. Overlaid ^{1}H–^{13}C NMR HSQC and HMBC spectra (500/126 MHz, 298 K, $CDCl_3$) of compound **10d**. Grey lines denote cuts in the spectrum to remove whitespace.

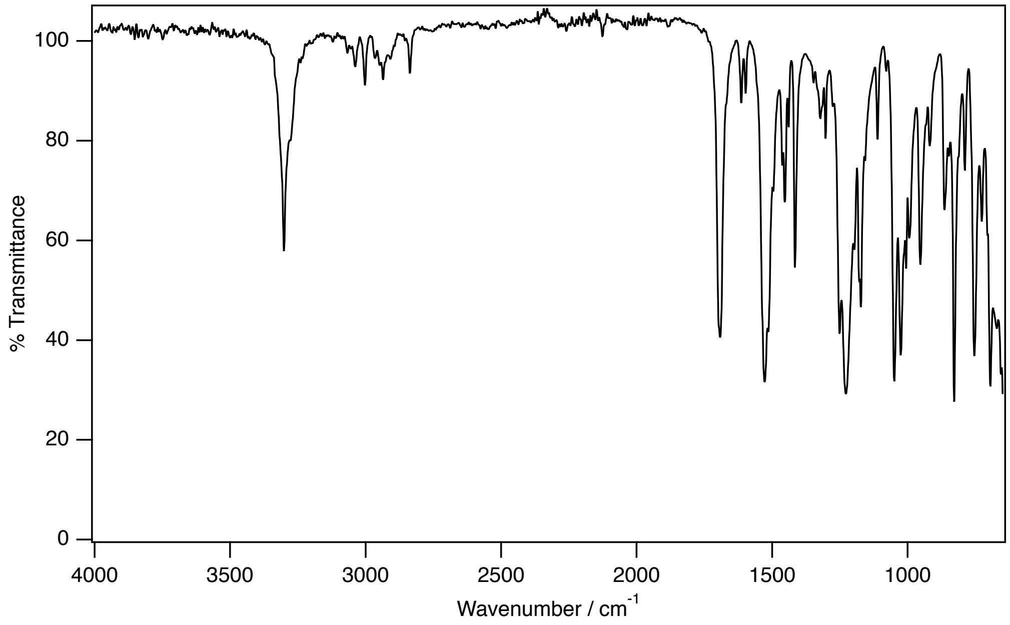

Figure S30. FTIR spectrum (ATR, solid powder) of compound **10d**. The alkyne stretching frequency (~2100 cm^{-1}) was observed to be extremely weak by this technique.

Monoisotopic Mass, Even Electron Ions
3 formula(e) evaluated with 1 results within limits (up to 50 closest results for each mass)
Elements Used:
C: 1-17 H: 1-16 B: 0-1 N: 1-1 O: 1-3
JRN_45339 B Pilgrim
JRN_45339 B Pilgrim 861 (1.874) Cm (833:958) 1: TOF MS ASAP+ 3.10e+005

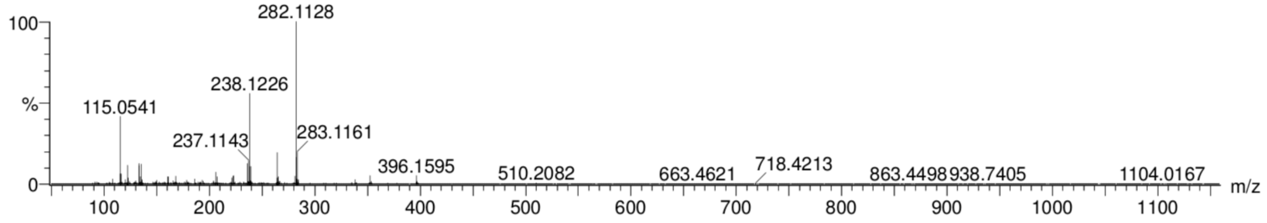

Mass	Calc. Mass	mDa	PPM	DBE	i-FIT	Norm	Conf(%)	Formula
Minimum:				-1.5				
Maximum:		5.0	5.0	50.0				
282.1128	282.1130	-0.2	-0.7	10.5	1888.3	n/a	n/a	C17 H16 N O3

Figure S31. HRMS spectrum (TOF MS ASAP +*ve*) and analysis report for compound **10d**.

S1.1.7. Characterization data for Alkyne 10e

After purification by silica gel chromatography in hexane/EtOAc 7:3 to 1:1, alkyne **10e** was obtained as a yellow oil that crystallised into pale-yellow needles upon standing (1.01 g, 3.37 mmol, 99%). **^{1}H NMR** (500 MHz, $CDCl_3$) δ_H 7.42 – 7.22 (m, 5H), 7.12 – 7.02 (m, 1H), 6.85 (d, J = 9.0 Hz, 2H), 6.64 (s, 1H), 6.48 (d, J = 2.3 Hz, 1H), 3.78 (s, 3H), 2.71 (d, J = 2.3 Hz, 1H). **^{13}C NMR** (126 MHz, $CDCl_3$) δ_C 162.92 (d, $^{1}J_{C\text{-}F}$ = 247.0 Hz), 156.41, 152.37, 139.08 (d, $^{3}J_{C\text{-}F}$ = 7.3 Hz), 130.43 (d, $^{3}J_{C\text{-}F}$ = 8.2 Hz), 130.40, 123.40 (d, $^{4}J_{C\text{-}F}$ = 3.0 Hz), 120.92, 116.20 (d, $^{2}J_{C\text{-}F}$ = 21.1 Hz), 114.81 (d, $^{2}J_{C\text{-}F}$ = 22.8 Hz), 114.44, 79.93, 76.19, 65.53, 55.63. **^{19}F NMR** (376 MHz, $CDCl_3$) δ_F −112.13. **FTIR** (ATR, solid powder) ν_{max} 3293, 3068, 2935, 2839, 2125 (weak, C≡C stretch), 1705, 1595, 1512, 1487, 1451, 1414, 1297, 1267, 1205, 1177, 1140, 1113, 1028, 963, 867, 856, 827, 773, 687 cm^{-1}. **LRMS** (+*ve* ESI-LCMS, CH_3CN/water/CF_3COOH) *m/z* 621.2 ($[2M+Na]^+$ 30%), 300.0 ($[M+H]^+$ 100), 151.1 ($[M-C_9H_7O]^+$ 55). **HRMS** (TOF MS ASAP +*ve*) *m/z* calculated for $C_{17}H_{15}NO_3F$ 300.1036, found 300.1035.

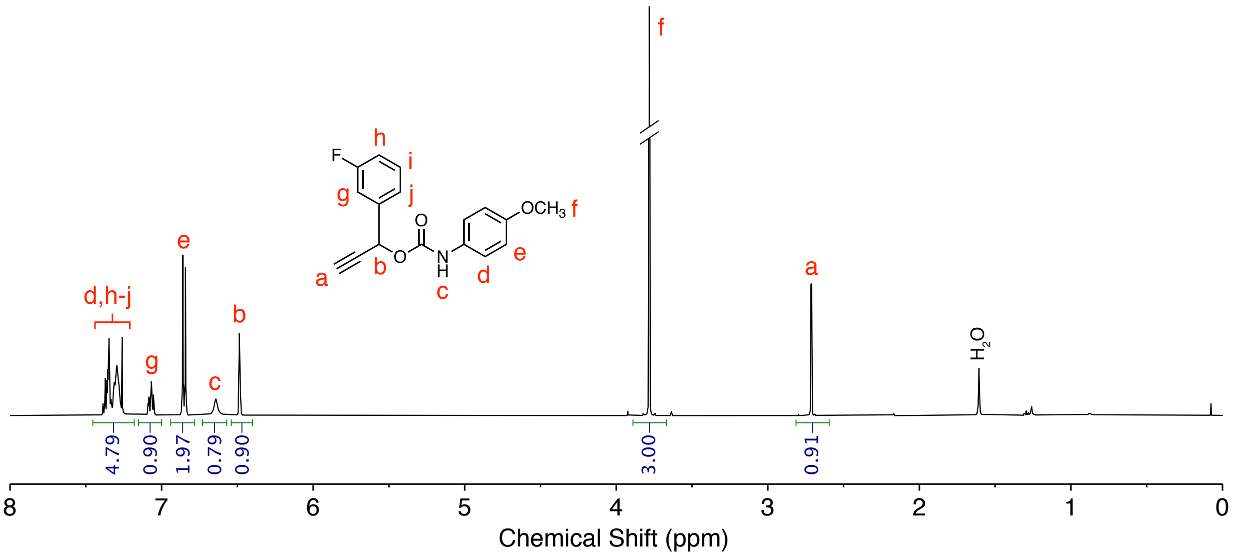

Figure S32. ^{1}H NMR spectrum (500 MHz, 298 K, $CDCl_3$) of compound **10e.**

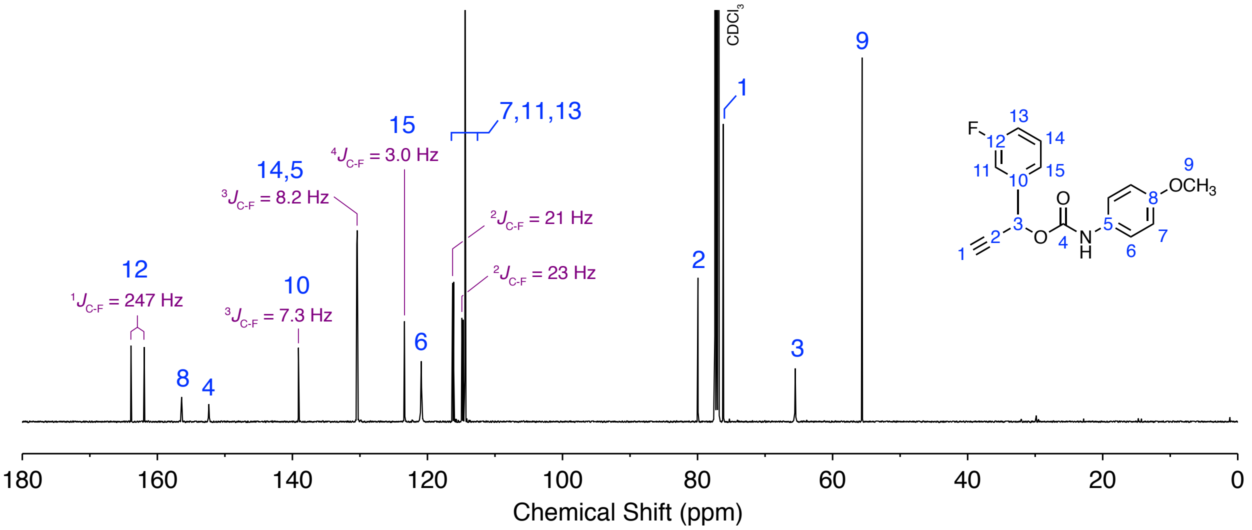

Figure S33. ^{13}C NMR spectrum (126 MHz, 298 K, $CDCl_3$) of compound **10e**, with ^{19}F–^{13}C couplings highlighted.

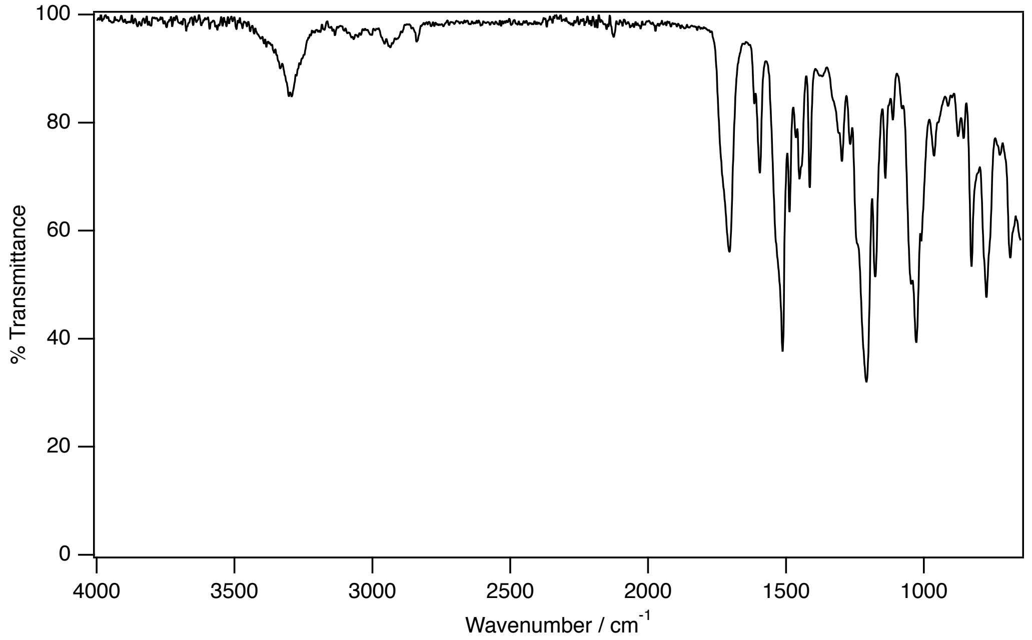

Figure S34. FTIR spectrum (ATR, solid powder) of compound **10e**. The alkyne stretching frequency (~2100 cm^{-1}) was observed to be extremely weak by this technique.

Monoisotopic Mass, Even Electron Ions
6 formula(e) evaluated with 1 results within limits (up to 50 closest results for each mass)
Elements Used:
C: 1-17 H: 1-15 B: 0-1 N: 1-1 O: 1-3 F: 0-1
JRN_45338 B Pilgrim
JRN_45338 B Pilgrim 861 (1.874) Cm (775:863)

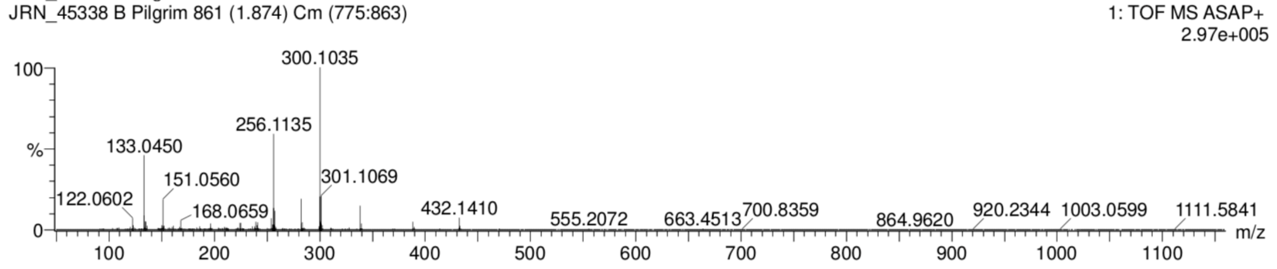

Minimum:				-1.5				
Maximum:		5.0	5.0	50.0				
Mass	Calc. Mass	mDa	PPM	DBE	i-FIT	Norm	Conf(%)	Formula
300.1035	300.1036	-0.1	-0.3	10.5	1602.4	n/a	n/a	C17 H15 N O3 F

Figure S35. HRMS spectrum (TOF MS ASAP +*ve*) and analysis report for compound **10e**.

S1.1.8. Characterization data for Alkyne 10f

After purification by silica gel chromatography in hexane/EtOAc 8:2 to 7:3, alkyne **10f** was obtained as yellow-brown needles (838 mg, 2.69 mmol, 42%).

^{1}H NMR (500 MHz, $CDCl_3$) δ_H 7.40 – 7.24 (m, 3H), 7.16 (d, J = 7.9 Hz, 1H), 7.13 (d, J = 2.3 Hz, 1H), 6.92 (ddd, J = 8.3, 2.6, 1.0 Hz, 1H), 6.85 (d, J = 9.0 Hz, 2H), 6.63 (s, 1H), 6.47 (d, J = 2.3 Hz, 1H), 3.83 (s, 3H), 3.78 (s, 3H), 2.70 (d, J = 2.3 Hz, 1H). **^{13}C NMR** (126 MHz, $CDCl_3$) δ_H 159.80, 156.18, 152.38, 137.99, 130.43, 129.80, 120.71, 119.90, 114.73, 114.28, 113.14, 80.29, 75.70, 66.07, 55.50, 55.33. **FTIR** (ATR, solid powder) ν_{max} 3300, 2937, 2836, 2332, 2253, 2124 (weak, C≡C stretch), 1708, 1600, 1513, 1489, 1464, 1413, 1312, 1284, 1244, 1204, 1177, 1112, 1027, 958, 907, 852, 827, 770, 728, 692 cm^{-1}. **LRMS** (+*ve* ESI-LCMS, CH_3CN/water/CF_3COOH) *m*/*z* 645.2 ($[2M+Na]^+$ 30), 334.1 ($[M+Na]^+$ 10), 312.1 ($[M+H]^+$ 100). **HRMS** (TOF MS ASAP +*ve*) *m*/*z* calculated for $C_{18}H_{18}NO_4$ 312.1236, found 312.1239.

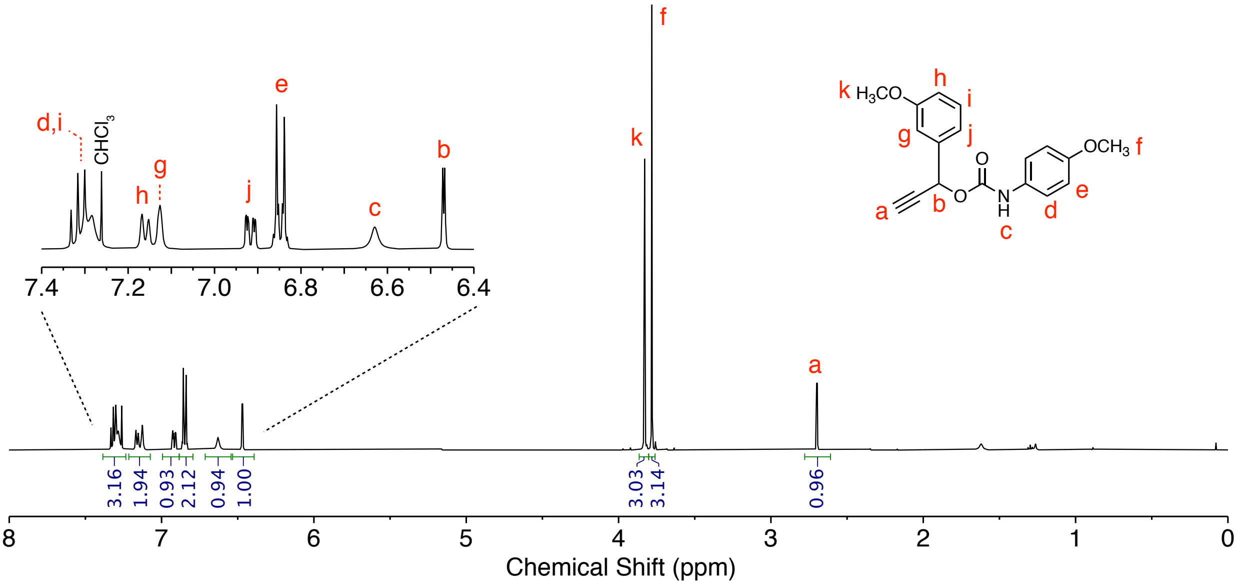

Figure S36. ^{1}H NMR spectrum (500 MHz, 298 K, $CDCl_3$) of compound **10f.**

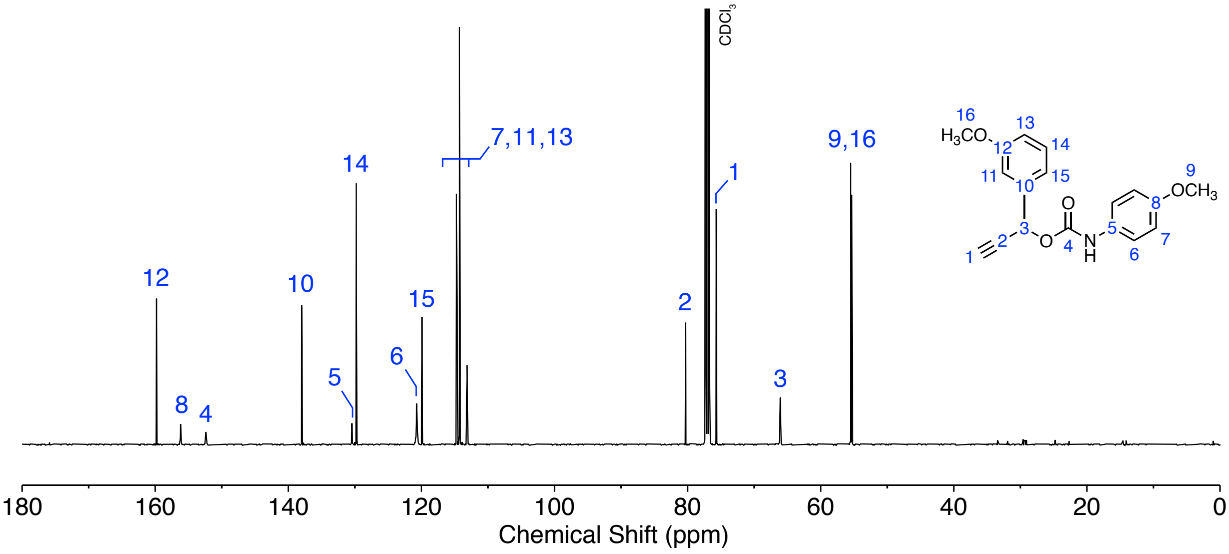

Figure S37. ^{13}C NMR spectrum (126 MHz, 298 K, $CDCl_3$) of compound **10f**, with ^{19}F–^{13}C couplings highlighted.

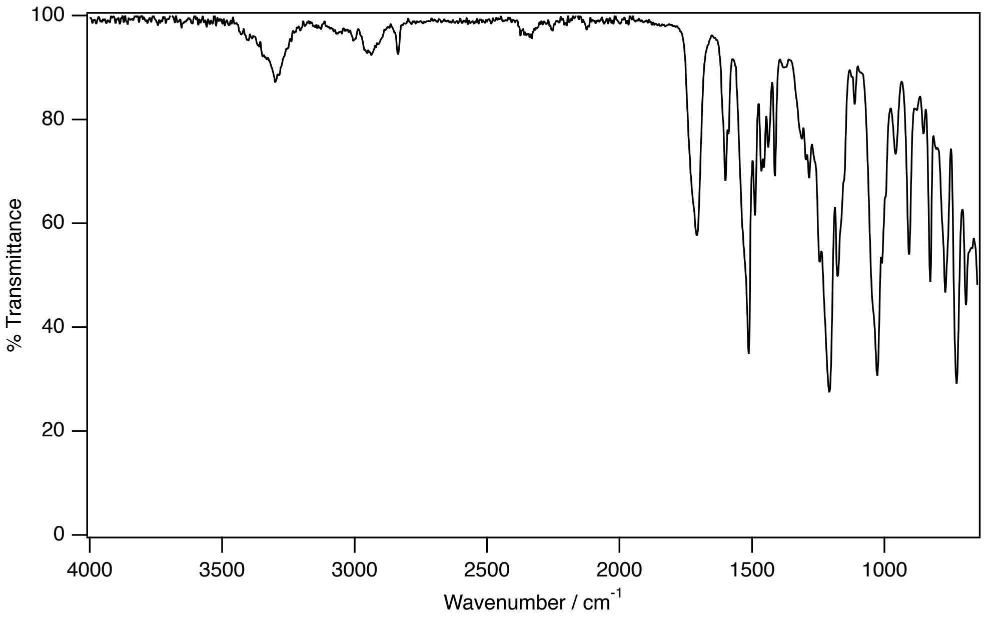

Figure S38. FTIR spectrum (ATR, solid powder) of compound **10f**. The alkyne stretching frequency (~2100 cm^{-1}) was observed to be extremely weak by this technique.

Monoisotopic Mass, Even Electron Ions
5 formula(e) evaluated with 1 results within limits (up to 50 closest results for each mass)
Elements Used:
C: 1-18 H: 1-18 B: 0-1 N: 1-1 O: 1-4
JRN_45337 B Pilgrim
JRN_45337 B Pilgrim 1377 (2.975) Cm (1377:1488)

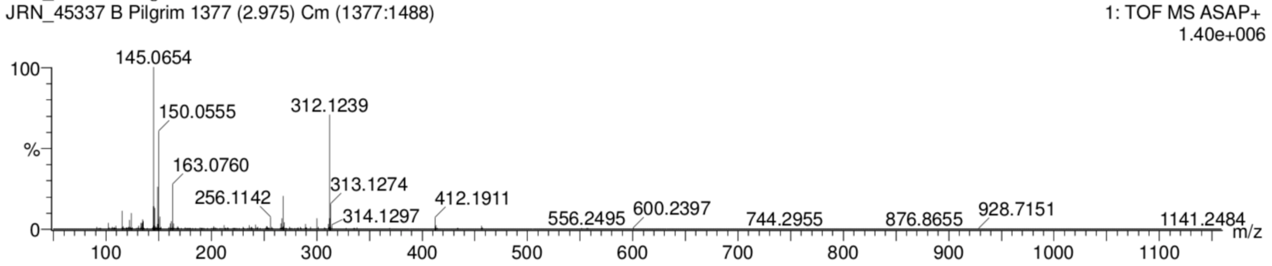

Minimum:				-1.5				
Maximum:		5.0	5.0	50.0				
Mass	Calc. Mass	mDa	PPM	DBE	i-FIT	Norm	Conf(%)	Formula
312.1239	312.1236	0.3	1.0	10.5	2662.0	n/a	n/a	C18 H18 N O4

Figure S39. HRMS spectrum (TOF MS ASAP +*ve*) and analysis report for compound **10f**.

S5. Synthesis of Self-immolative Model Compounds (1a–f)

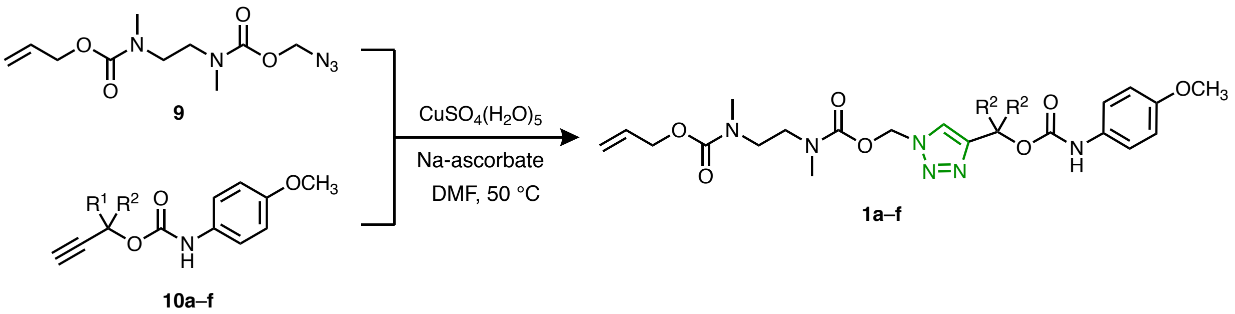

Scheme S2. General scheme for the synthesis of self-immolative model compounds **1a–f**.

Table S1. Summary of CuAAC coupling yields (isolated gravimetric yield) for the formation of model compounds **1a–f**.

Congener	R^1	R^2	Yield (%)
**1a**	H	H	80
**1b**	H	Me	87
**1c**	Me	Me	56
**1d**	H	Ph	64
**1e**	H	3-fluorophenyl	40
**1f**	H	3-methoxylphenyl	89

General Procedure: A vial was charged with azide **9** (156 mg, 575 mmol, 1 equiv.), alkyne **10a-f** (559 mmol, 0.97 equiv.), copper(II) sulfate pentahydrate (56 mmol, 0.1 equiv.), sodium ascorbate (275 mmol, 0.48 equiv.) and DMF (2 mL). The reaction mixture, typically a brown solution or suspension, was stirred at 50 °C for up to 4 h, monitoring progress by TLC (typically Hex/EtOAc = 2:8, product R_f 0.3–0.60). Upon complete consumption of the alkyne, the reaction mixture was diluted with EtOAc (30 mL) and washed with an aqueous solution of sodium EDTA (2.5% w/v, 3 × 20 mL), water (2 × 20 mL), brine (20 mL), dried over Na_2SO_4, filtered and the solvents removed by rotary evaporation. The resulting crude product was purified by silica chromatography using hexane/EtOAc = 3:7 to 1:9 as eluent.

S5.1. Characterization data for Model 1a

The product was obtained as a pale yellow, highly viscous oil (219 mg, 460 µmol, 80%). **^{1}H NMR** (500 MHz, DMSO-*d*6) δ_H 9.54 (s, 1H), 8.28 – 8.13 (m, 1H), 7.34 (d, *J* = 8.4 Hz, 2H), 6.85 (d, *J* = 9.0 Hz, 2H), 6.29 – 6.14 (m, 2H), 5.98 – 5.79 (m, 1H), 5.38 – 5.04 (m, 4H), 4.54 – 4.32 (m, 2H), 3.70 (s, 3H), 3.35 (s, 4H; overlapping with water peak), 2.96 – 2.62 (m, 6H). **^{13}C NMR** (126 MHz, DMSO-*d*6) δ_C 155.40, 155.15, 154.88, 154.20, 153.94, 153.28, 142.81, 134.01 – 132.92 (m), 131.98, 126.18 – 125.63 (m), 119.82, 117.36 – 116.99 (m), 116.69 (d, *J* = 17.8 Hz), 70.67, 66.36 – 64.34 (m), 56.97, 55.18, 47.27 – 44.75 (m), 35.30 – 32.68 (m). **FTIR** (ATR, solid powder) ν_{max} 3333, 2937, 2356, 1717, 1679, 1601, 1541, 1514, 1462, 1404, 1297, 1210, 1172, 1119, 1044, 1031, 993, 908, 822, 794, 760 cm^{-1}. **LRMS** (+*ve* ESI-LCMS, CH_3CN/water/CF_3COOH) *m*/*z*. 499.1 ([M+Na]$^+$ 25%), 477.1 ([M+H]$^+$ 100). **HRMS** (TOF MS ASAP +*ve*) *m*/*z* calculated for $C_{21}H_{29}N_6O_7$ 477.2098, found 477.2098.

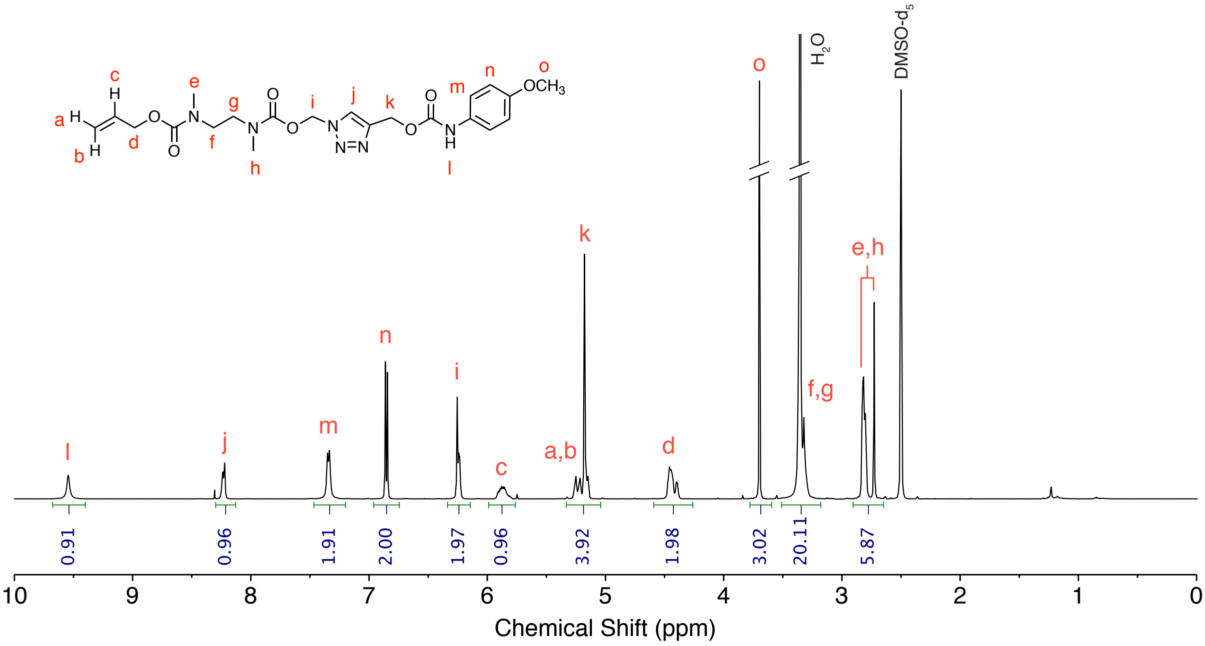

Figure S40. ^{1}H NMR (500 MHz, DMSO, 298 K) spectrum of compound **1a**

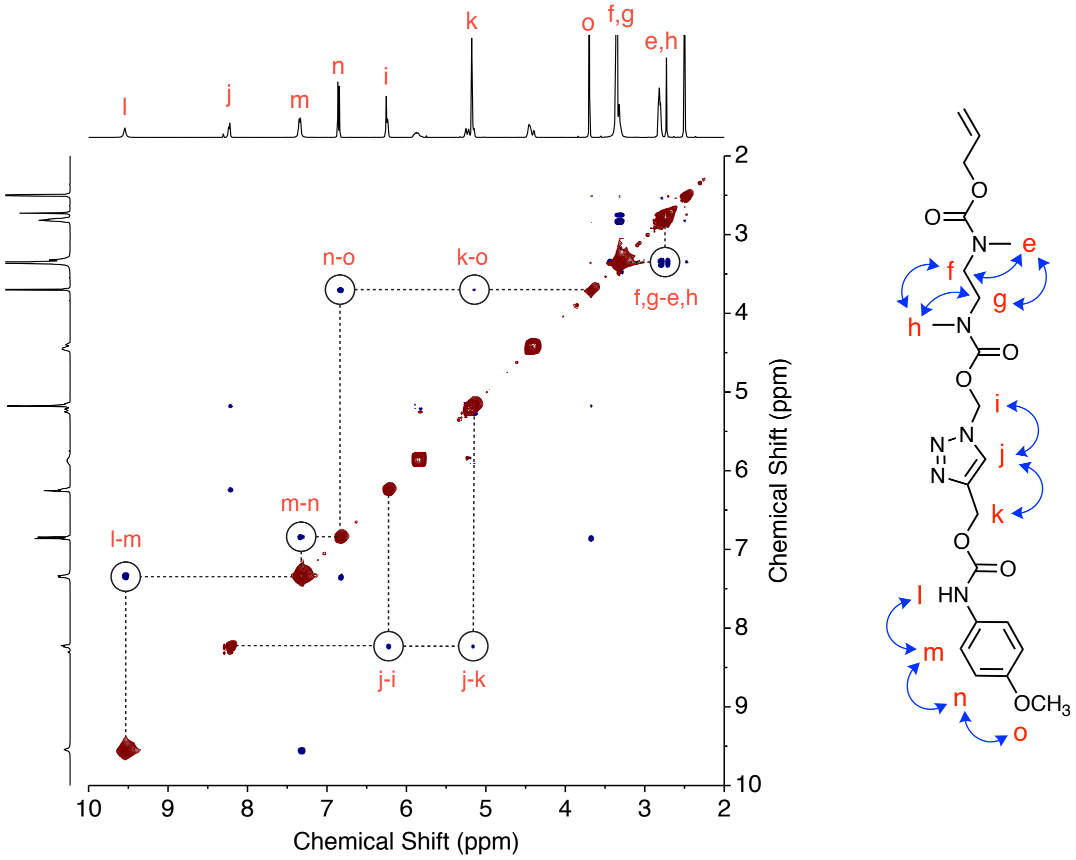

Figure S41. ^{1}H-^{1}H NOESY NMR (400 MHz, DMSO-d_6, 294 K; T_{mix} = 500 ms) spectrum of compound **1a**. ^{1}H assignment was achieved using the highlighted NOEs. (Baseline: Whittaker smoother; window functions: [f_1] sine square 90°, sine bell II 0%, first point 0.5, [f_2] sine square 90°; COSY-like symmetrization applied to reduce noise, validated by visual inspection).

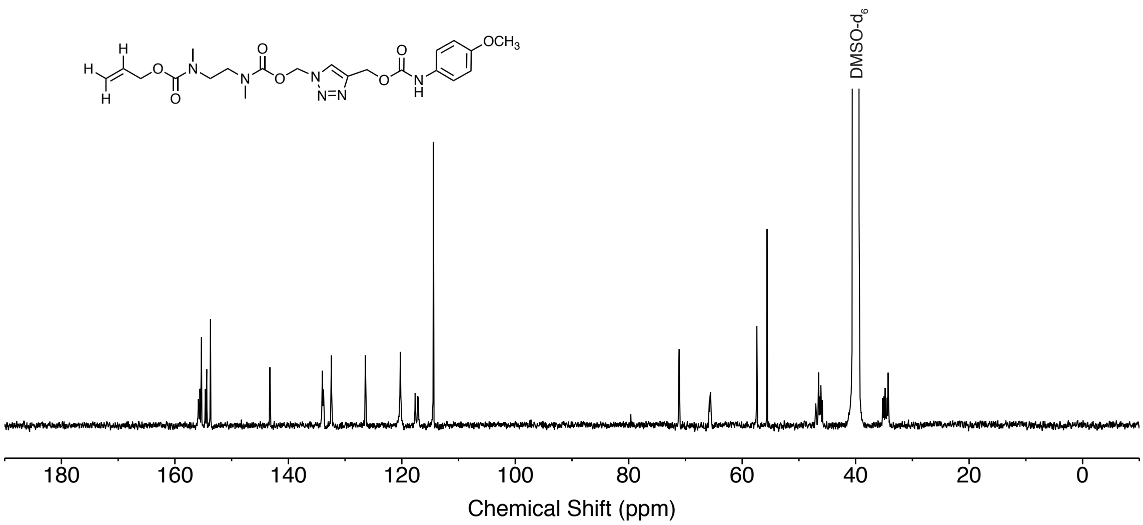

Figure S42. ^{13}C NMR (126 MHz, DMSO, 298 K) spectrum of compound **1a**. Complex splitting (e.g., peaks at ~35 and 45 ppm) is attributed to rotamers due to slow rotation around the urethane C–N bonds.

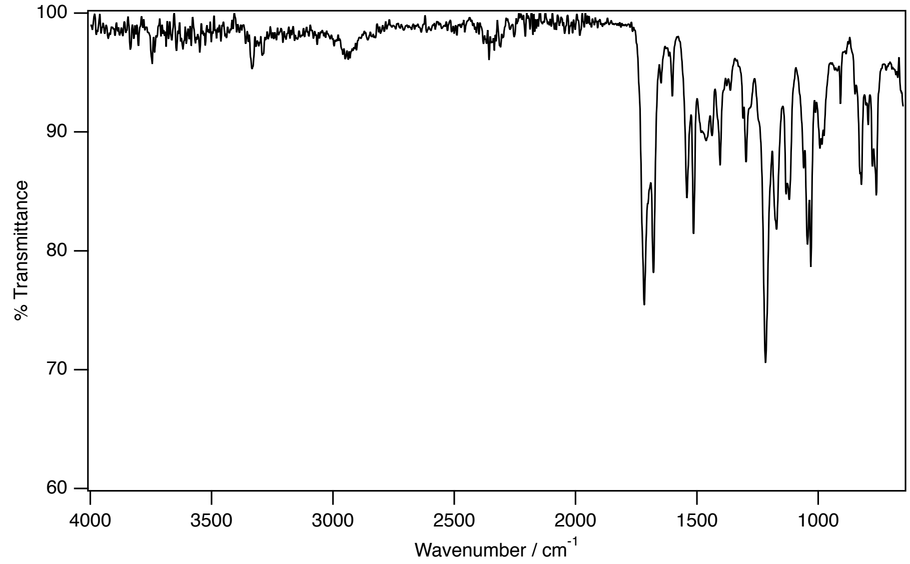

Figure S43. FTIR spectrum (ATR, solid powder) of compound **1a**.

Monoisotopic Mass, Even Electron Ions
26 formula(e) evaluated with 1 results within limits (all results (up to 1000) for each mass)
Elements Used:
C: 0-21 H: 1-29 N: 1-6 O: 3-7
JRN_45370 B Pilgrim 1721 (3.709)

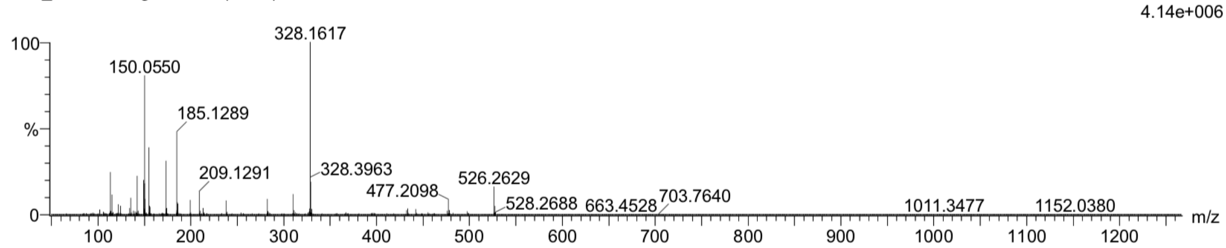

Minimum:				-1.5				
Maximum:		5.0	5.0	50.0				
Mass	Calc. Mass	mDa	PPM	DBE	i-FIT	Norm	Conf(%)	Formula
477.2098	477.2098	0.0	0.0	10.5	887.5	n/a	n/a	C21 H29 N6 O7

Figure S44. HRMS spectrum (TOF MS ASAP +*ve*) and analysis report for compound **1a**.

S5.2. Characterization data for Model 1b

The product was obtained as a colorless, highly viscous oil (420 mg, 856 µmol, 87%). **^{1}H NMR** (500 MHz, DMSO-d_6) δ_H 9.52 (s, 1H), 8.35 – 8.03 (m, 1H), 7.35 (d, J = 8.3 Hz, 2H), 6.94 – 6.71 (m, 2H), 6.25 (s, 2H), 5.95 (q, J = 6.6 Hz, 1H), 5.92 – 5.78 (m, 1H), 5.33 – 5.10 (m, 2H), 4.60 – 4.29 (m, 2H), 3.70 (s, 3H), 3.35 (d, J = 16.7 Hz, 4H), 2.94 – 2.63 (m, 6H), 1.63 (d, J = 6.8 Hz, 3H). **^{13}C NMR** (126 MHz, DMSO-d_6) δ_C 155.56 – 155.25 (m), 155.11, 154.78, 154.19, 153.92, 152.88, 147.50, 134.06 – 132.96 (m), 132.07, 125.07 – 122.72 (m), 119.71, 116.91 (m), 113.92, 70.67, 65.69 – 64.81 (m), 64.36, 55.14, 47.33 – 44.87 (m), 36.09 – 33.00 (m), 19.74. **FTIR** (ATR, solid powder) ν_{max} 3306, 2936, 1697, 1601, 1513, 1457, 1409, 1296, 1210, 1172, 1147, 1118, 1071, 1026, 971, 930, 829, 767 cm^{-1}. **HRMS** (TOF MS ASAP +*ve*) *m/z* calculated for $C_{22}H_{31}N_6O_7$ 491.2254, found 491.2237.

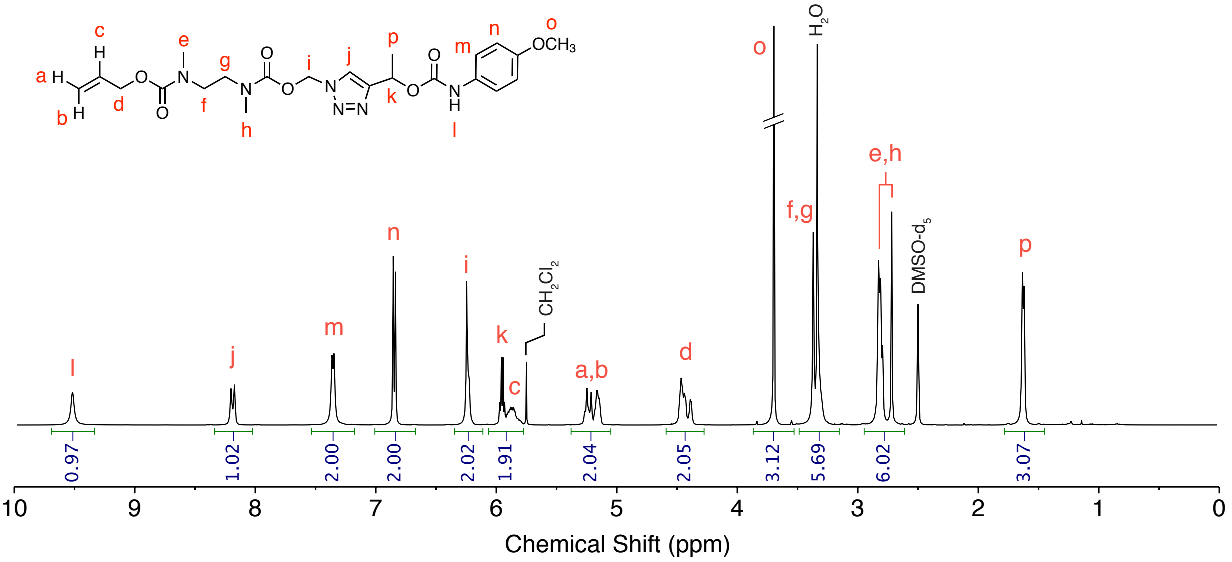

Figure S45. ^{1}H NMR (500 MHz, DMSO-d_6, 298 K) spectrum of compound **1b**.

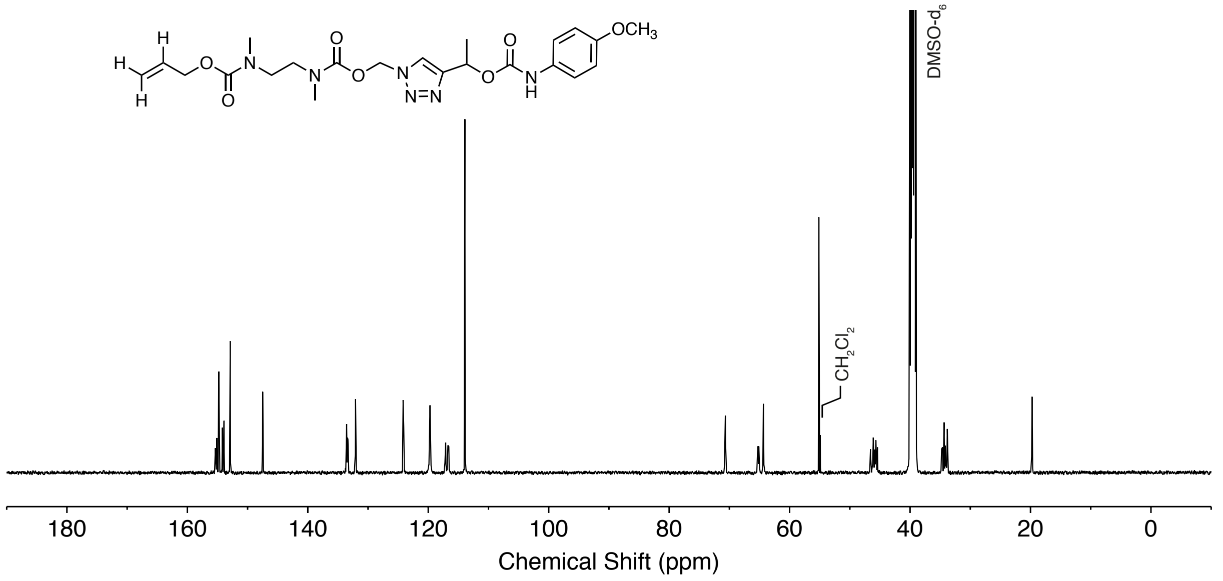

Figure S46. ^{13}C NMR (126 MHz, DMSO-d_6, 298 K) spectrum of compound **1b**.

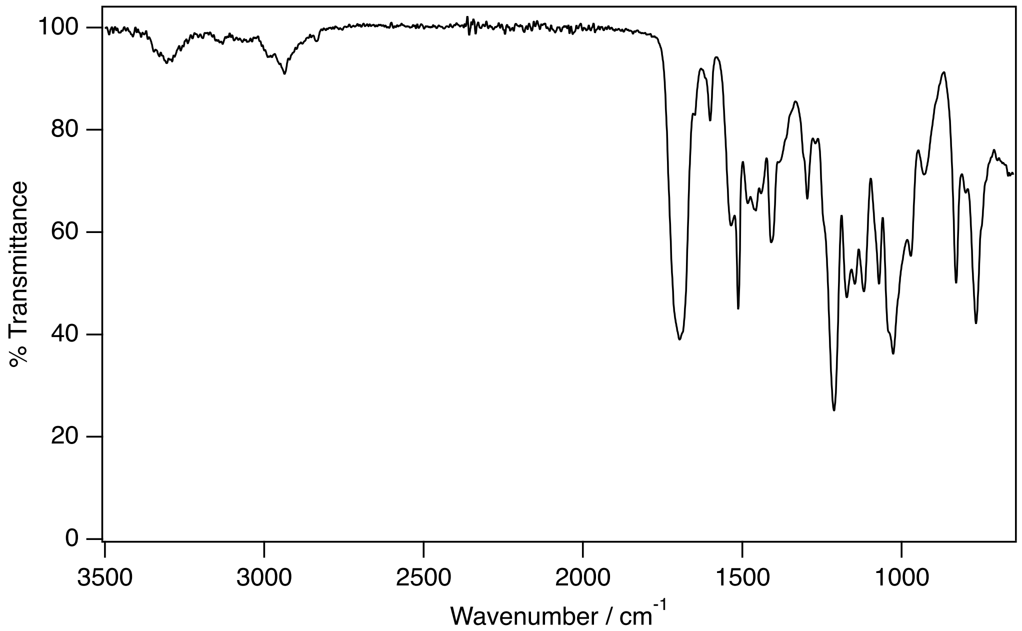

Figure S47. FTIR spectrum (ATR, solid powder) of compound **1b.**

Monoisotopic Mass, Even Electron Ions
26 formula(e) evaluated with 1 results within limits (all results (up to 1000) for each mass)
Elements Used:
C: 0-22   H: 1-31   N: 1-6   O: 3-7
JRN_45369 B Pilgrim 1205 (2.608)   1: TOF MS ASAP+ 1.22e+004

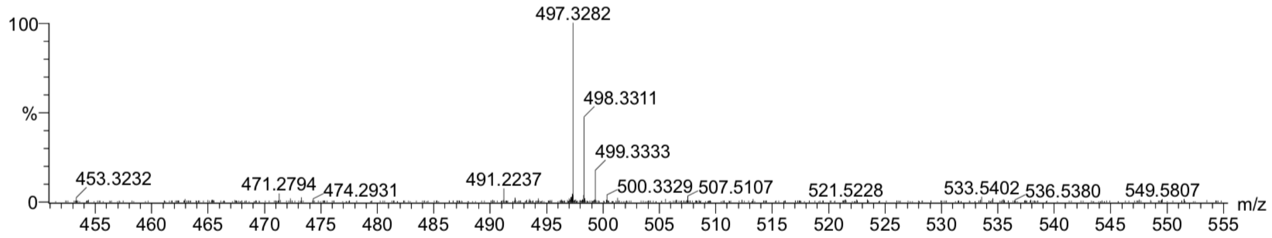

Minimum:				-1.5				
Maximum:		5.0	5.0	50.0				
Mass	Calc. Mass	mDa	PPM	DBE	i-FIT	Norm	Conf(%)	Formula
491.2237	491.2254	-1.7	-3.5	10.5	137.3	n/a	n/a	C22 H31 N6 O7

Figure S48. HRMS spectrum (TOF MS ASAP +ve) and analysis report for compound **1b**.

S5.3. Characterization data for Model 1c

The product was obtained as a colorless, highly viscous oil (164 mg, 325 µmol, 56%). **^{1}H NMR** (400 MHz, DMSO-*d*$_6$) δ_H 9.33 (s, 1H), 8.21 – 8.04 (m, 1H), 7.28 (d, *J* = 8.9 Hz, 2H), 6.90 – 6.70 (m, 2H), 6.22 (m, 2H), 5.87 (m, 1H), 5.35 – 5.05 (m, 2H), 4.58 – 4.33 (m, 2H), 3.68 (s, 3H), 3.34 (s, 4H), 2.91 – 2.62 (m, 6H), 1.81 (br s, 6H). **^{13}C NMR** (126 MHz, DMSO-*d*$_6$) δ_C 155.39, 155.15, 154.61, 154.24, 153.92, 152.22, 151.65, 134.11 – 132.87 (m), 132.28, 122.91 (d, *J* = 12.1 Hz), 119.60, 116.96 (dd, *J* = 65.0, 16.4 Hz), 113.84, 75.54, 70.75, 65.20 (d, *J* = 17.3 Hz), 55.16, 47.40 – 44.82 (m), 34.30 (dd, *J* = 72.1, 39.0 Hz), 27.55. **FTIR** (ATR, solid powder) ν_{max} 2946, 1700, 1603, 1513, 1467, 1410, 1297, 1226, 1138, 1024, 832, 767 cm^{-1}. **LRMS** (+*ve* ESI-LCMS, CH_3CN/water/CF_3COOH) *m*/*z* 527.2 ($[M+Na]^+$ 100%). **HRMS** (TOF MS ASAP +*ve*) *m*/*z* calculated for $C_{23}H_{32}N_6O_7Na$ 527.2230, found 527.2228.

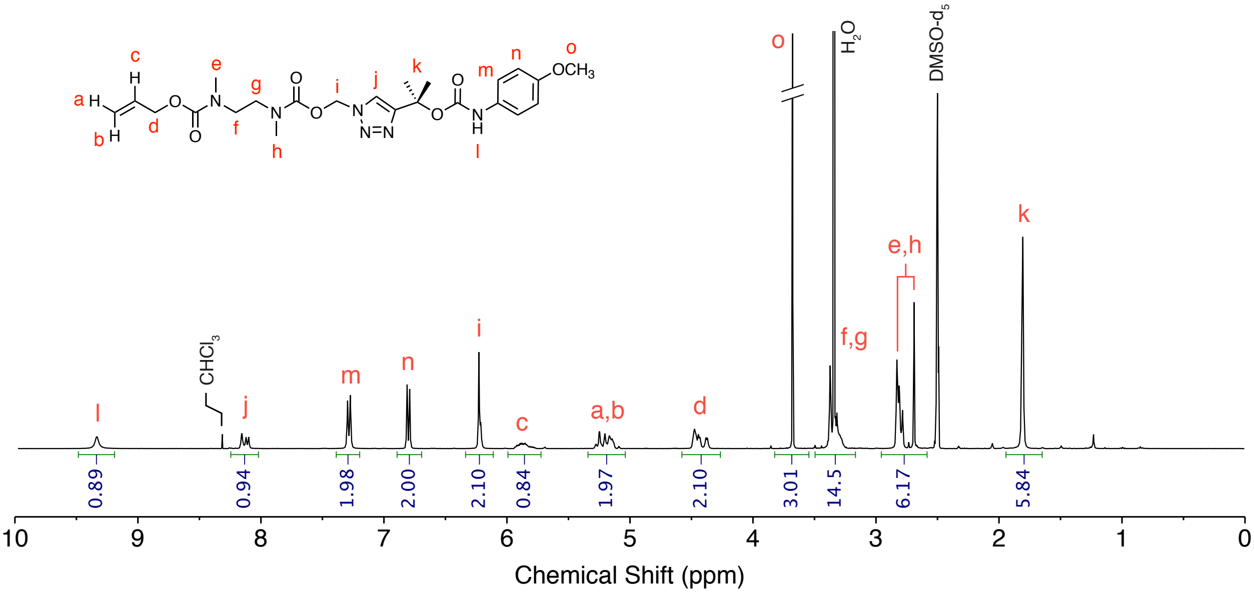

Figure S49. ^{1}H NMR spectrum (400 MHz, DMSO-*d*$_6$, 295 K) of compound **1c**.

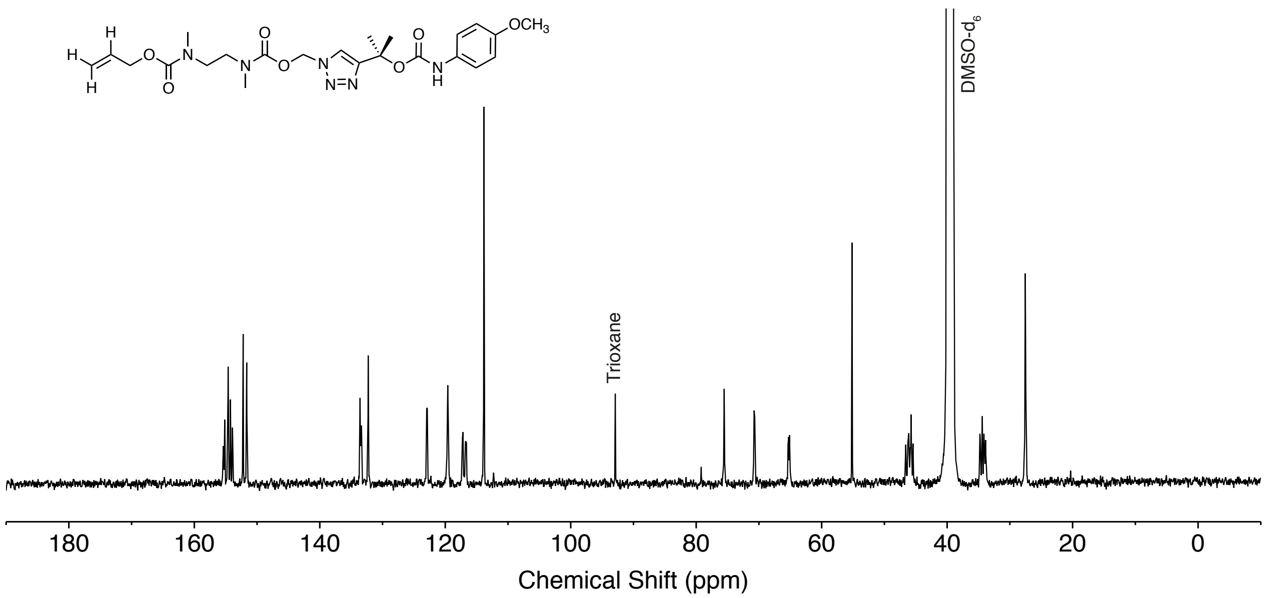

Figure S50. ^{13}C NMR spectrum (126 MHz, DMSO-*d*$_6$, 298 K) of **1c**. Sample contains trioxane standard.

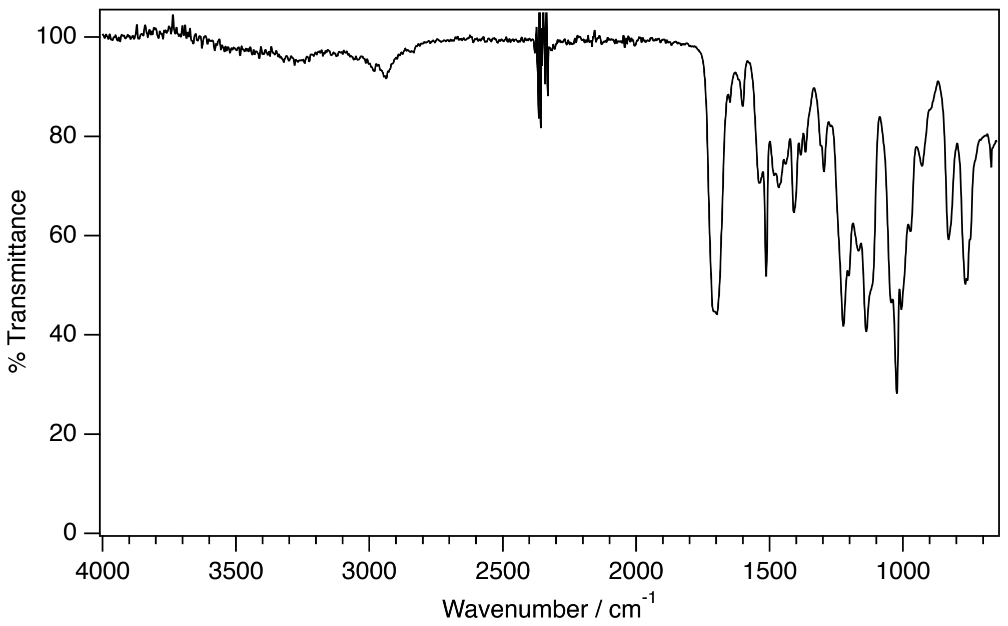

Figure S51. FTIR spectrum (ATR, solid powder) of compound **1c.**

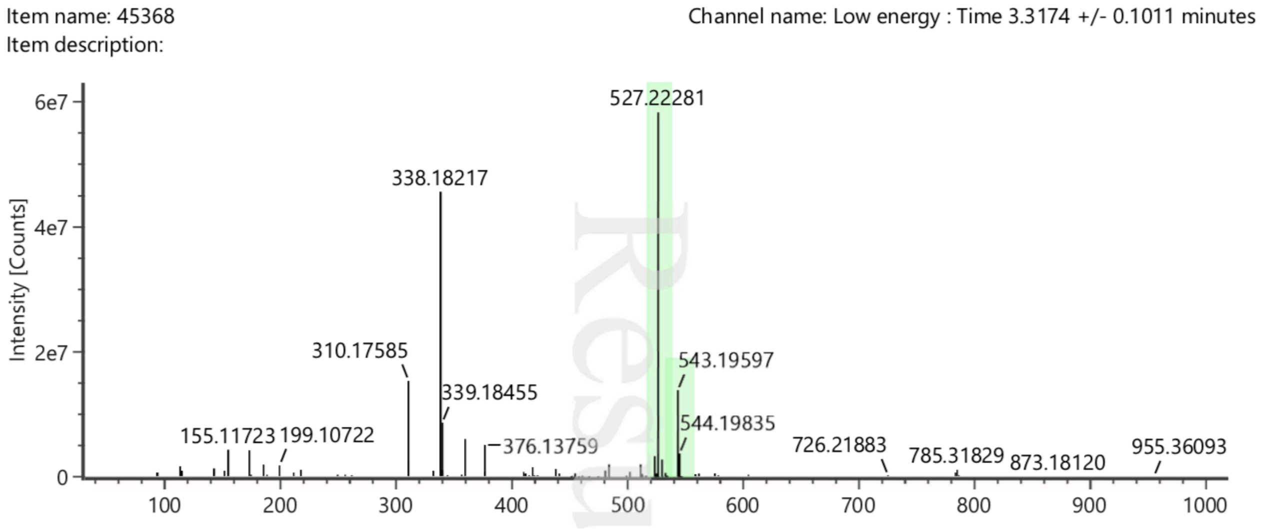

Figure S52. HRMS spectrum (TOF MS ASAP +ve) and analysis report for compound **1c**. Highlighted region contains the peak corresponding to the $[M+Na]^+$ species.

S5.4. Characterization data for Model 1d

The product was obtained as a yellow, low-melting solid (199 mg, 360 µmol, 64%). **^{1}H NMR** (400 MHz, DMSO-d_6) δ_H 9.75 (s, 1H), 8.21 – 8.04 (m, 1H), 7.54 – 7.29 (m, 7H), 6.92 (s, 1H), 6.88 – 6.80 (m, 2H), 6.29 – 6.16 (m, 2H), 5.98 – 5.74 (m, 1H), 5.29 – 5.09 (m, 2H), 4.52 – 4.33 (m, 2H), 3.69 (s, 3H), 3.34 (s, 4H), 2.91 – 2.62 (m, 6H). **^{13}C NMR** (101 MHz, $CDCl_3$) δ_C 156.66 – 155.56 (m), 155.24, 155.06, 154.64, 152.80, 148.81, 148.13, 147.88, 139.01, 138.48, 133.82 – 132.64 (m), 131.53, 130.78, 129.39 – 128.09 (m), 127.77 – 126.59 (m), 124.68 – 123.30 (m), 120.63, 120.23, 118.20, 118.02, 117.54, 117.36, 114.33, 114.24, 71.63 – 70.51 (m), 66.93 – 65.57 (m), 55.60, 49.40 – 43.32 (m), 36.41 – 32.81 (m). **FTIR** (ATR, solid powder) ν_{max} 2987, 1714, 1604, 1543, 1513, 1456, 1411, 1297, 1217, 1175, 1125, 1047, 1027, 831, 759, 699 cm^{-1}. **LRMS** (+*ve* ESI-LCMS, CH_3CN/water/CF_3COOH) *m*/*z* 575.2 ([M+Na]$^+$ 100%). **HRMS** (TOF MS ASAP +*ve*) *m*/*z* calculated for $C_{27}H_{33}N_6O_7$ 553.2411, found 553.2426.

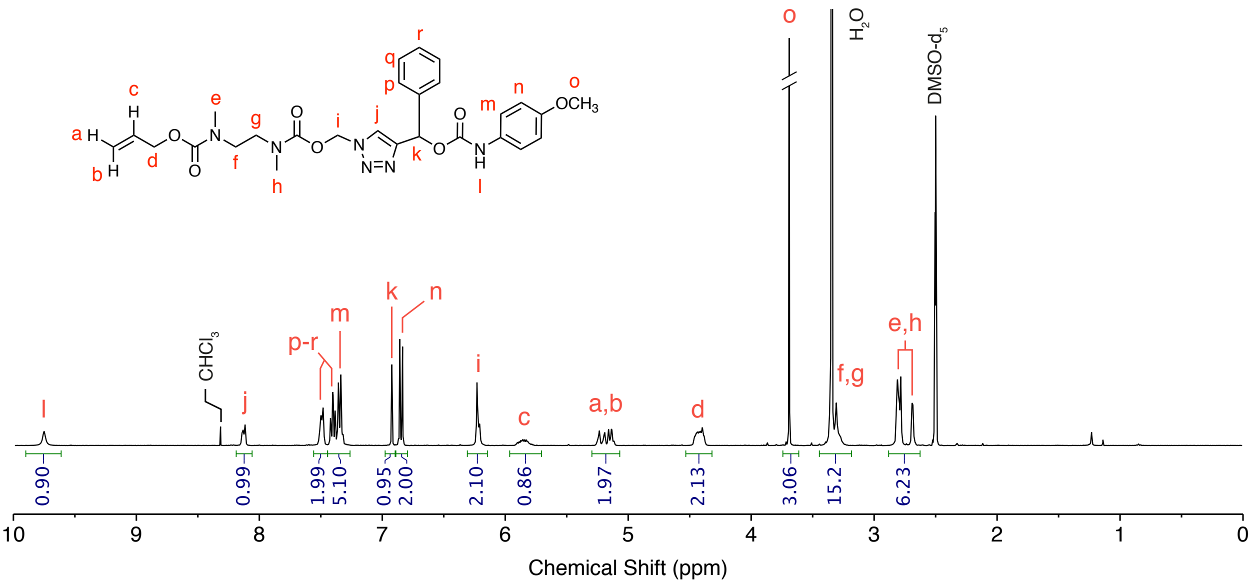

Figure S53. ^{1}H NMR spectrum (400 MHz, DMSO-d_6, 295 K) of compound **1d**.

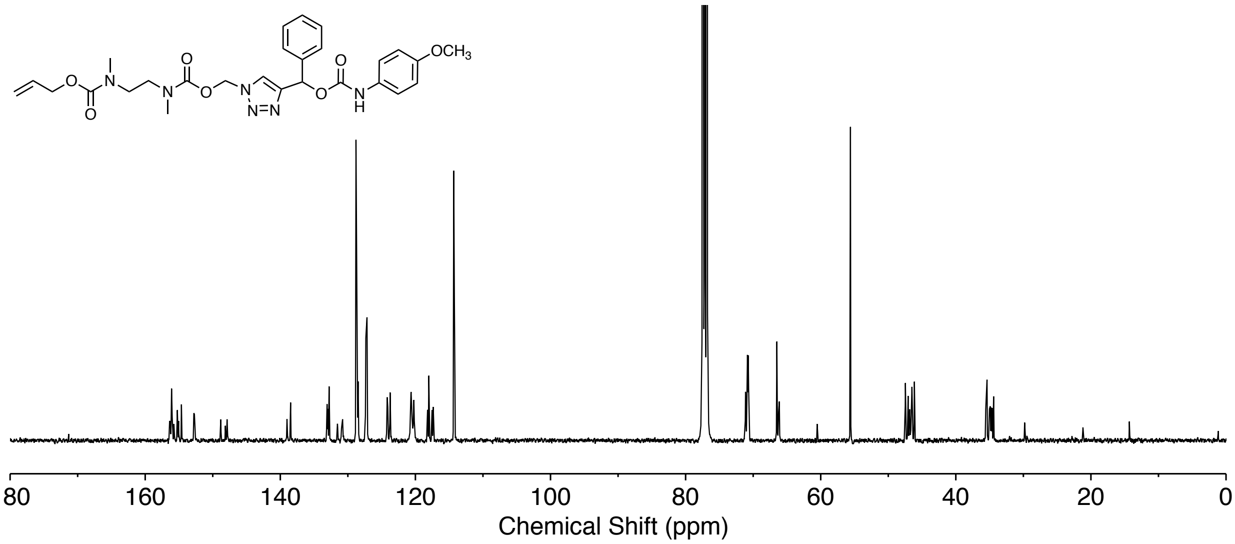

Figure S54. ^{13}C NMR spectrum (126 MHz, $CDCl_3$, 298 K) of **1d**. Sample contains trioxane standard.

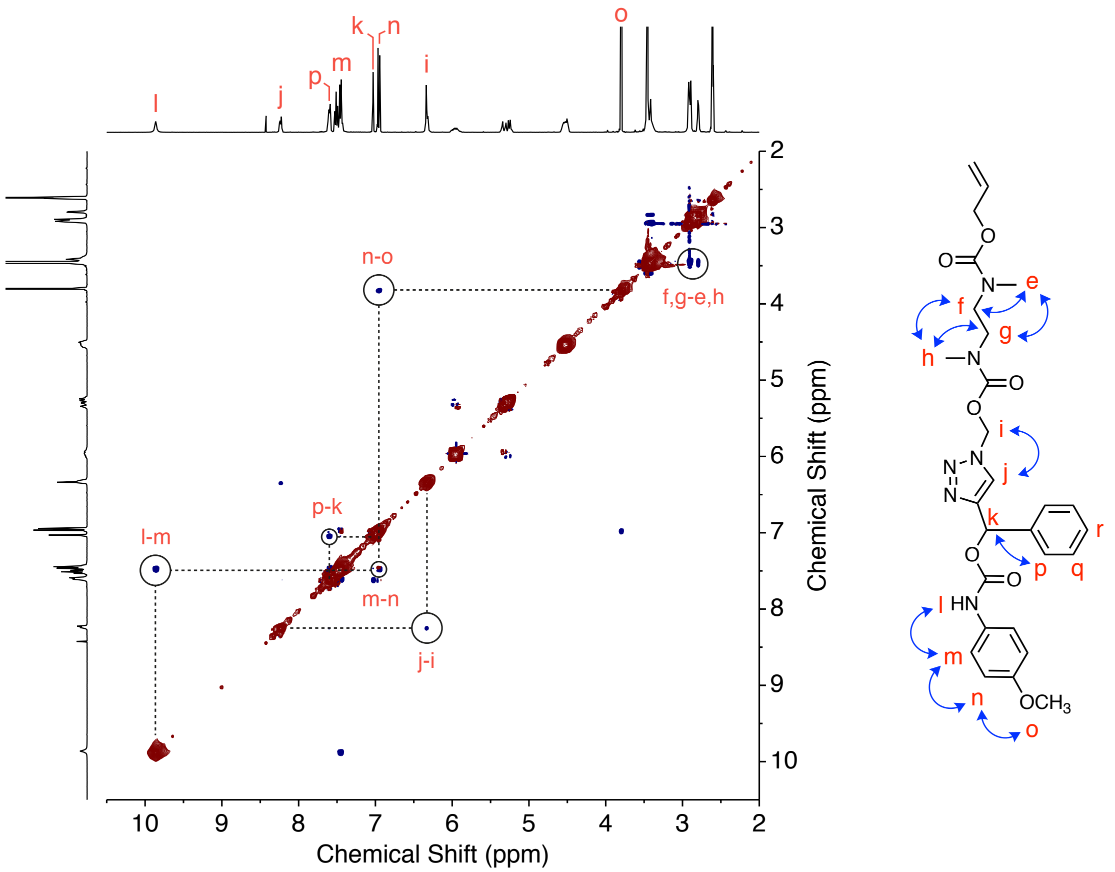

Figure S55. ^1H-^1H NOESY NMR spectrum (400 MHz, DMSO-d_6, 294 K; T_{mix} = 500 ms) of compound **1d**. ^1H assignment was achieved using the highlighted NOEs. (Baseline: Whittaker smoother; window functions: [f_1] sine square 90°, [f_2] sine square 90°; COSY-like symmetrization applied to reduce noise, validated by visual inspection).

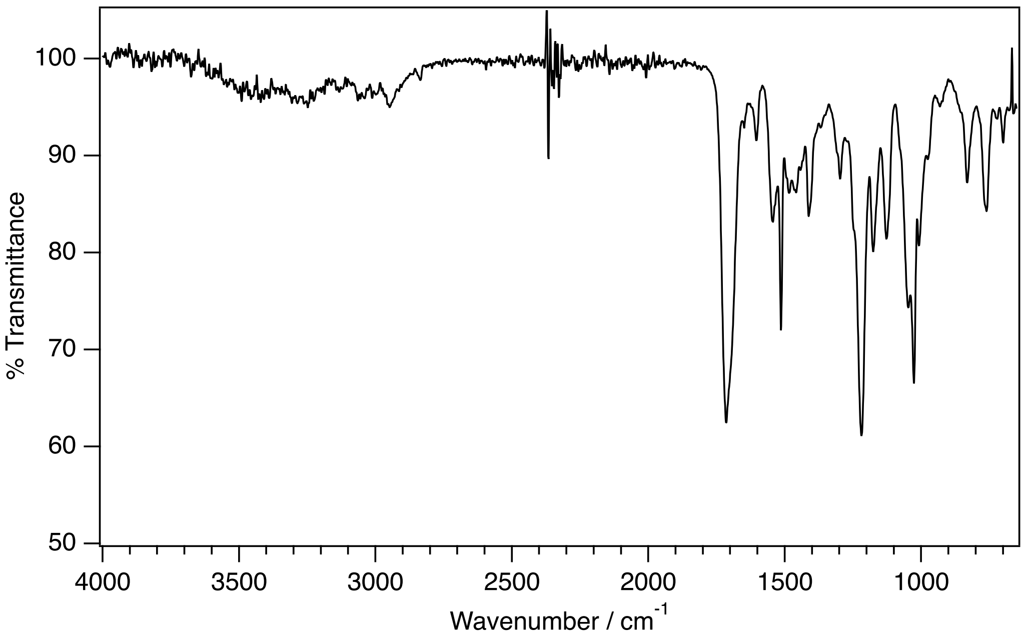

Figure S56. FTIR spectrum (ATR, solid powder) of compound **1d.**

Monoisotopic Mass, Even Electron Ions
43 formula(e) evaluated with 1 results within limits (all results (up to 1000) for each mass)
Elements Used:
C: 13-27 H: 1-33 N: 1-6 O: 0-7
JRN_45356 B Pilgrim BSP-B07-08 1912 (4.112)

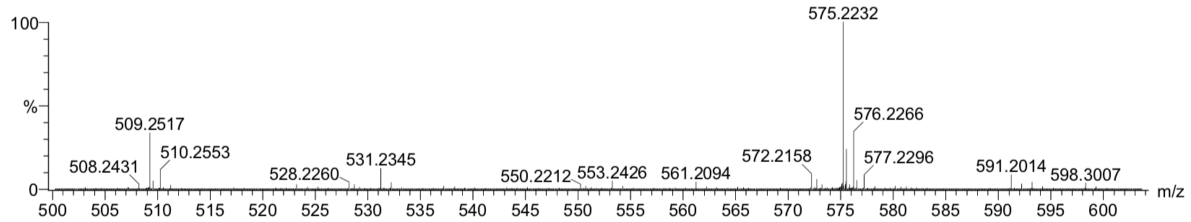

Minimum:				-1.5				
Maximum:		5.0	50.0	50.0				
Mass	Calc. Mass	mDa	PPM	DBE	i-FIT	Norm	Conf(%)	Formula
553.2426	553.2411	1.5	2.7	14.5	951.0	n/a	n/a	C27 H33 N6 O7

Figure S57. HRMS spectrum (TOF MS ASAP +ve) and analysis report for compound **1d**.

S5.5. Characterization data for Model 1e

The product was obtained as a pale yellow, highly viscous oil (283 mg, 496 µmol, 40%). **^{1}H NMR** (500 MHz, DMSO-d_6) δ_H 9.78 (s, 1H), 8.30 – 8.09 (m, 1H), 7.54 – 7.41 (m, 1H), 7.40 – 7.28 (m, 4H), 7.22 – 7.14 (m, 1H), 6.95 (s, 1H), 6.85 (d, J = 9.0 Hz, 2H), 6.31 – 6.16 (m, 2H), 5.95 – 5.78 (m, 1H), 5.31 – 5.08 (m, 2H), 4.54 – 4.30 (m, 2H), 3.70 (s, 3H), 3.45 – 3.23 (m, 4H), 2.89 – 2.64 (m, 6H). **^{13}C NMR** (126 MHz, DMSO-d_6) δ_C 162.09 (d, $^{1}J_{C\text{-}F}$ = 243.9 Hz), 155.31, 155.10, 154.93, 154.16, 153.88, 152.34, 146.23, 141.86, 134.19 – 132.91 (m), 131.77, 130.91 – 129.83 (m), 125.74 – 124.08 (m), 122.91, 119.74, 117.57 – 115.89 (m), 115.17 – 114.73 (m), 113.98, 113.83 – 113.25 (m), 70.70, 68.63, 66.01 – 63.38 (m), 55.15, 48.67 – 45.04 (m), 35.60 – 32.69 (m). **^{19}F NMR** (376 MHz, DMSO-d_6) δ −115.18. **FTIR** (ATR, solid powder) ν_{max} 3313, 2938, 1699, 1593, 1513, 1410, 1362, 1296, 1209, 1173, 1123, 1027, 972, 926, 829, 770, 686 cm^{-1}. **HRMS** (TOF MS ASAP +*ve*) *m*/*z* calculated for $C_{27}H_{32}N_6O_7F$ 571.2317, found 571.2328.

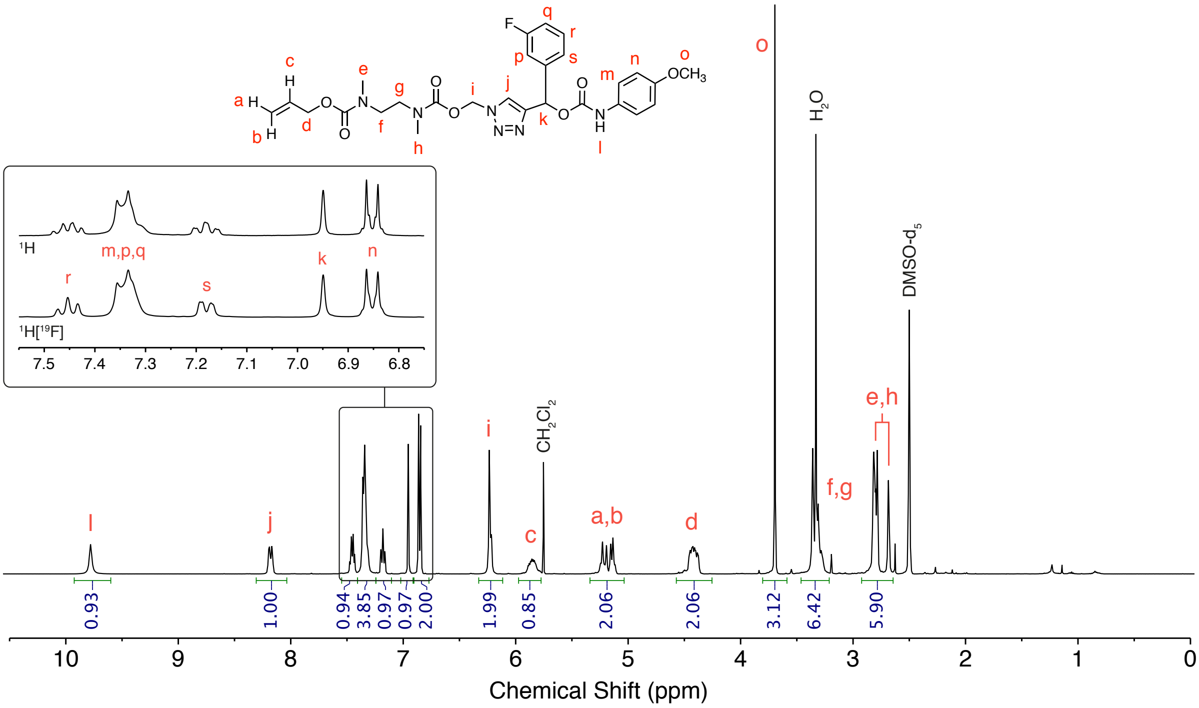

Figure S58. ^{1}H NMR (400 MHz, DMSO-d_6, 298 K) spectrum of compound **1e**. *Inset*: comparison of ^{1}H and ^{19}F-decoupled ^{1}H NMR spectra highlights the ^{1}H-^{19}F coupling for H_r and H_s.

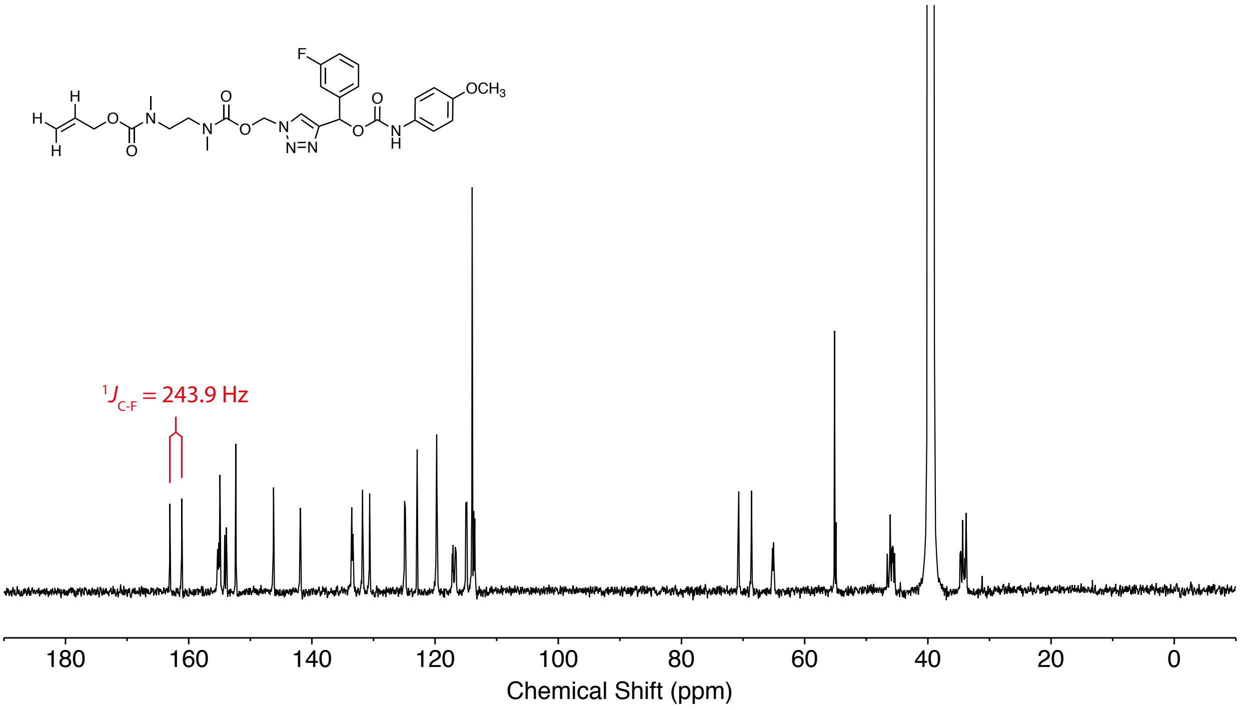

Figure S59. ^{13}C NMR (126 MHz, DMSO-d_6, 298 K) spectrum of compound **1e**.

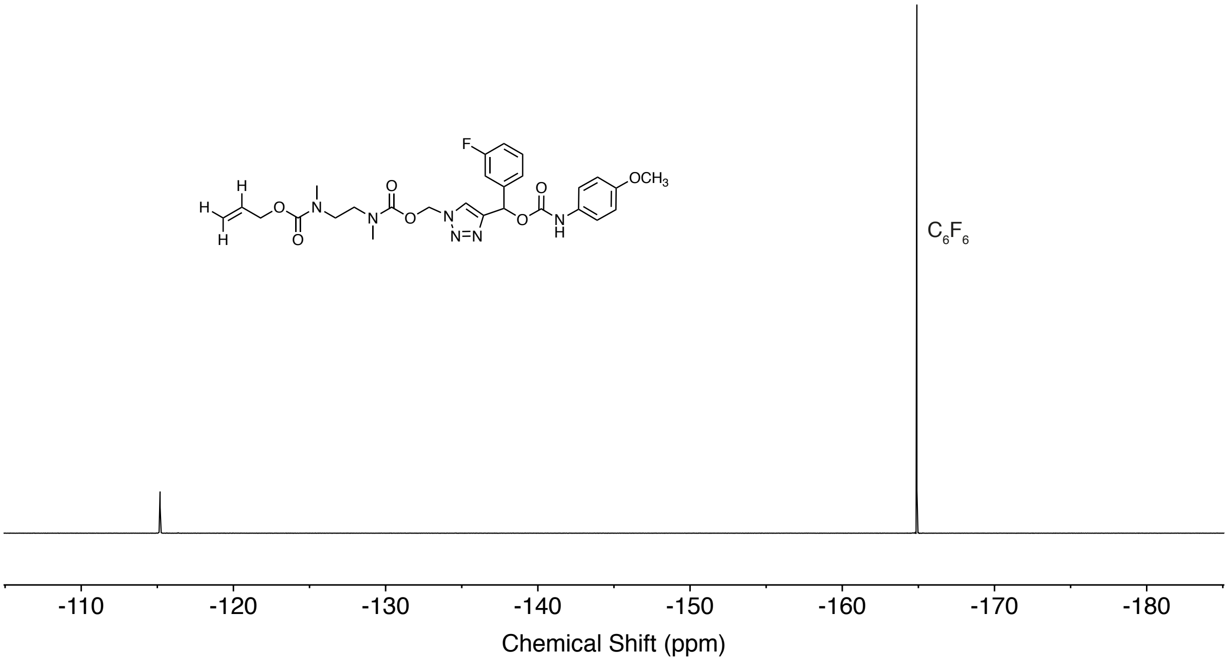

Figure S60. ^{19}F NMR (376 MHz, DMSO-d_6, 298 K) spectrum of compound **1e**.

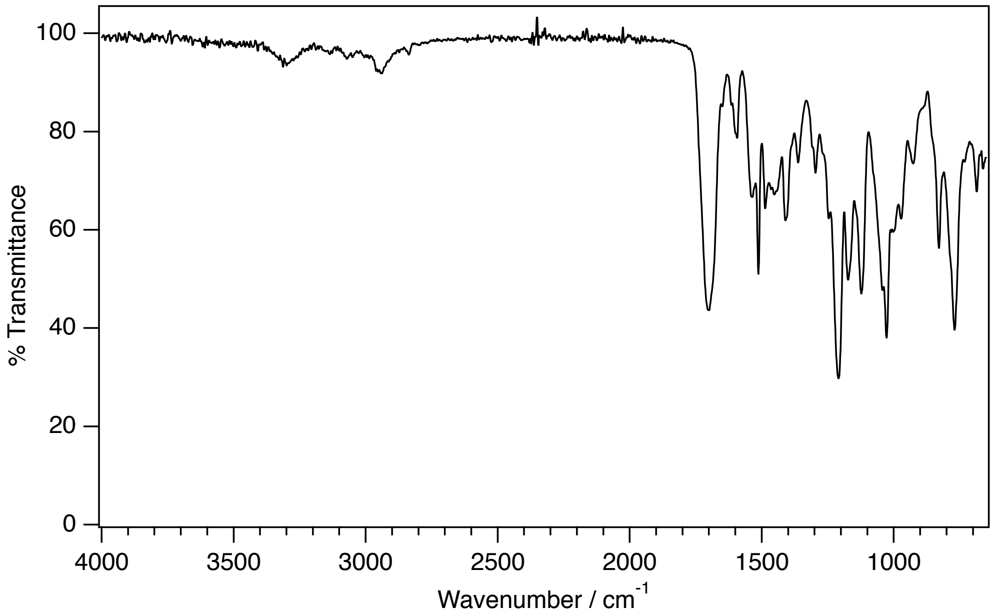

Figure S61. FTIR spectrum (ATR, solid powder) of compound **1e**.

Monoisotopic Mass, Even Electron Ions
43 formula(e) evaluated with 1 results within limits (all results (up to 1000) for each mass)
Elements Used:
C: 13-27 H: 1-32 N: 1-6 O: 0-7 F: 1-1

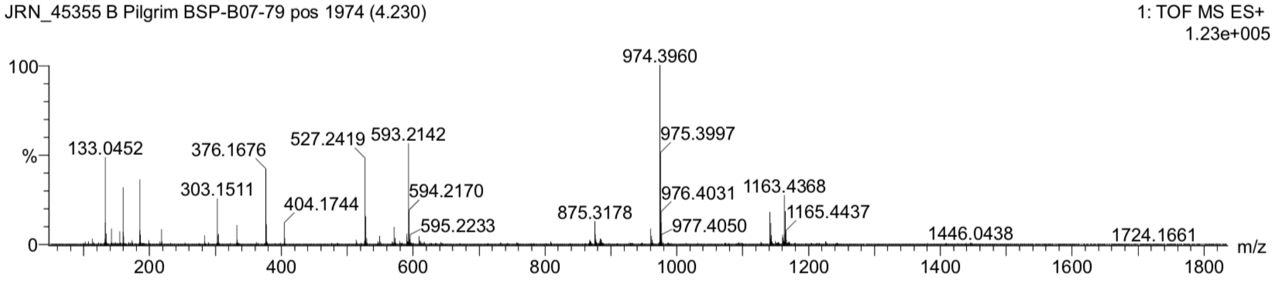

Minimum:				-1.5				
Maximum:		5.0	50.0	50.0				
Mass	Calc. Mass	mDa	PPM	DBE	i-FIT	Norm	Conf(%)	Formula
571.2328	571.2317	1.1	1.9	14.5	264.4	n/a	n/a	C27 H32 N6 O7 F

Figure S62. HRMS spectrum (TOF MS ASAP +ve) and analysis report for compound **1e**.

S5.6. Characterization data for Model 1f

The product was obtained as a colorless, highly viscous oil (296 mg, 508 µmol, 89%). ^{1}**H NMR** (400 MHz, DMSO-d_6) δ 9.76 (s, 1H), 8.22 – 7.95 (m, 1H), 7.42 – 7.23 (m, 3H), 7.14 – 6.97 (m, 2H), 6.97 – 6.72 (m, 4H), 6.33 – 6.10 (m, 2H), 5.95 – 5.74 (m, 1H), 5.34 – 5.02 (m, 2H), 4.53 – 4.30 (m, 2H), 3.75 (d, J = 0.8 Hz, 3H), 3.69 (s, 3H), 3.34 (m, 4H), 2.91 – 2.62 (m, 6H). ^{13}**C NMR** (126 MHz, DMSO-d_6) δ 159.29, 155.49 – 155.24 (m), 155.10, 154.88, 154.16, 153.89, 152.47, 146.73, 140.61, 133.96 – 133.03 (m), 131.87, 129.65, 125.82 – 123.78 (m), 119.70, 118.95, 117.54 – 116.17 (m), 113.96, 113.28, 112.66, 70.95 – 70.72 (m), 70.68, 69.27, 66.11 – 64.35 (m), 56.53 – 53.23 (m), 47.69 – 44.08 (m), 35.41 – 32.89 (m). **FTIR** (ATR, solid powder) ν_{max} 3290, 2937, 1696, 1601, 1513, 1488, 1457, 1410, 1210, 1172, 1123, 1027, 972, 929, 829, 769, 732, 696 cm^{-1}. **LRMS** (+*ve* ESI-LCMS, CH_3CN/water/CF_3COOH) *m*/*z* 605.1 ([M+Na]$^+$ 100%). **HRMS** (TOF MS ASAP +*ve*) *m*/*z* calculated for $C_{28}H_{35}N_6O_8$ 583.2516, found 583.2531.

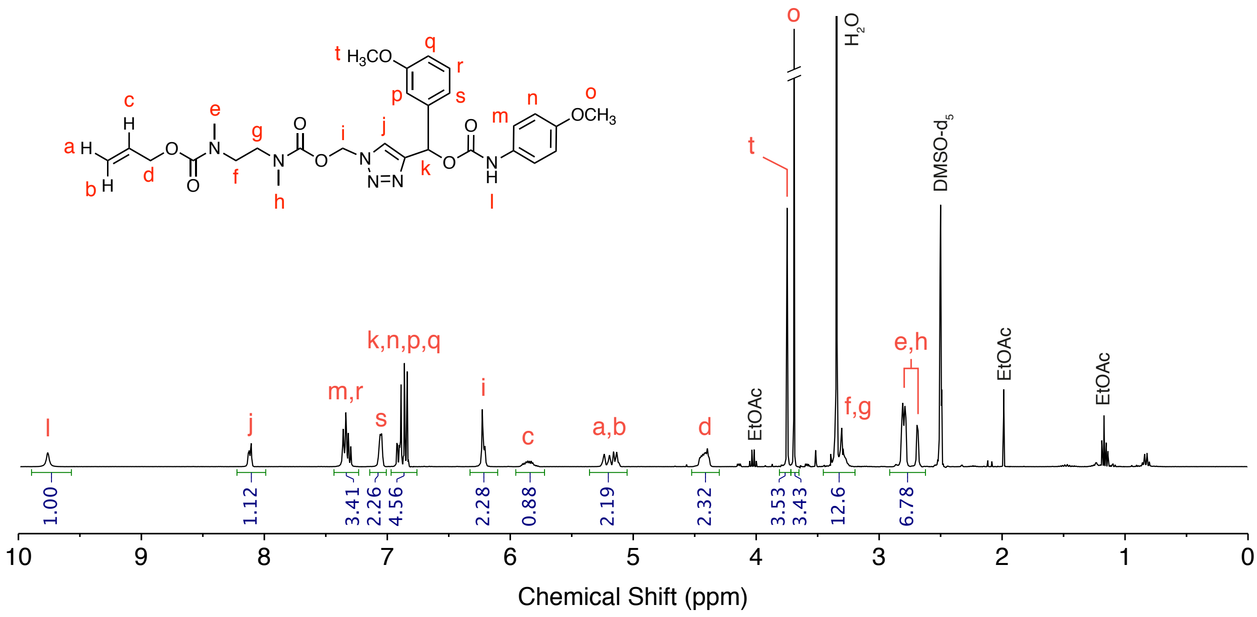

Figure S63. ^{1}H NMR (400 MHz, DMSO-d_6, 298 K) spectrum of compound **1f**.

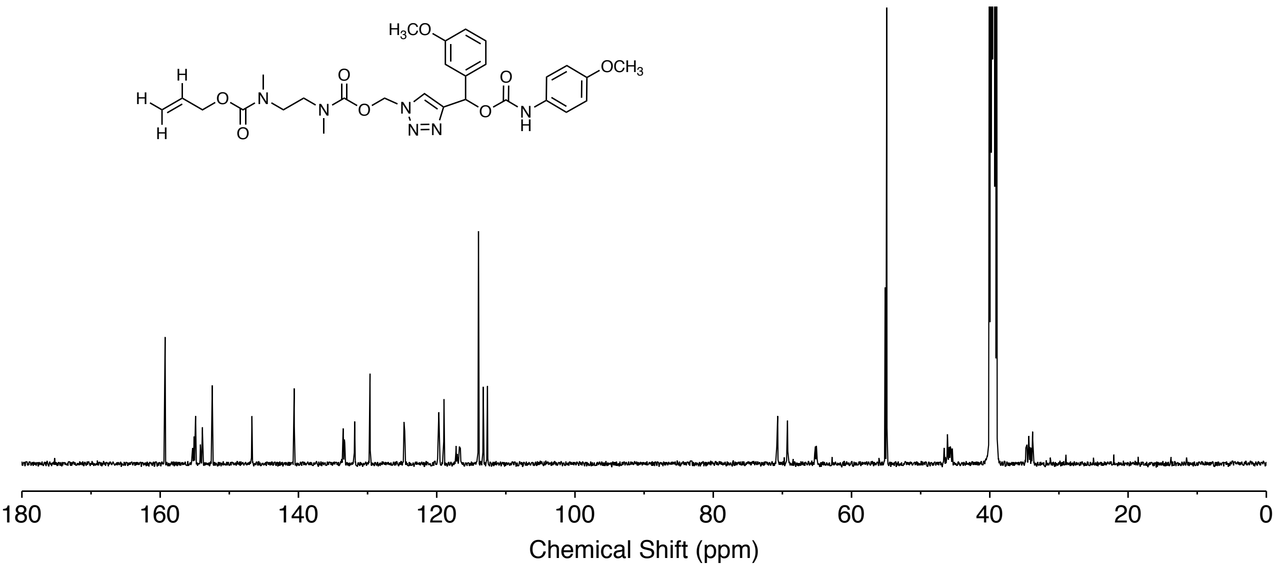

Figure S64. ^{13}C NMR (126 MHz, DMSO-d_6, 298 K) spectrum of compound **1f**.

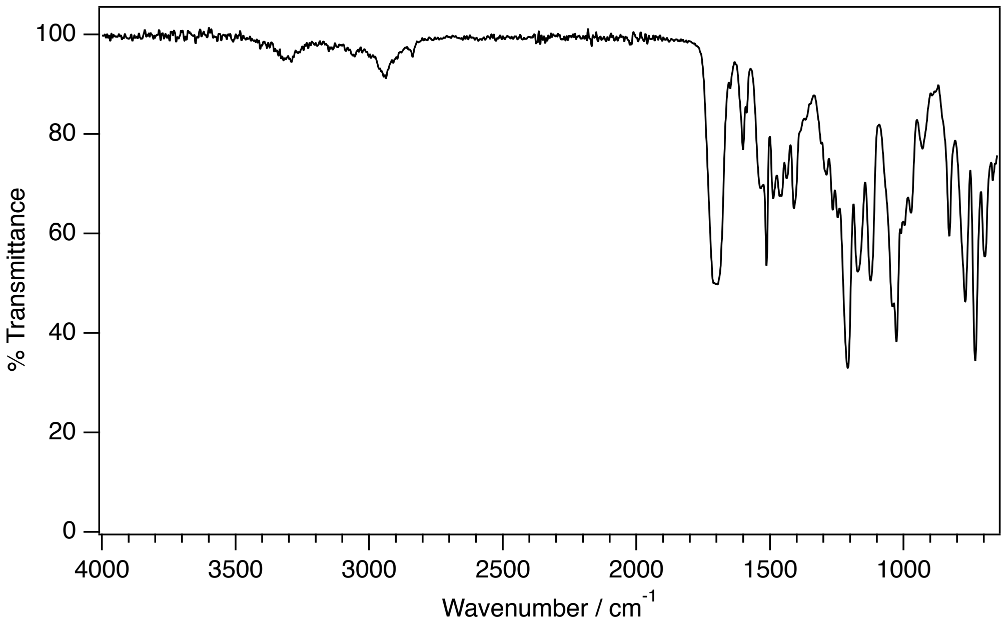

Figure S65. FTIR spectrum (ATR, solid powder) of compound **1f**.

Monoisotopic Mass, Even Electron Ions
49 formula(e) evaluated with 1 results within limits (all results (up to 1000) for each mass)
Elements Used:
C: 15-28 H: 1-35 N: 1-6 O: 0-8

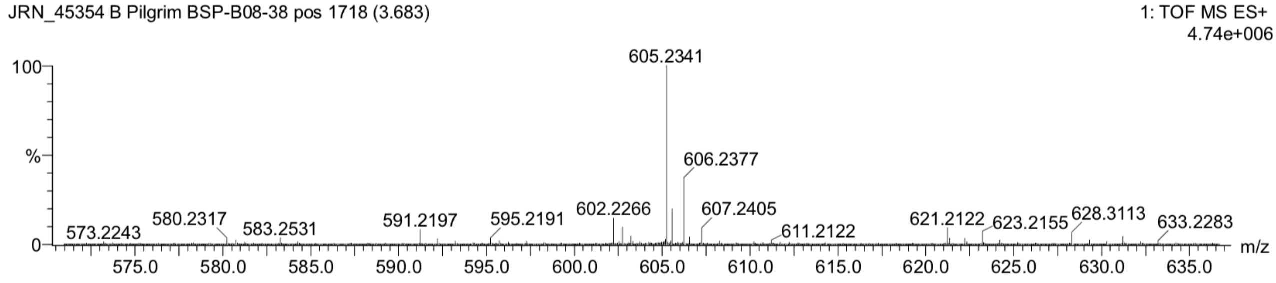

Minimum:				-1.5				
Maximum:		5.0	50.0	50.0				
Mass	Calc. Mass	mDa	PPM	DBE	i-FIT	Norm	Conf(%)	Formula
583.2531	583.2516	1.5	2.6	14.5	899.2	n/a	n/a	C28 H35 N6 O8

Figure S66. HRMS spectrum (TOF MS ASAP +ve) and analysis report for compound **1f**.

S6. Self-Immolation Kinetics of Models 1a–f in DMSO-d_6

Detailed analyses of the self-immolation kinetics of all model compounds (**1a**–**1f**) were performed in DMSO-d_6 in order to initially elucidate the mechanism of self-immolation in a non-nucleophilic solvent prior to analysis in mixed organic-aqueous systems. Full NMR spectra and kinetics analysis are shown in Section S6.7.

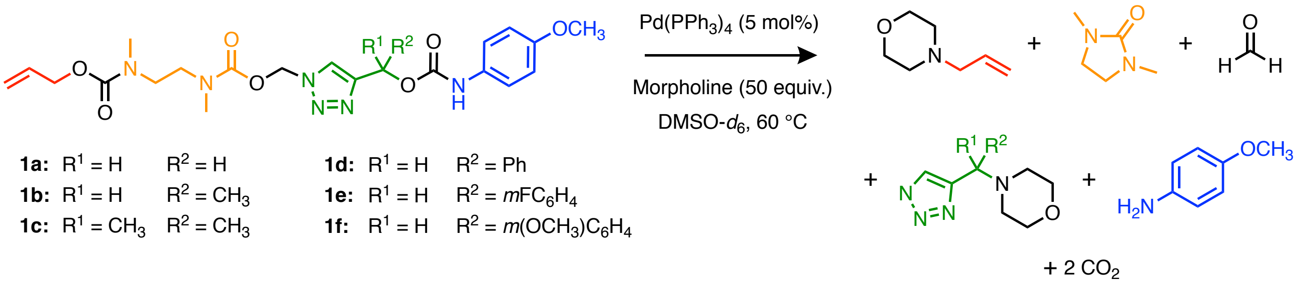

Scheme S3. General scheme for Pd^0-triggered self-immolation of model compounds **1a**–**f** under pseudo-first-order conditions at 60 °C, monitoring the reaction kinetics by *in situ* ^{1}H NMR spectroscopy.

S6.1. General method

In a typical experiment, a standard 5 mm NMR tube was charged with a solution of model compound in DMSO (16 µmol, 150 mM solution), morpholine (71 µL, 815 µmol, 50 equiv.) DMSO-d_6 (made up to a final volume of 500-550 µL) containing a 1,3,5-trioxane internal standard (~3 mM final concentration). The tube was inserted into the NMR spectrometer, equilibrated at 60 °C until stable (~5 min) then the spectrometer was tuned and matched, locked and shimmed. An initial spectrum was collected for concentration calibration (typically *ds* = 2, *ns* = 8, running a *zg30* pulse program with a *D1* recycle delay of 2 s to ensure complete longitudinal relaxation of the sample between each scan to enable quantitative integration of the spectrum). The tube was ejected from the spectrometer, a suspension of $Pd(PPh_3)_4$ (~1 µmol) in DMSO-d_6 (100 µL) added and the tube mixed rapidly before returning the sample to the spectrometer. The sample was generally left out of the spectrometer for <1 min, remaining within 5 °C of the target temperature. Upon returning to the spectrometer, the sample was re-shimmed and kinetics timepoints were collected immediately. The frequency at which spectra were collected was controlled by varying the number of dummy scans.

S6.2. Kinetics methodology for model 1a

Due to the slow rate of self-immolation for model compound **1a**, kinetics data were collected in two phases. Data were collected continuously for the first 15 min of the experiment, allowing us to capture the initial trigger cleavage and cyclisation steps. However, due to the much slower rate of the 1,4-elimination phase, it was not possible to continuously record data *in situ*. Consequently, subsequent timepoints for sample **1a** were recorded by transferring the NMR tube between the spectrometer and an oil bath maintained at 60 °C, periodically removing the sample for no longer than 15 min to record spectra at either 25 or 60 °C.[1] Processed NMR and kinetics data are shown Section S6.7, below.

[1]Some of the time points were recorded at 25 °C on an open-access autosampling 400 MHz spectrometer due to limited availability of the variable temperature 400 MHz spectrometer during the course of this experiment. Recorded spectra at different temperatures did not measurably influence the concentrations of species during self-immolation, and therefore did not interfere with integration/kinetics analysis.

S6.3. Kinetics methodology for models 1b-f

NMR kinetics experiments were performed using the same procedure as model **1a**, except the data for models **1b–f** were collected in a single continuous sitting. Processed NMR and kinetics data are shown in Section S6.7, below.

S6.4. Kinetics analysis methodology

NMR spectra were digitally processed and automatically integrated using the arrayed data analysis tool in Mestrenova 14.0. This allows us to export the integration data for several peaks within the spectrum as a single matrix with time as the independent variable. A typical example of the data analysis window is shown in Figure S67.

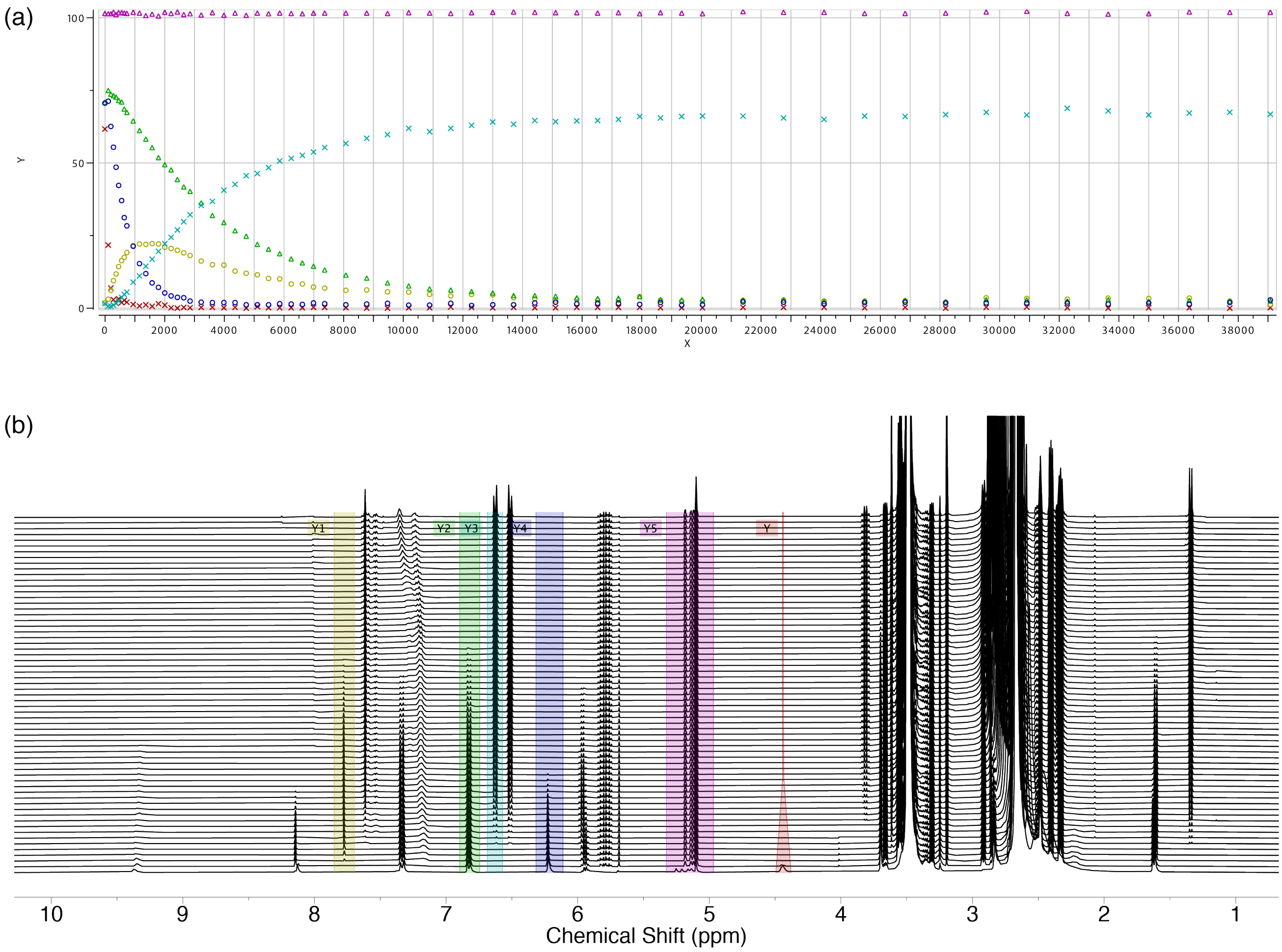

Figure S67. Representative example of the data analysis window in Mestrenova 14.0, showing (a) the extracted integrals plotted as a function of time and (b) the stacked time course spectra with colored integration regions. Data for model **1c** self-immolation kinetics in DMSO-d_6 at 333 K are shown.

Integrals were references against the singlet peak of 1,3,5-trioxane (~5.3 ppm), which was included as an internal concentration standard. Due to overlapping of this singlet with peaks of allyl-containing compounds, the integral component of this peak cluster (Y5 in Figure S67) was computed by multiple peak fitting analysis in Mestrenova. Upon close manual inspection of the data analysis outputs, we found that the integration window had to be narrowed when peak integrals approached zero in order to obtain reliable integration data (i.e., having the integration window too wide when the peaks were

very small resulted in the program erroneously inflating the value of the integrals when compared with manual integration). Hence, the colored integration windows in Figure S67 are tapered.

The mechanism of self-immolation was assumed to be first order in self-immolative species in the elimination cascade. In generic terms, the key elementary rate operations are summarised in Figure S68.

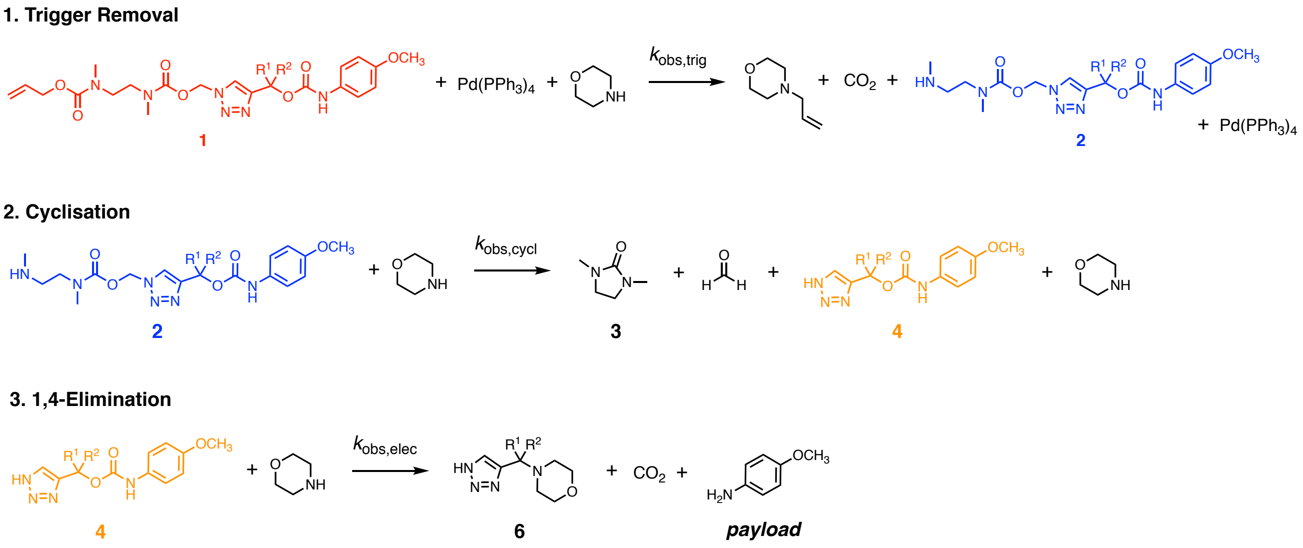

Figure S68. Summary of the key observable elementary rate operations in the self-immolation cascades of compounds **1a–f**.

Each of the above steps are base-mediated, so the rate expressions will depend on the concentration of the self-immolative species (**1**, **2** or **4**) and the concentration of base. However, since the self-immolation reactions were performed using a large excess of morpholine (50 equiv.), the corresponding rate expressions for these steps simplify to pseudo-first-order rate laws, as described in Equations S1–S3:

$$-\frac{d[\mathbf{1}]}{dt} = k_{\text{obs,trig}}[\mathbf{1}] \qquad \text{where } k_{\text{obs,trig}} = k[\text{morpholine}][(\text{PdPPh}_3)_4] \qquad \text{(Equation S1)}$$

$$-\frac{d[\mathbf{2}]}{dt} = k_{\text{obs,cycl}}[\mathbf{2}] \qquad \text{where } k_{\text{obs,cycl}} = k[\text{morpholine}] \qquad \text{(Equation S2)}$$

$$-\frac{d[\mathbf{4}]}{dt} = k_{\text{obs,1,4elim}}[\mathbf{4}] \qquad \text{where } k_{\text{obs,elec}} = k[\text{morpholine}] \qquad \text{(Equation S3)}$$

A relatively high catalyst loading was used for the initial trigger cleavage step to ensure the catalyst was operating at its maximum velocity. Under pseudo-first-order conditions, the consumption of species **1**, **2** and **4** are therefore described by a monoexponential decay process (Equation S4) according to the integrated rate expression for a first-order reaction:

$$I_n = A\exp\left(\frac{-(t - t_0)}{\tau}\right) \qquad \text{where} \quad \tau = \frac{1}{k_{\text{obs}}} \qquad \text{(Equation S4)}$$

Where I_n is the measured integral of a given nucleus n, A is a pre-exponential scaling factor, t is the independent time variable, t_0 is the extrapolated value of time at $t = 0$ s, and τ is the exponential decay constant that is reciprocally related to the pseudo-first-order rate constant.

Fitting analysis was performed in IgorPro 7 using the standard curve fitting package, which uses a non-linear least-squares regression process to optimize the fits. In general, the kinetics data were well described by the pseudo-first-order model described in Equation S4, which is consistent with the expected fragmentation mechanism in the presence of excess base. Early timepoints were not included in the fitting procedure in order to exclude parts of the data where the samples had not yet re-equilibrated to 60 °C after addition of the catalyst suspension and re-insertion into the NMR spectrometer. Kinetics plots for all experimental data are show in Section S6.7, below.

S6.5. Reference NMR spectra (reagents and products)

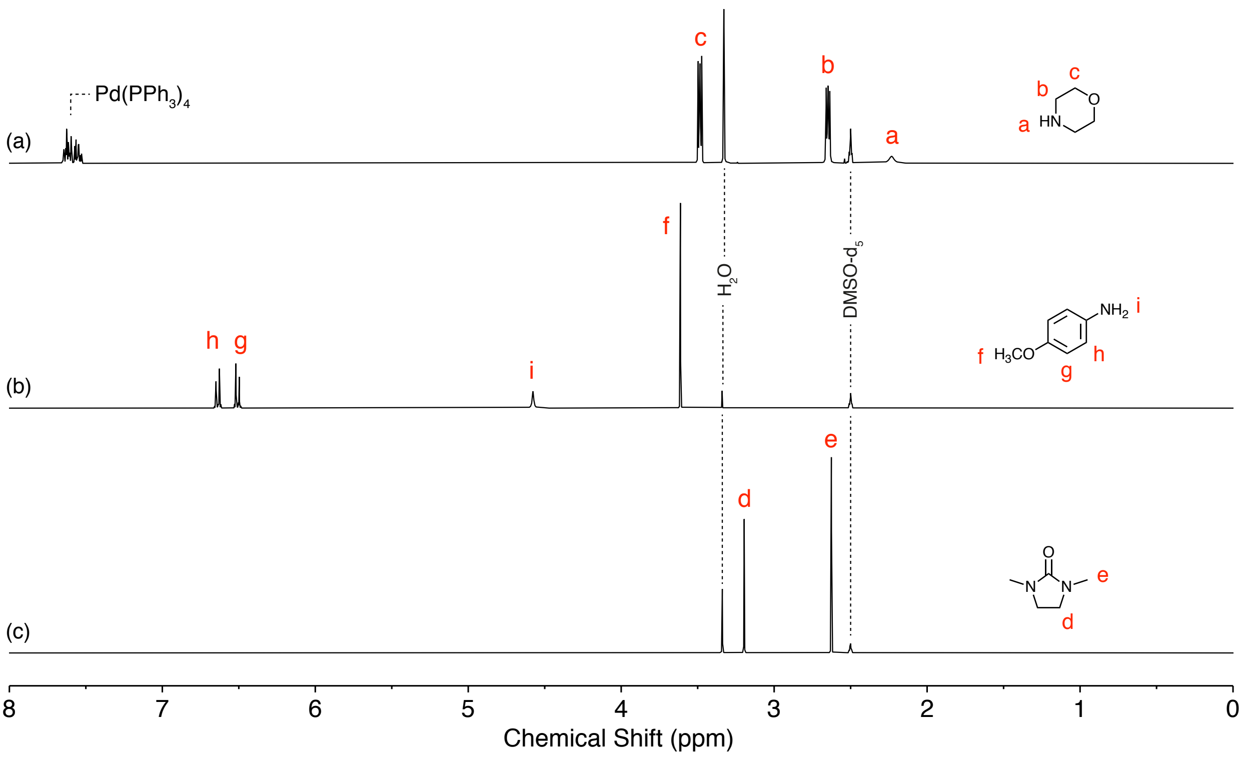

Figure S69. ^{1}H NMR spectra (400 MHz, DMSO-d_6, 295 K) of (a) $Pd(PPh_3)_4$ and morpholine, (b) *p*-anisidine, and (c) 1,3-dimethylimidazolidin-2-one.

S6.6. Self-immolation control experiments for 1c

To confirm that self-immolation was triggered only by the addition of $Pd(PPh_3)_4$, and that no appreciable degradation occurred under basic conditions alone, a control experiment using the least stable model compound (**1c**) was carried out as a representative test. A solution of model **1c** in DMSO-d_6 (35 mM) containing morpholine (50 equiv.) was heated at 60 °C for 3.75 h and monitored by *in situ* ^{1}H NMR spectroscopy. No degradation of the compound was observed under these conditions (Figure S70), suggesting that any direct hydrolysis of the model compounds is much slower than the rate of self-immolative elimination. Furthermore, good agreement between the experimental data and the first-order kinetics model suggests that self-immolation is the dominant pathway of degradation in the kinetics experiments reported herein.

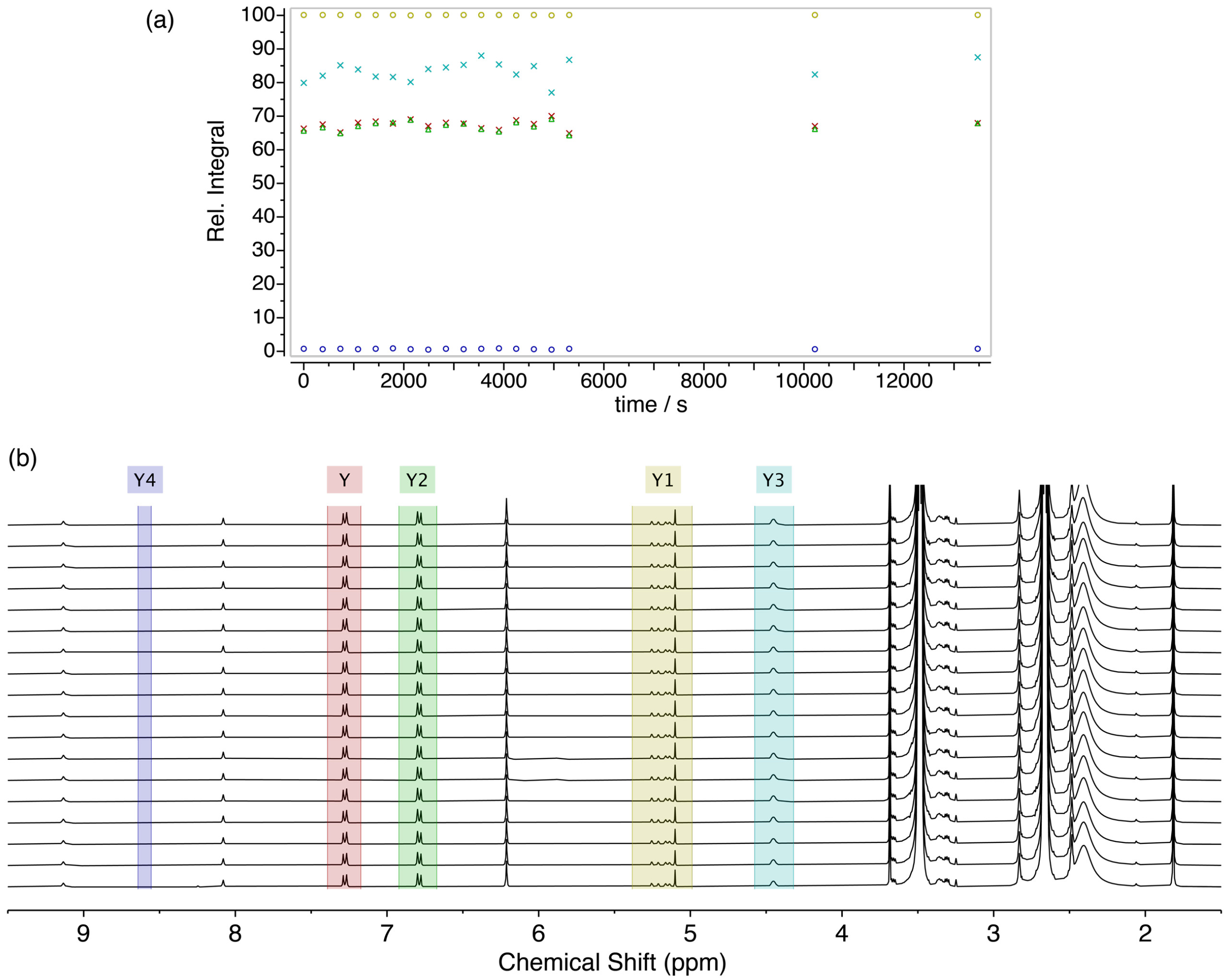

Figure S70. ^{1}H NMR kinetics control experiment (400 MHz, DMSO-d_6, 333 K) of model **1c** heated with morpholine for 3.75 h, showing no observable hydrolysis or degradation in the absence of $Pd(PPh_3)_4$. (a) Relative integral plotted against time. (b) Stacked NMR spectra showing integrated regions. Region Y4 was included to extend the y-axis scale to zero in Figure S70a.

S6.7. Self-immolation kinetics data for 1a–f

Table S2. Summary of pseudo-first-order rate constants (k_{obs}) for the three stages of self-immolation for model compounds **1a–f** in DMSO-d_6 at 60 °C.

Compound	Trigger Removal (s^{-1})	Cyclisation (s^{-1})	1,4-elimination (s^{-1})
**1a**	–[a]	$(1.74 \pm 0.03) \times 10^{-3}$	$(2.61 \pm 0.14) \times 10^{-6}$
**1b**	$(1.06 \pm 0.02) \times 10^{-2}$	$(1.53 \pm 0.01) \times 10^{-3}$	$(2.67 \pm 0.02) \times 10^{-4}$
**1c**	$(2.2 \pm 0.8) \times 10^{-2}$	$(1.51 \pm 0.05) \times 10^{-3}$	$(1.50 \pm 0.06) \times 10^{-3}$
**1d**	$(3.1 \pm 0.1) \times 10^{-2}$	$(1.85 \pm 0.02) \times 10^{-3}$	$(1.44 \pm 0.03) \times 10^{-3}$
**1e**	$(2.37 \pm 0.05) \times 10^{-2}$	$(1.95 \pm 0.02) \times 10^{-3}$	$(1.52 \pm 0.02) \times 10^{-3}$
**1f**	$(3.8 \pm 0.3) \times 10^{-2}$	$(1.80 \pm 0.02) \times 10^{-3}$	$(1.07 \pm 0.05) \times 10^{-3}$

[a]Trigger removal was complete within the equilibration and shimming period.

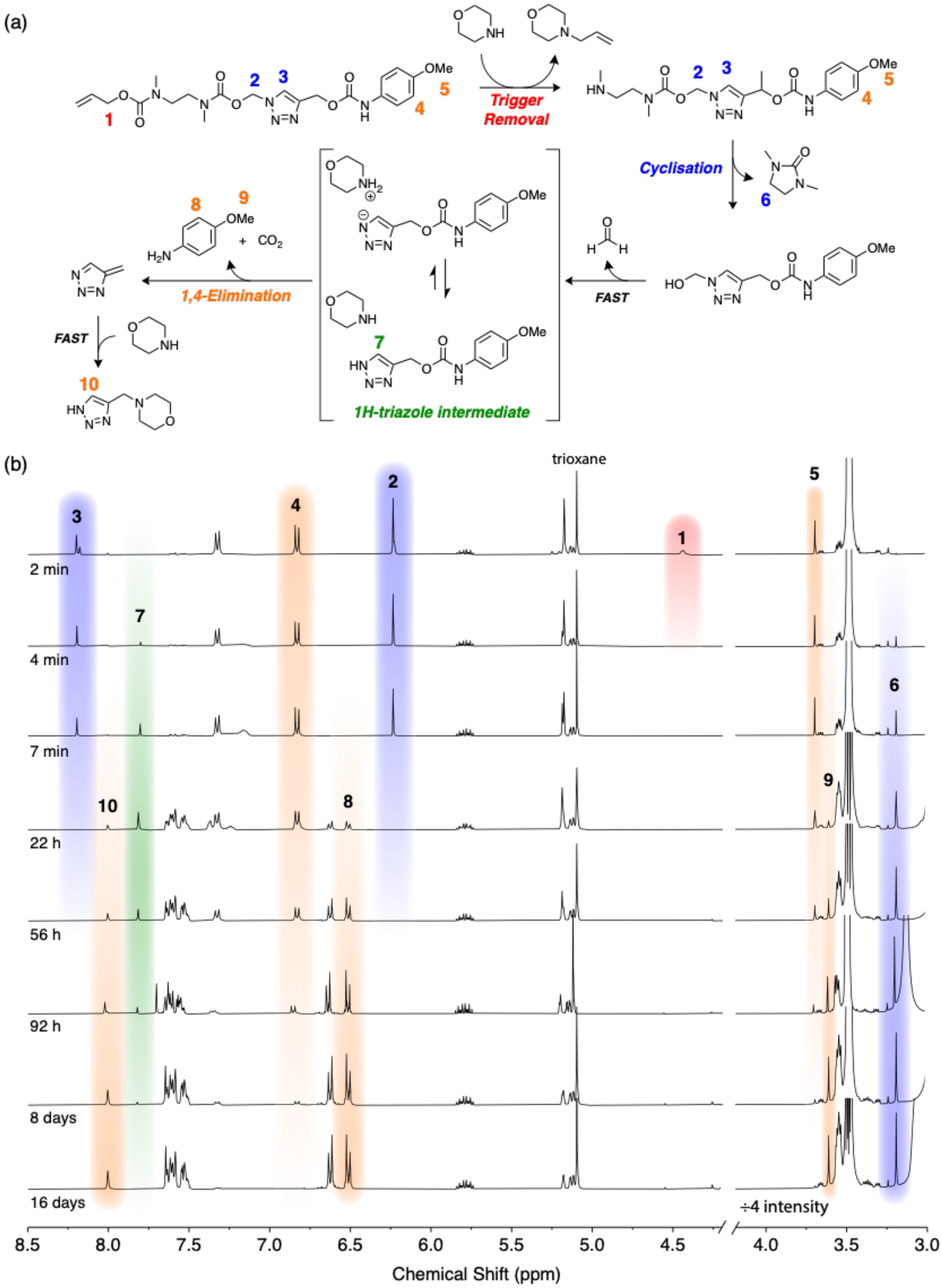

Figure S71. NMR kinetics for self-immolation of model **1a** in DMSO-d_6. (a) Reaction scheme showing key reactive species according to the proposed mechanism of self-immolative elimination. Numbered ^{1}H assignments are color-coded to match the peaks in spectra. (b) Stacked ^{1}H NMR spectra (400 MHz, DMSO-d_6, 333 K) showing a representative cross-section of the spectra recorded to construct the kinetics traces.

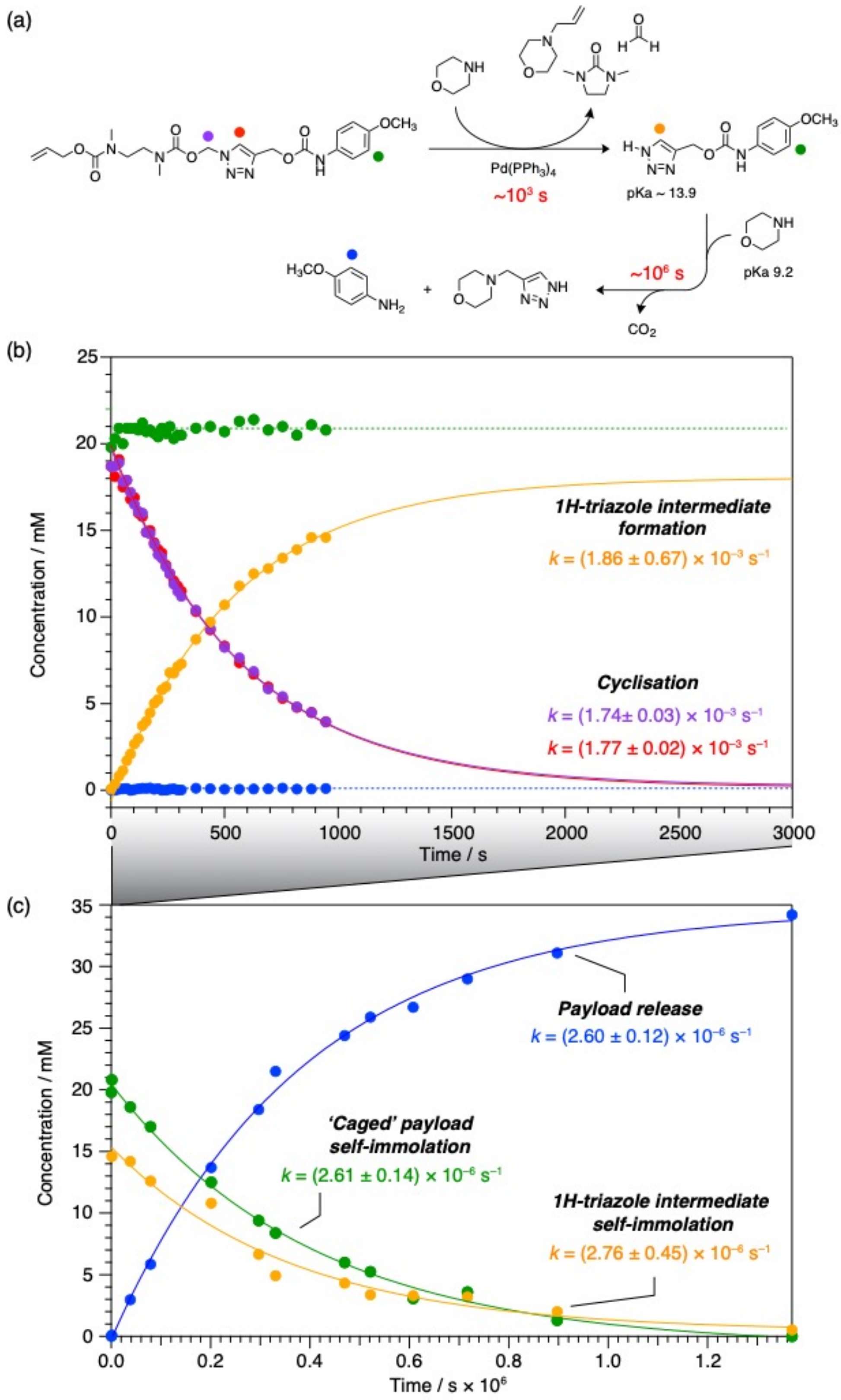

Figure S72. (a) Self-immolation mechanism of model **1a**, highlighting three distinct stages in the immolation cascade. Colored shapes denote ^{1}H nuclei monitored to obtain kinetics traces in (b) and (c). **(b)** NMR kinetics traces (DMSO-d_6, 333 K) showing loss of substrate signals. **(c)** NMR kinetics traces (DMSO-d_6, 333 K) showing appearance of immolation products. The trigger removal occurred faster than equilibration of the NMR sample. Solid lines depict monoexponential fits according to Equation S4, from which pseudo-first-order rate constants were determined.

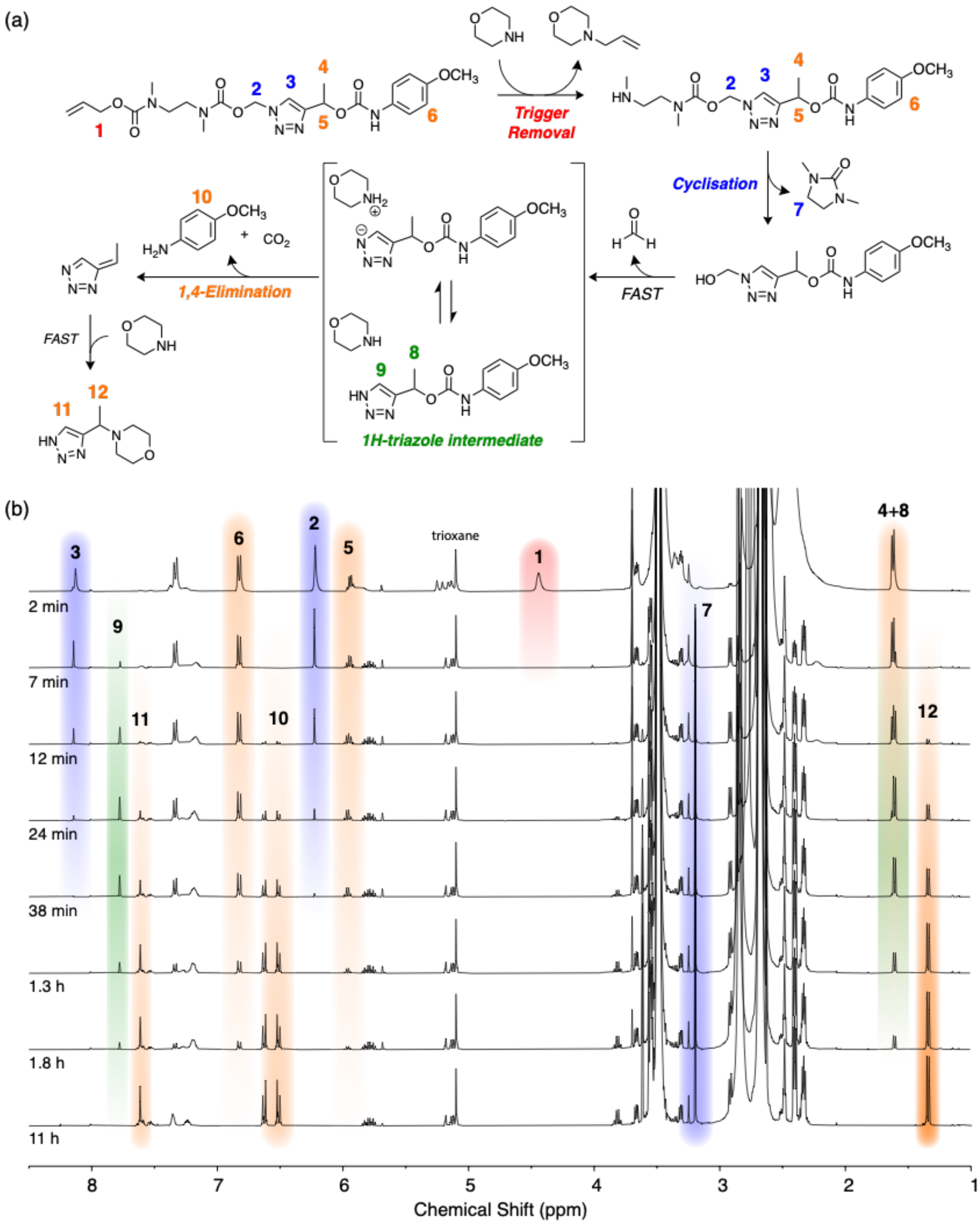

Figure S73. NMR kinetics for self-immolation of model **1b** in DMSO-d_6. (a) Reaction scheme showing key reactive species according to the proposed mechanism of self-immolative elimination. Numbered ^{1}H assignments are color-coded to match the peaks in spectra. (b) Stacked ^{1}H NMR spectra (400 MHz, DMSO-d_6, 333 K) showing a representative cross-section of the spectra recorded to construct the kinetics traces.

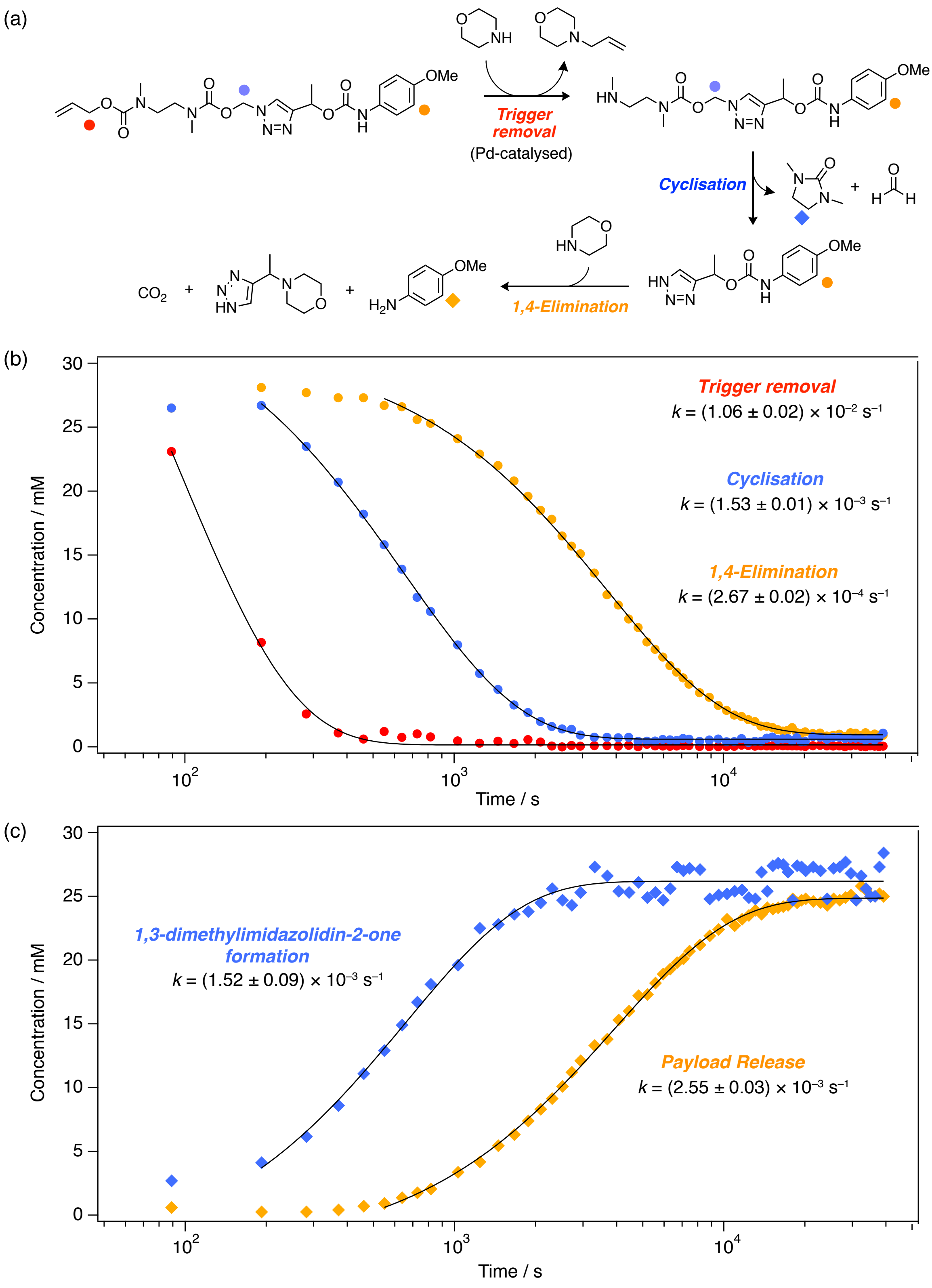

Figure S74. (a) Self-immolation mechanism of model **1b**, highlighting three distinct stages in the immolation cascade (trigger removal, cyclisation and electronic elimination). Colored shapes denote ^{1}H nuclei monitored to obtain kinetics traces in (b) and (c). **(b)** NMR kinetics traces (DMSO-d_6, 333 K) showing loss of substrate signals. **(c)** NMR kinetics traces (DMSO-d_6, 333 K) showing appearance of immolation products. The trigger removal product (*N*-allylmorpholine) formed too rapidly to monitor by NMR. Black lines are monoexponential fits according to Equation S4, from which pseudo-first-order rate constants were determined.

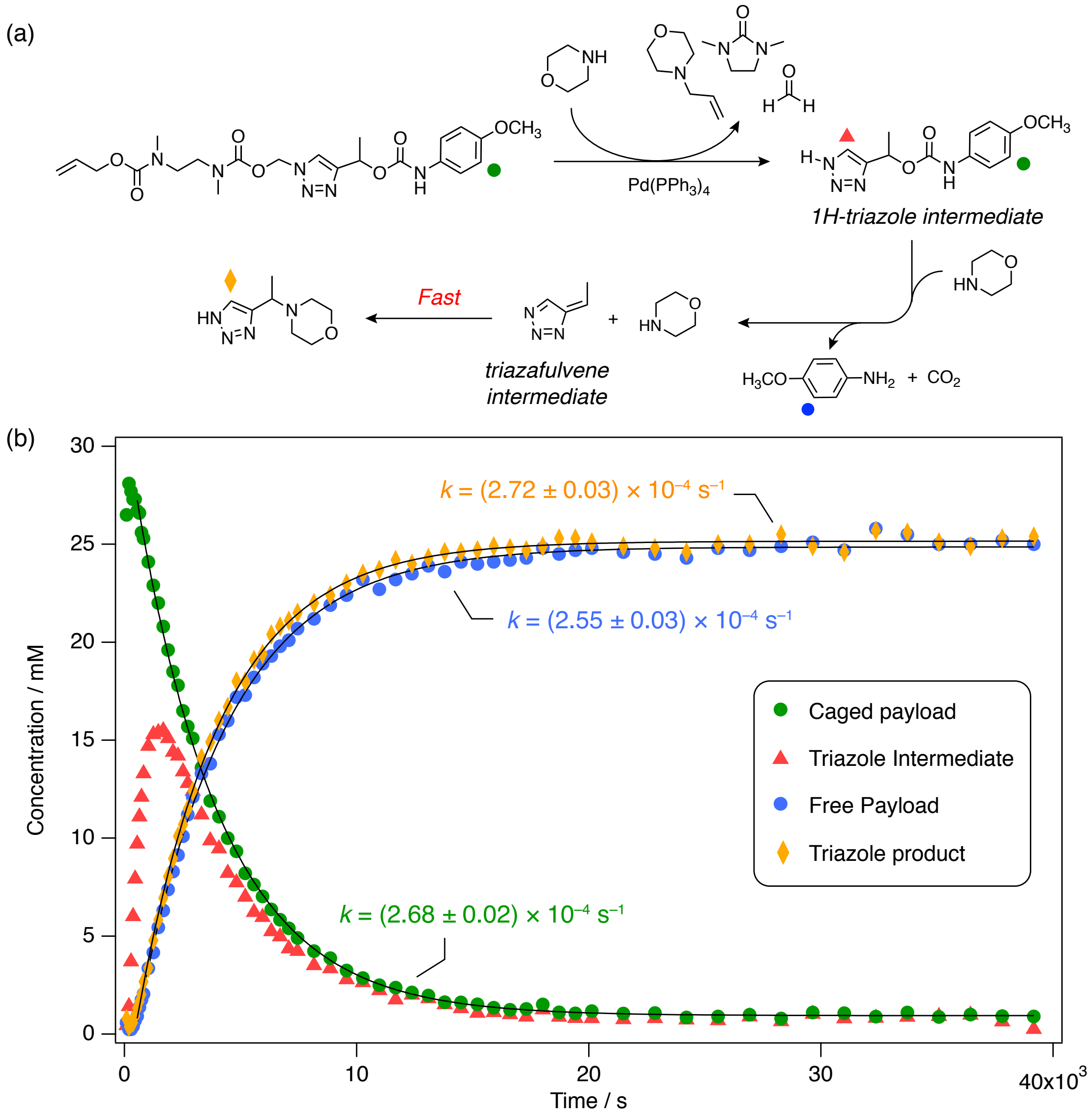

Figure S75. (a) Self-immolation mechanism of model **1b** in DMSO-d_6. Colored shapes denote ^{1}H nuclei monitored to obtain kinetics traces in (b). **(b)** NMR kinetics traces (DMSO-d_6, 333 K) showing the time-dependent product distribution during self-immolation of model **1b**. Black lines represent monoexponential fits according to the pseudo-first-order model described in Equation S4. Similar rates of formation of the free payload (blue circles) and triazole product (orange diamonds) confirms that the triazafulvene intermediate is rapidly intercepted by morpholine.

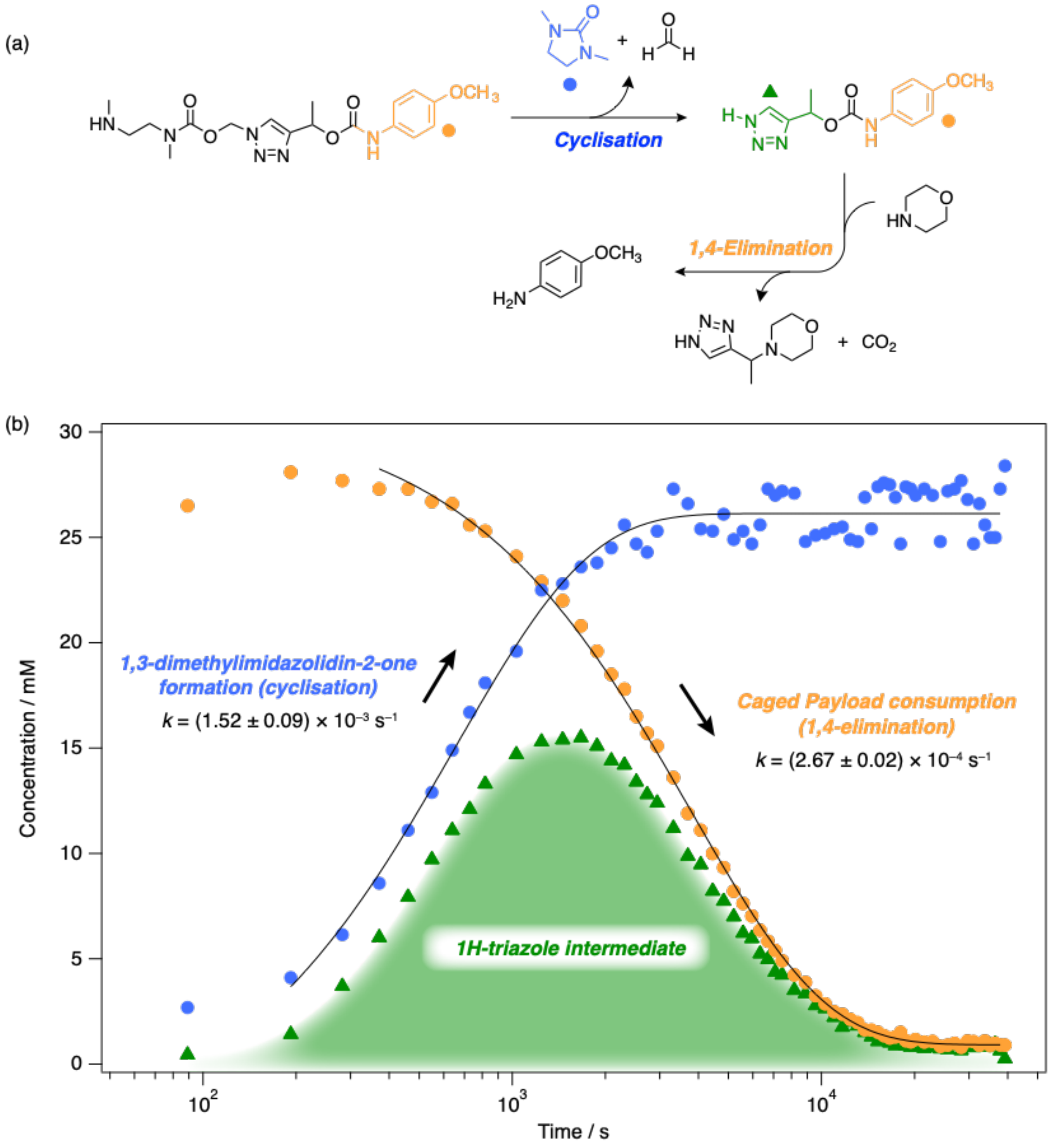

Figure S76. Overlaying the kinetics data shows that the 1*H*-triazole intermediate forms at the same rate as the cyclisation step and is consumed as the same rate as the electronic elimination step. (a) Reaction scheme illustrating the proposed self-immolation mechanism. (b) Time-speciation diagram showing how the presence of the 1*H*-triazole intermediate corresponds with the cyclisation and electronic elimination steps.

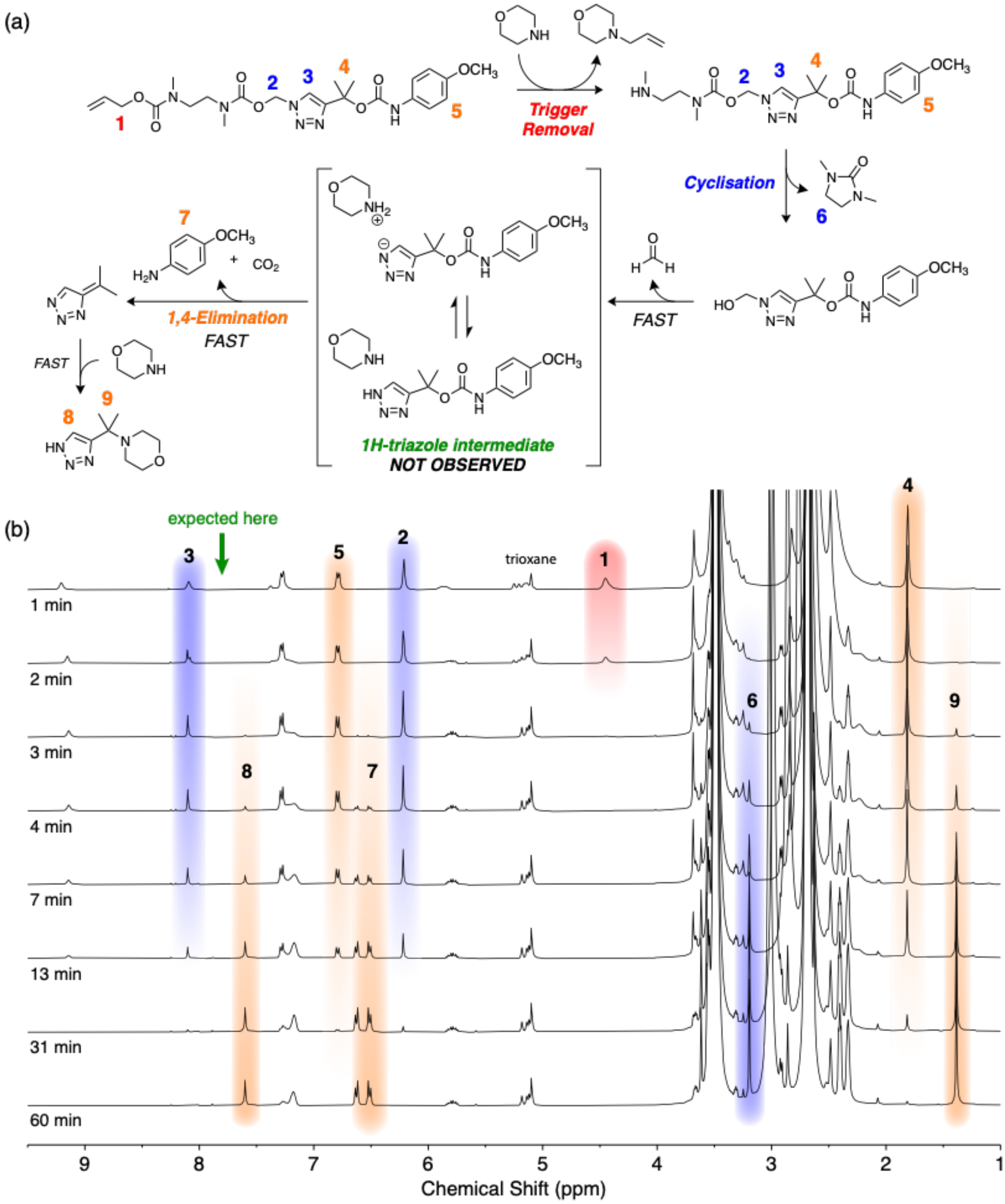

Figure S77. NMR kinetics for self-immolation of model **1c** in DMSO-d_6. (a) Reaction scheme showing key reactive species according to the proposed self-immolation mechanism. Numbered ^{1}H assignments are color-coded to match the peaks in spectra. (b) Stacked ^{1}H NMR spectra (400 MHz, DMSO-d_6, 333 K) showing a representative cross-section of the spectra recorded to construct the kinetics traces. Note that the 1*H*-triazole intermediate was not observed during the reaction, which we attribute to similar rates of the cyclisation and 1,4-elimination steps.

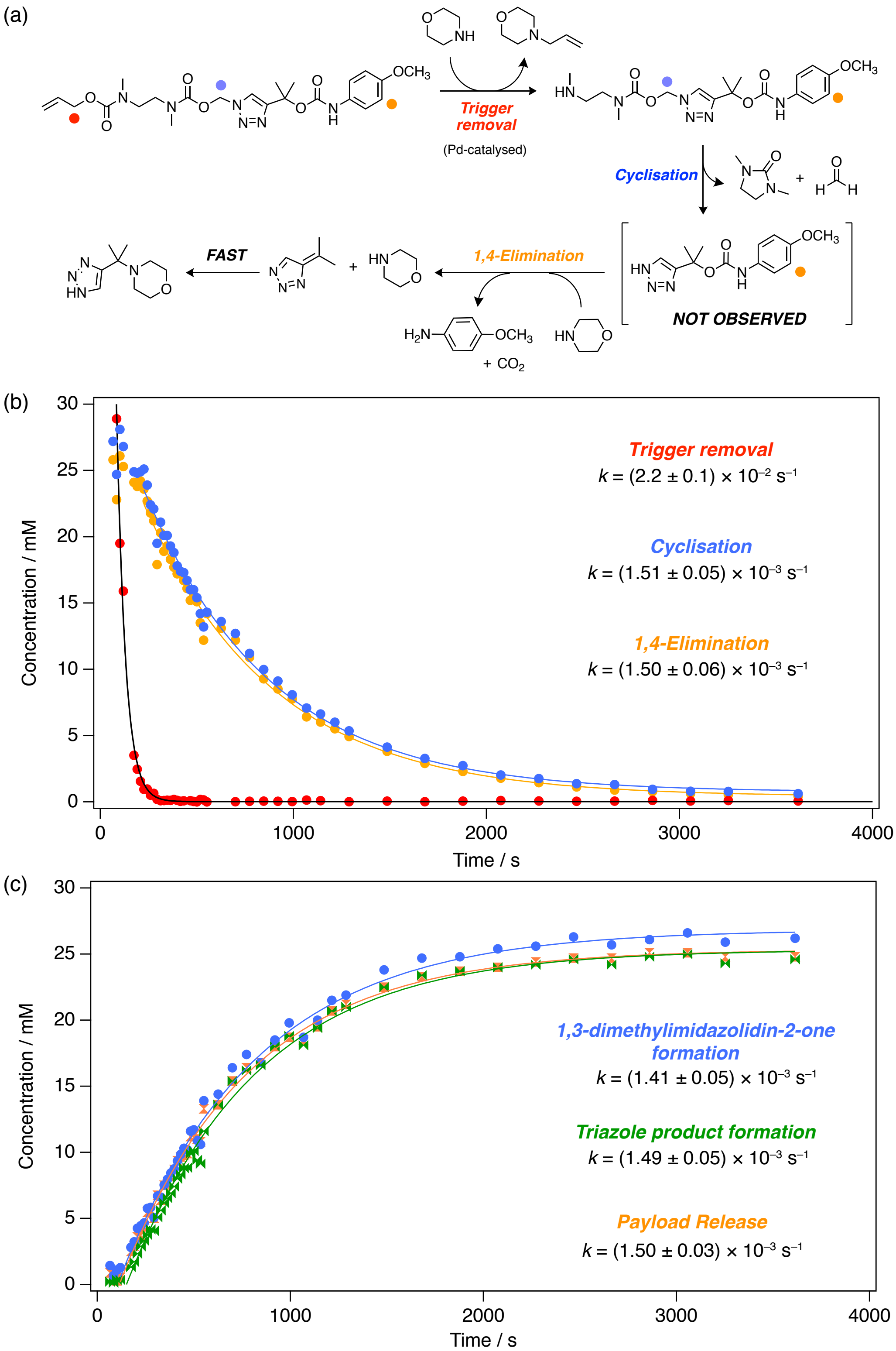

Figure S78. (a) Self-immolation mechanism of model **1c**. Colored shapes denote ^{1}H nuclei monitored to obtain kinetics traces in (b) and (c). **(b)** NMR kinetics traces (DMSO-d_6, 333 K) showing the time-dependent product distribution during self-immolation of model **1c**. Black lines represent monoexponential fits according to the pseudo-first-order model described in Equation S4. The traces clearly highlight that 1,4-elimination was sufficiently rapid for its rate to compete with the cyclisation. The fast rate of 1,4-elimination is attributed to the high stability of the triazafulvene intermediate formed from 1,4-elimination across the triazole ring. (c) Overlaid kinetics traces highlighting that the cyclisation and 1,4-elimination products formed at the same rate. Identical rates of formation of triazole product **6c** and anisidine confirm rapid Michael addition to triazafulvene **5c** in the presence of excess morpholine.

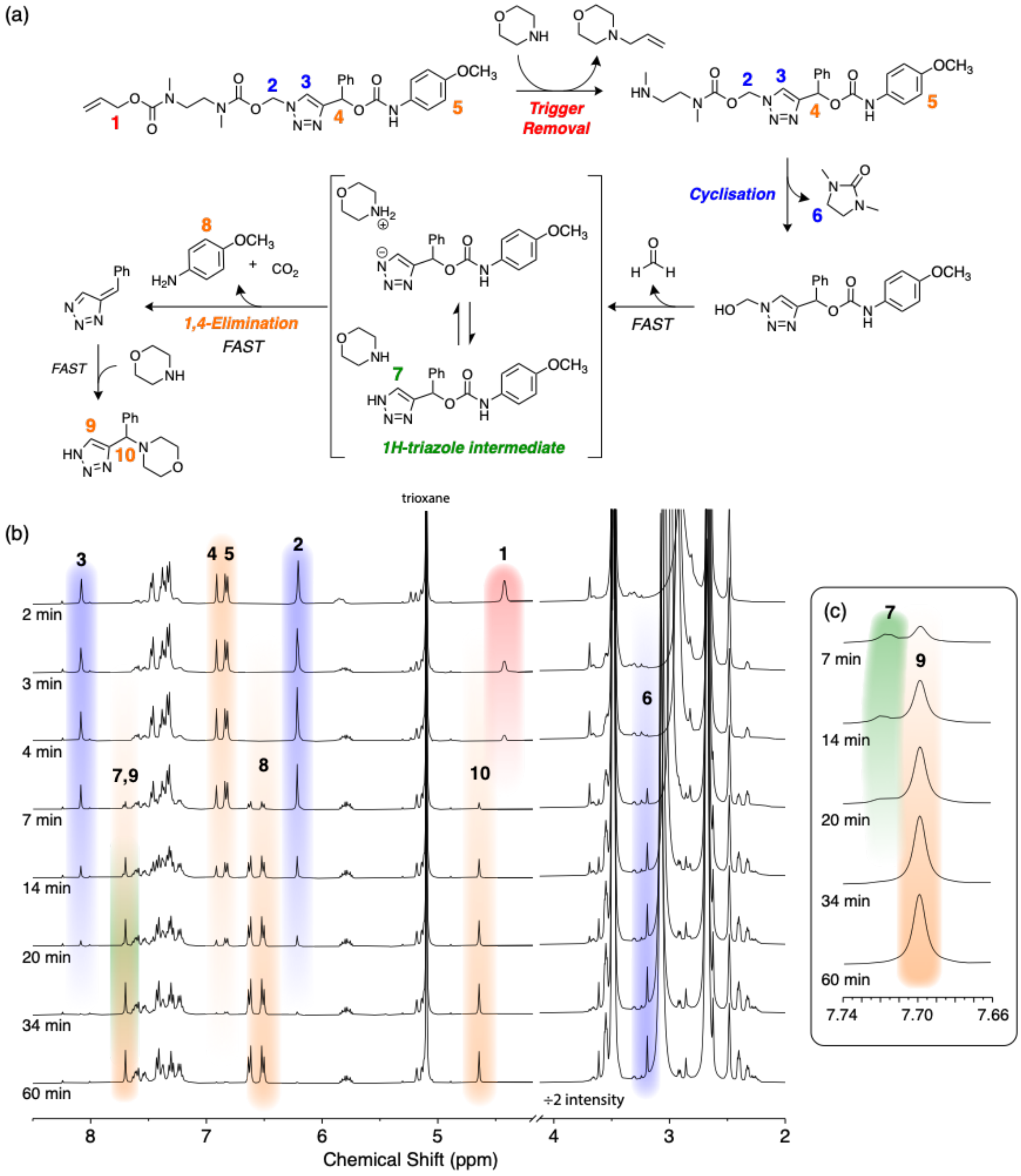

Figure S79. NMR kinetics for self-immolation of model **1d** in DMSO-d_6. **(a)** Reaction scheme showing key reactive species according to the proposed self-immolation mechanism. Numbered ^{1}H assignments are color-coded to match the peaks in the stacked spectra. **(b)** Stacked ^{1}H NMR spectra (400 MHz, DMSO-d_6, 333 K) showing a representative cross-section of the spectra recorded to construct the kinetics traces.

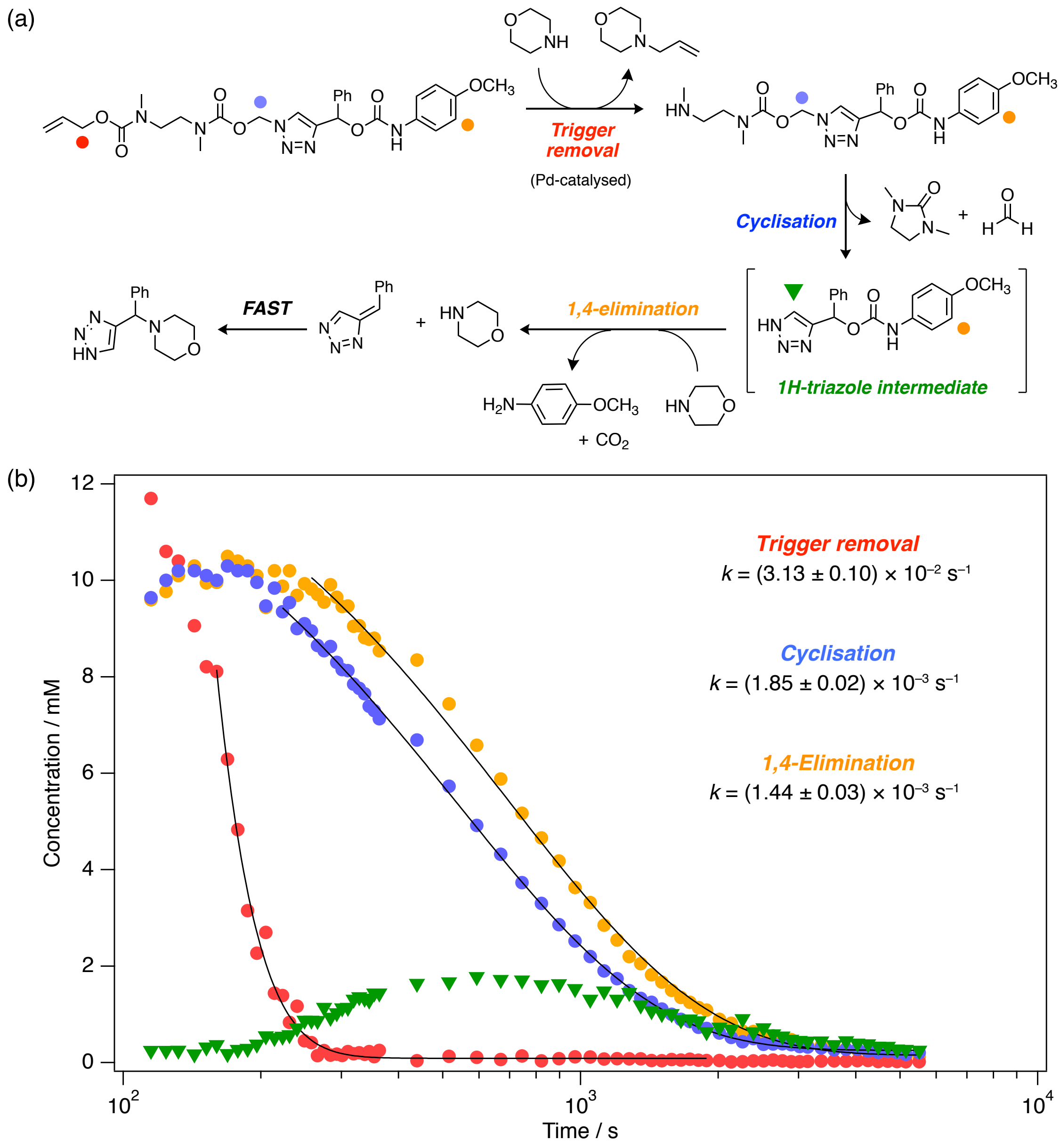

Figure S80. (a) Self-immolation mechanism of model **1d**. Colored circles denote ^{1}H nuclei monitored to obtain kinetics traces in (b). **(b)** NMR kinetics traces (DMSO-d_6, 333 K) showing the time-dependent product distribution during self-immolation of model **1d**. Black lines represent monoexponential fits according to the pseudo-first-order model described in Equation S4. The traces clearly highlight close competition between cyclisation and electronic elimination processes, which is attributed to the stability of triazafulvene intermediate **5d**.

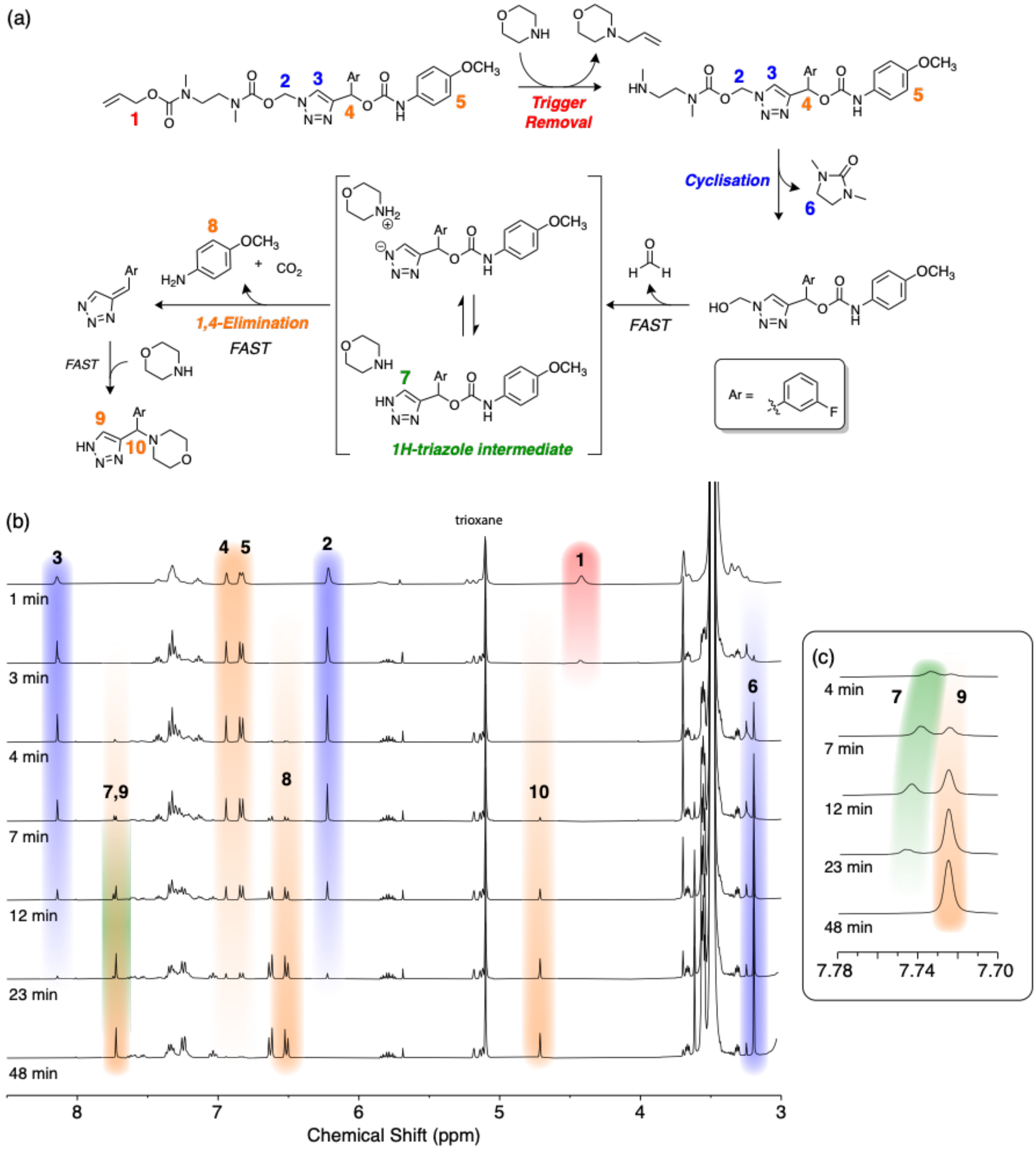

Figure S81. NMR kinetics for self-immolation of model **1e** in DMSO-d_6. **(a)** Reaction scheme showing key reactive species according to the proposed self-immolation mechanism. Numbered ^{1}H assignments are color-coded to match the peaks in the stacked spectra. **(b)** Stacked ^{1}H NMR spectra (400 MHz, DMSO-d_6, 333 K) showing a representative cross-section of the spectra recorded to construct the kinetics traces.

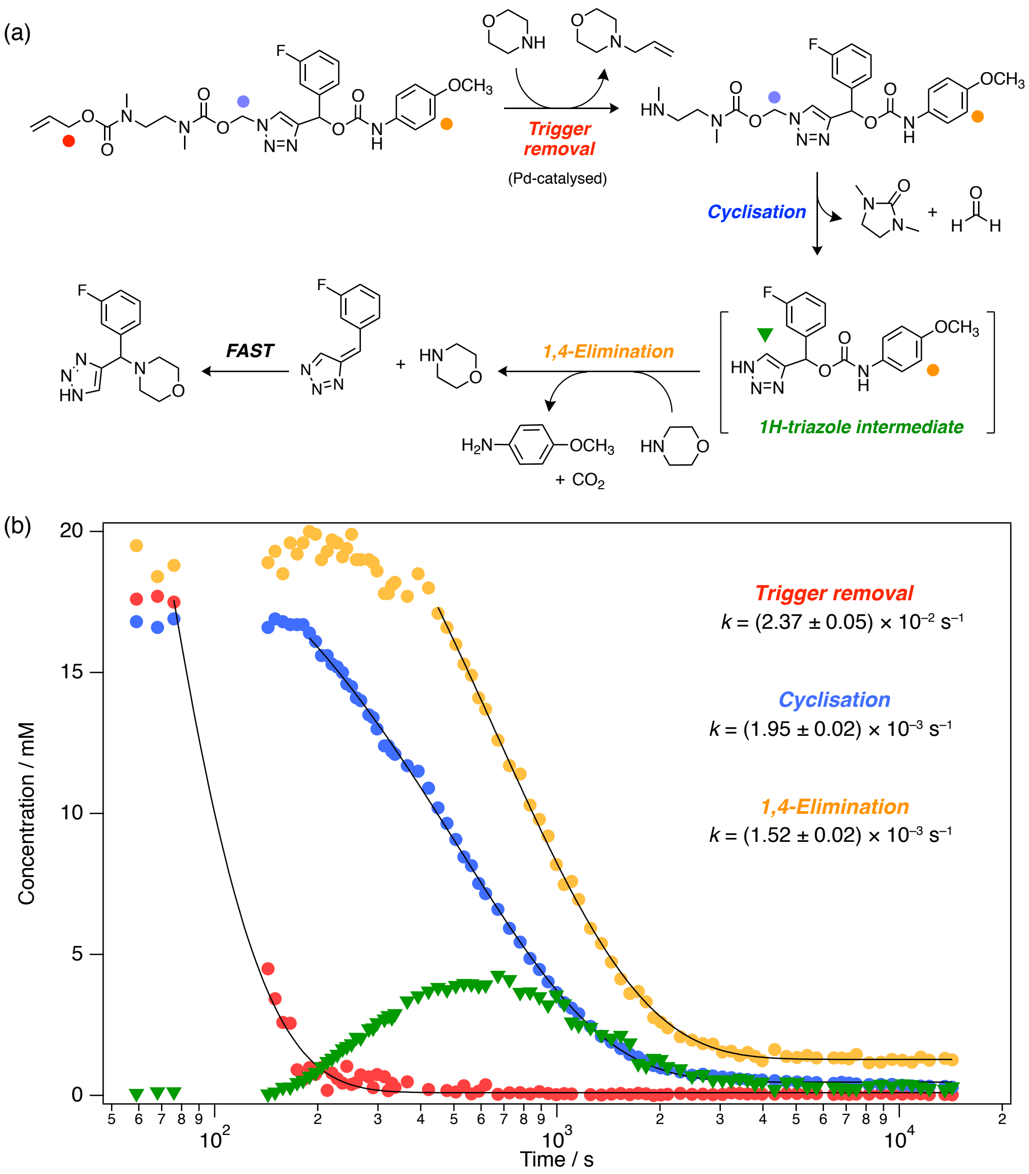

Figure S82. (a) Self-immolation mechanism of model **1e**. Colored circles denote ^{1}H nuclei monitored to obtain kinetics traces in (b). **(b)** NMR kinetics traces (DMSO-d_6, 333 K) showing the time-dependent product distribution during self-immolation of model **1e**. Black lines represent monoexponential fits according to the pseudo-first-order model described in Equation S4.

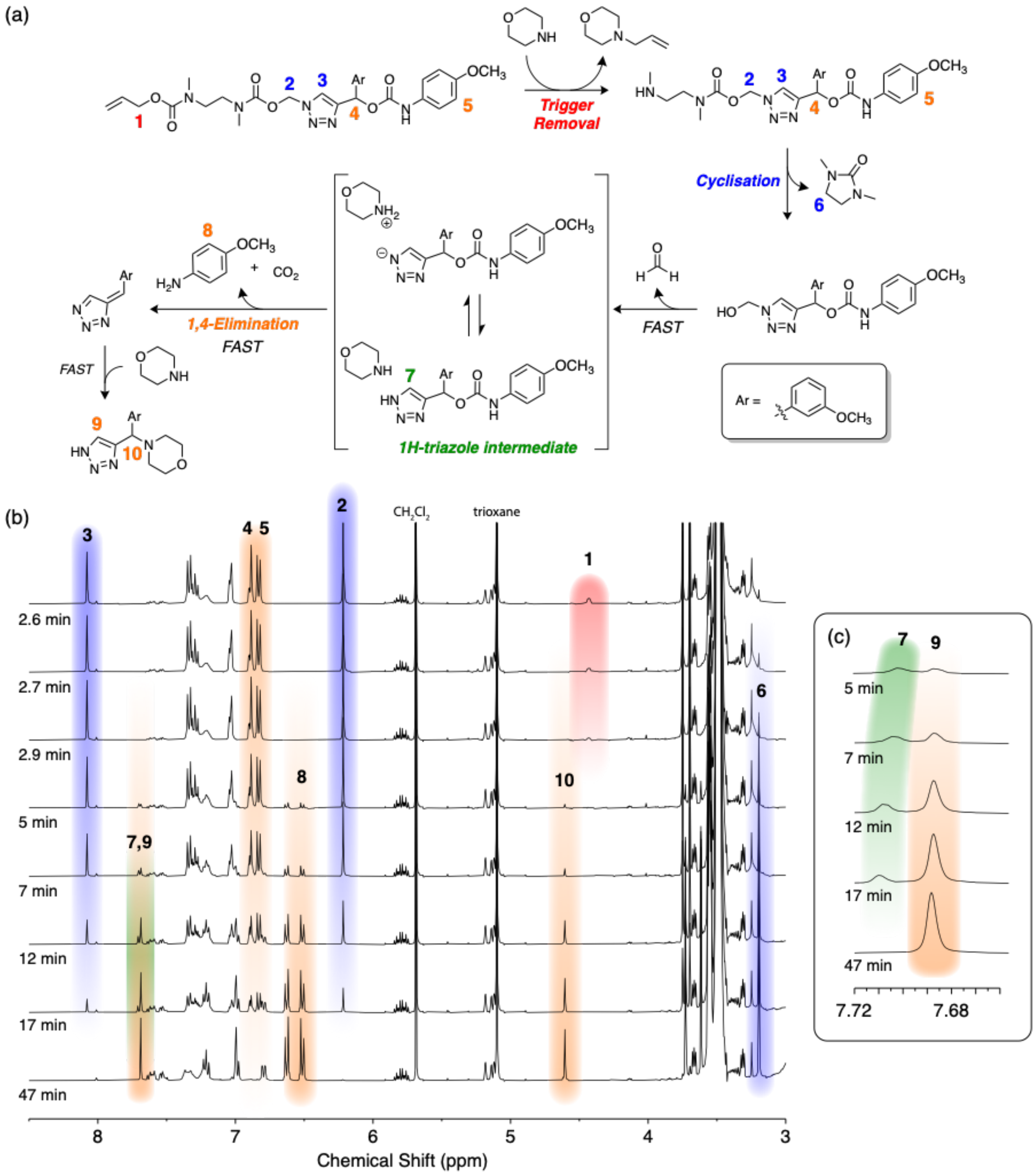

Figure S83. NMR kinetics for self-immolation of model **1f** in DMSO-d_6. **(a)** Reaction scheme showing key reactive species according to the proposed self-immolation mechanism. Numbered ^{1}H assignments are color-coded to match the peaks in the stacked spectra. **(b)** Stacked ^{1}H NMR spectra (400 MHz, DMSO-d_6, 333 K) showing a representative cross-section of the spectra recorded to construct the kinetics traces.

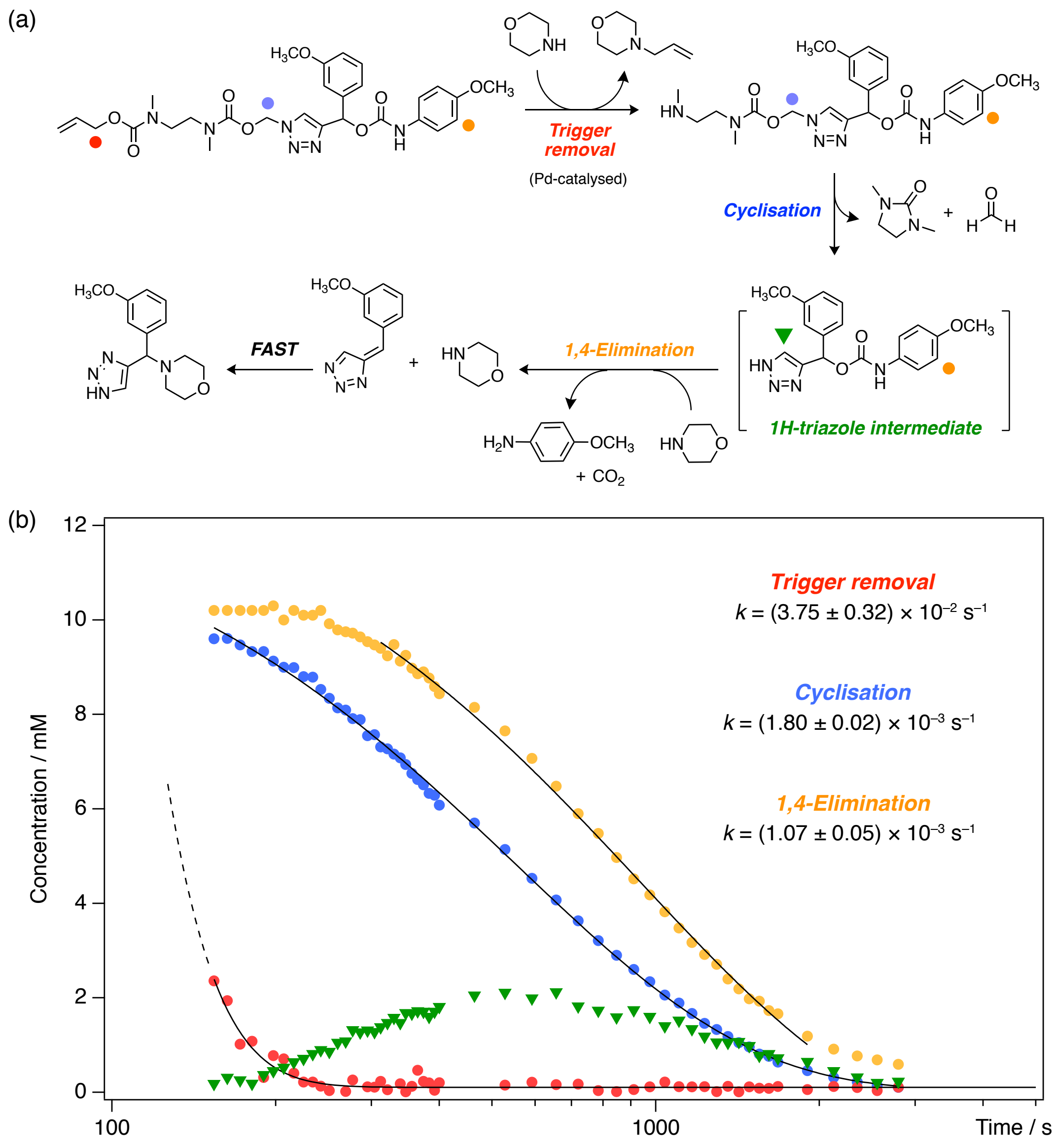

Figure S84. (a) Self-immolation mechanism of model **1f**. Colored circles denote ^{1}H nuclei monitored to obtain kinetics traces in (b). **(b)** NMR kinetics traces (DMSO-d_6, 333 K) showing the time-dependent product distribution during self-immolation of model **1f**. Black lines represent monoexponential fits according to the pseudo-first-order model described in Equation S4.

S7. Self-Immolation Kinetics of Models 1a, 1b and 1d in DMSO-d_6/D_2O

Self-immolation kinetics experiments were performed in DMSO-d_6/D_2O (8:2 *v*/*v*) to demonstrate that the cascade could also operate in aqueous solvent mixtures. Poor solubility of the model compounds in water limited our studies to the above mixed solvent system; however more water-soluble self-immolative linkers are the focus of current work. Control and kinetics data for **1a**, **1b** and **1d** in DMSO-d_6/D_2O are shown in Sections S7.1 and S7.2 to study across all three kinetics regimes (days, hours and minutes) examined in this work. Pausing experiments were not performed for **1c**, **1e** and **1f** due to their similar cascade behavior to **1d** (which served as a representative model system) and to reduce spectrometer/reagent costs. Control experiments for all three model compounds revealed the excellent stability of all three compounds in DMSO-d_6/D_2O (8:2 *v*/*v*) containing excess morpholine (50 equiv.) (Figure Figure **S85**–Figure **S87**). In each case, no hydrolysis was observed during the timescales investigated.

S7.1. Self-immolation control data for 1a, 1b and 1d in DMSO-d_6/D_2O

General control experiment procedure:

A 5 mm NMR tube was charged with a DMSO solution of the model compound (~14 µmol, 84 mM), morpholine (62 µL, 714 µmol, 50 equiv.), D_2O containing *t*BuOH (~100 µL, 15.6 mM) and DMSO-d_6 (made up to a total volume of ~550-600 µL). The tube was inserted into the spectrometer, equilibrated at 60 °C (~5 min) then an initial spectrum was collected. For models **1b** and **1d**, tubes were maintained at 60 °C inside the spectrometer; for **1a**, the sample was ejected, and the tube incubated at 60 °C in a glycerol bath before recording the end timepoint.

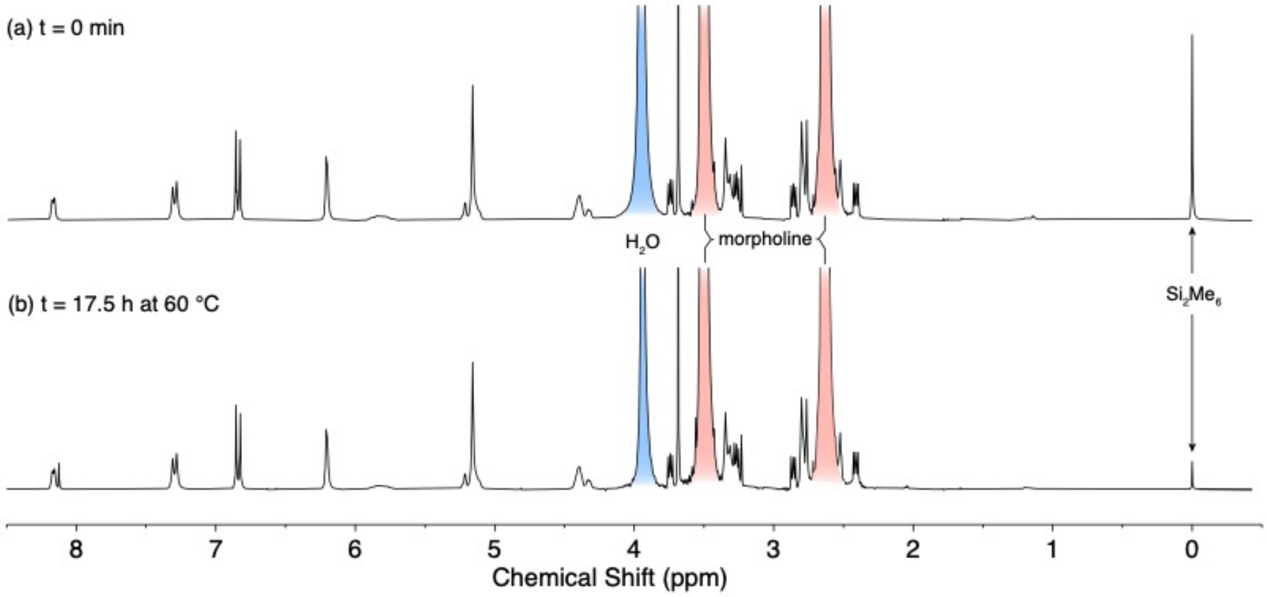

Figure S85. ^{1}H NMR kinetics control experiment (300 MHz, DMSO-d_6/D_2O 8/2 *v*/*v*, 300 K) of model **1a** with morpholine (a) before and (b) after heating at 60 °C for 17.4 h. No observable hydrolysis or degradation was observed, revealing the stability of the untriggered model compound in the presence of water on the timescale of **1a**'s self-immolation cascade, which proceeds to completion within ~18 h at 60 °C. Note, also, that the hexamethydisilane (Si_2Me_6) standard was observed to evaporate over this period, which led us to use *t*BuOH as the internal standard for kinetics experiments in organic/aqueous mixtures.

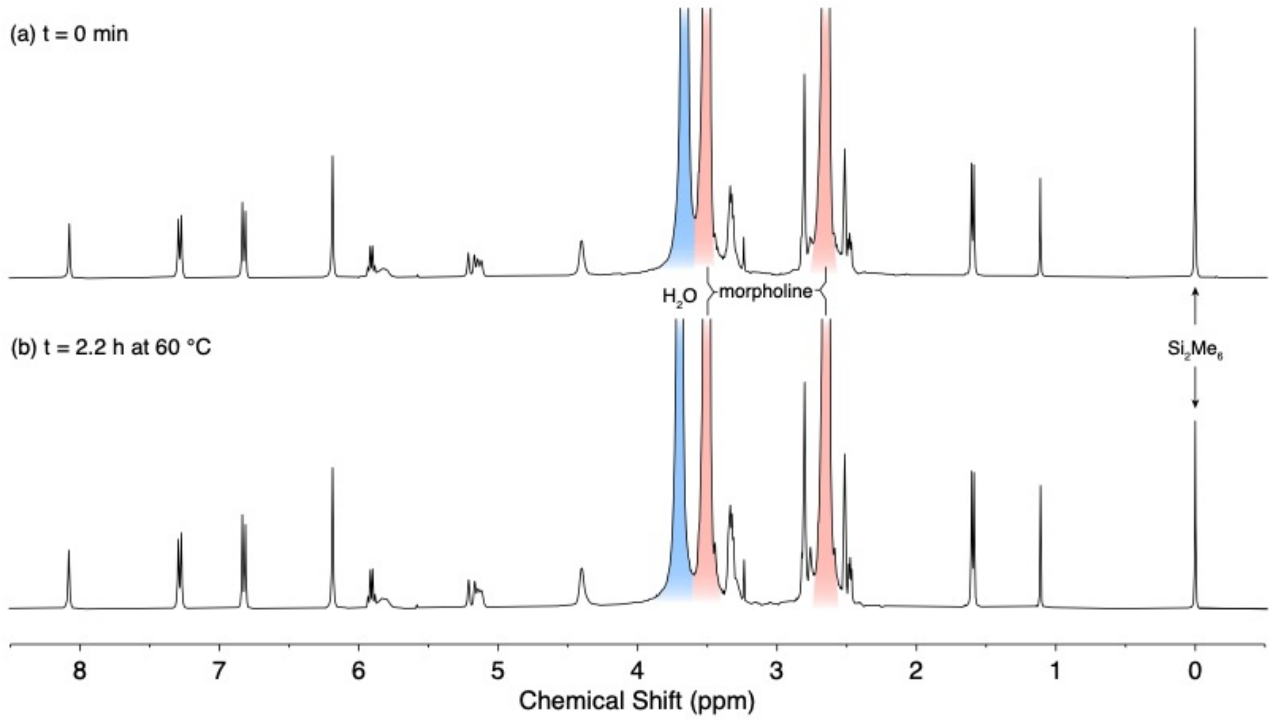

Figure S86. ^{1}H NMR kinetics control experiment (400 MHz, DMSO-d_6/D_2O 8/2 *v*/*v*, 333 K) of model **1b** with morpholine (a) before and (b) after heating at 60 °C for 2.2 h. No observable hydrolysis or degradation was observed, revealing the stability of the untriggered model compound in the presence of water on the timescale of **1b**'s self-immolation cascade, which proceeds to completion within ~20 min at 60 °C.

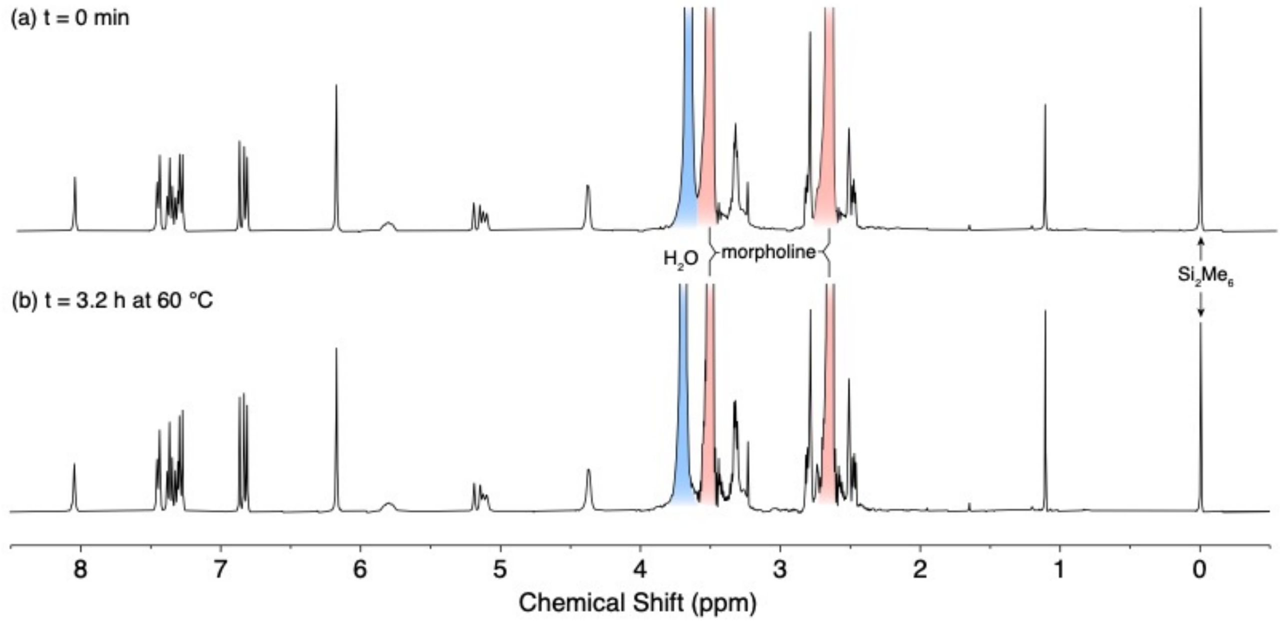

Figure S87. ^{1}H NMR kinetics control experiment (400 MHz, DMSO-d_6/D_2O 8/2 *v*/*v*, 333 K) of model **1d** with morpholine (a) before and (b) after heating at 60 °C for 3.2 h. No observable hydrolysis or degradation was observed, revealing the stability of the untriggered model compound in the presence of water on the timescale of **1d**'s self-immolation cascade, which proceeds to completion within ~10 min at 60 °C.

S7.2. Self-immolation kinetics data for 1a, 1b and 1d in DMSO-d_6/D_2O

General procedure for kinetics experiments

In a typical experiment, a 5 mm NMR tube was charged with DMSO solution of the model compound (~14 µmol, 84 mmol), morpholine (62 µmol, 714 µmol, 50 equiv.), D_2O containing *t*BuOH (~100 µL, 15.6 mM) in DMSO-d_6 (made up to a total volume of ~500-550 µL). The tube was inserted into the spectrometer, equilibrated at 60 °C (~5 min) then an initial spectrum was collected to calibrate the concentration. The tube was then ejected from the spectrometer, a suspension of $Pd(PPh_3)_4$ (~1 µmol) in DMSO-d_6 (100 µL) added and the tube mixed rapidly before returning the sample to the spectrometer. The sample was generally left out of the spectrometer for <1 min during catalyst addition and remained within 5 °C of the target temperature. The sample was re-shimmed and kinetics timepoints were collected immediately. Data (*ds* = 0, *ns* = 1, *zg30* pulse with *D1* = 2 s) were collected continuously throughout the experiment at variable time intervals by varying the number of dummy scans. Data were processed according to the procedure described in Section S6. Table S3 provides a summary of pseudo-first-order rate constants (k_{obs}) for the three stages of self-immolation for model compounds **1a**, **1b**, **1d**, and NMR spectra and kinetics plots are shown in Figures Figure **S88**–Figure **S93**.

Table S3. Summary of pseudo-first-order rate constants (k_{obs}) for the three stages of self-immolation for model compounds **1a**, **1b**, **1d** in DMSO-d_6/D_2O (8:2 *v*/*v*) at 60 °C

Compound	Trigger Removal (s^{-1})	Cyclisation (s^{-1})	1,4-elimination (s^{-1})
**1a**	–[a]	$(5.81 \pm 0.05) \times 10^{-3}$	$(8.05 \pm 0.16) \times 10^{-6}$
**1b**	$(1.06 \pm 0.04) \times 10^{-1}$	$(5.89 \pm 0.09) \times 10^{-3}$	$(2.85 \pm 0.07) \times 10^{-3}$
**1d**	$(8.7 \pm 1.1) \times 10^{-2}$	$(5.74 \pm 0.14) \times 10^{-3}$	$(5.66 \pm 0.13) \times 10^{-3}$

[a]Trigger removal was complete within the equilibration and shimming period.

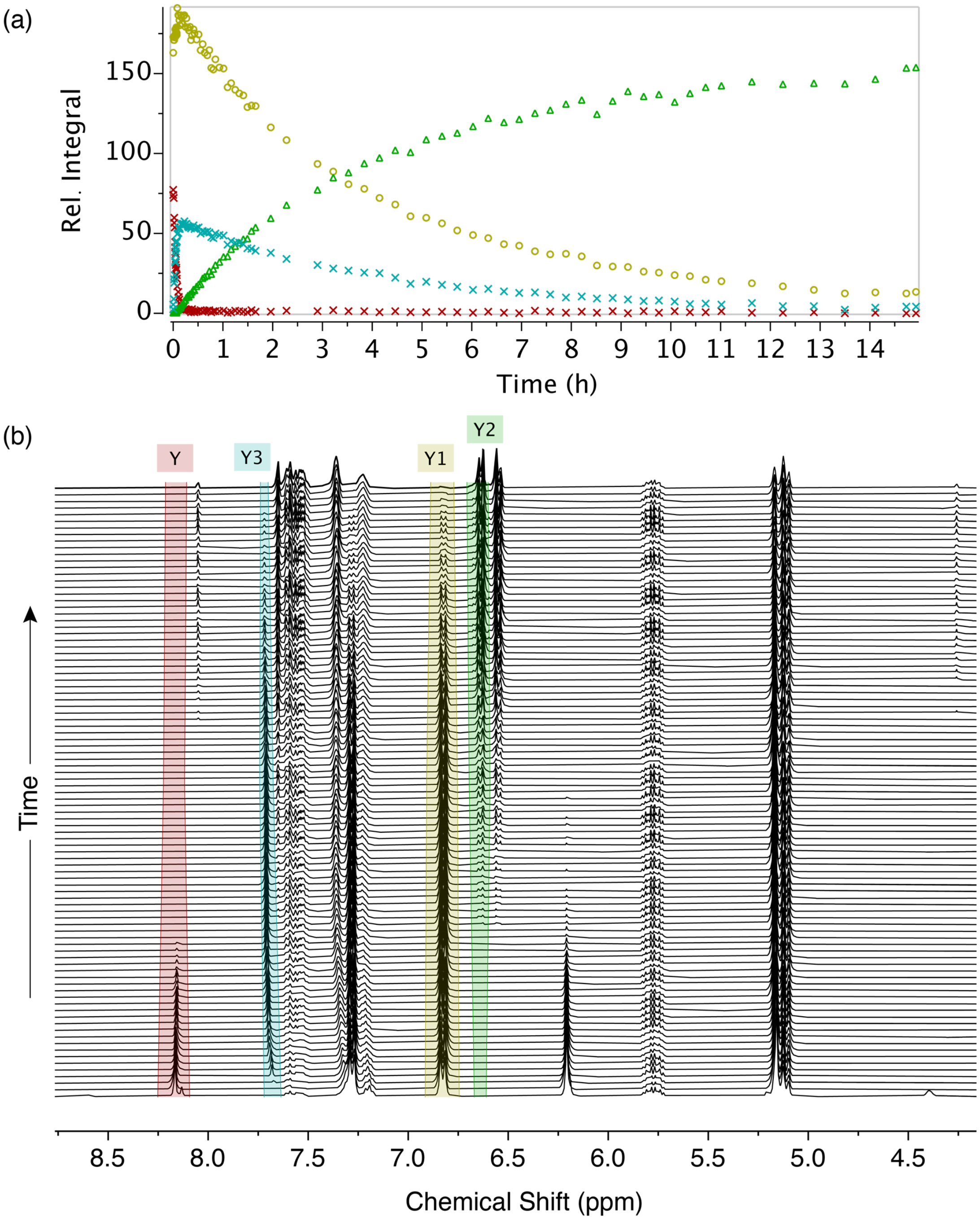

Figure S88. NMR kinetics for self-immolation of model **1a** in DMSO-d_6/D_2O 8:2 *v*/*v* at 60 °C. **(a)** (a) Relative integral plotted against time. **(b)** Stacked ^1H NMR spectra (partial) showing integrated regions for kinetics analysis (t_0 shown at bottom).

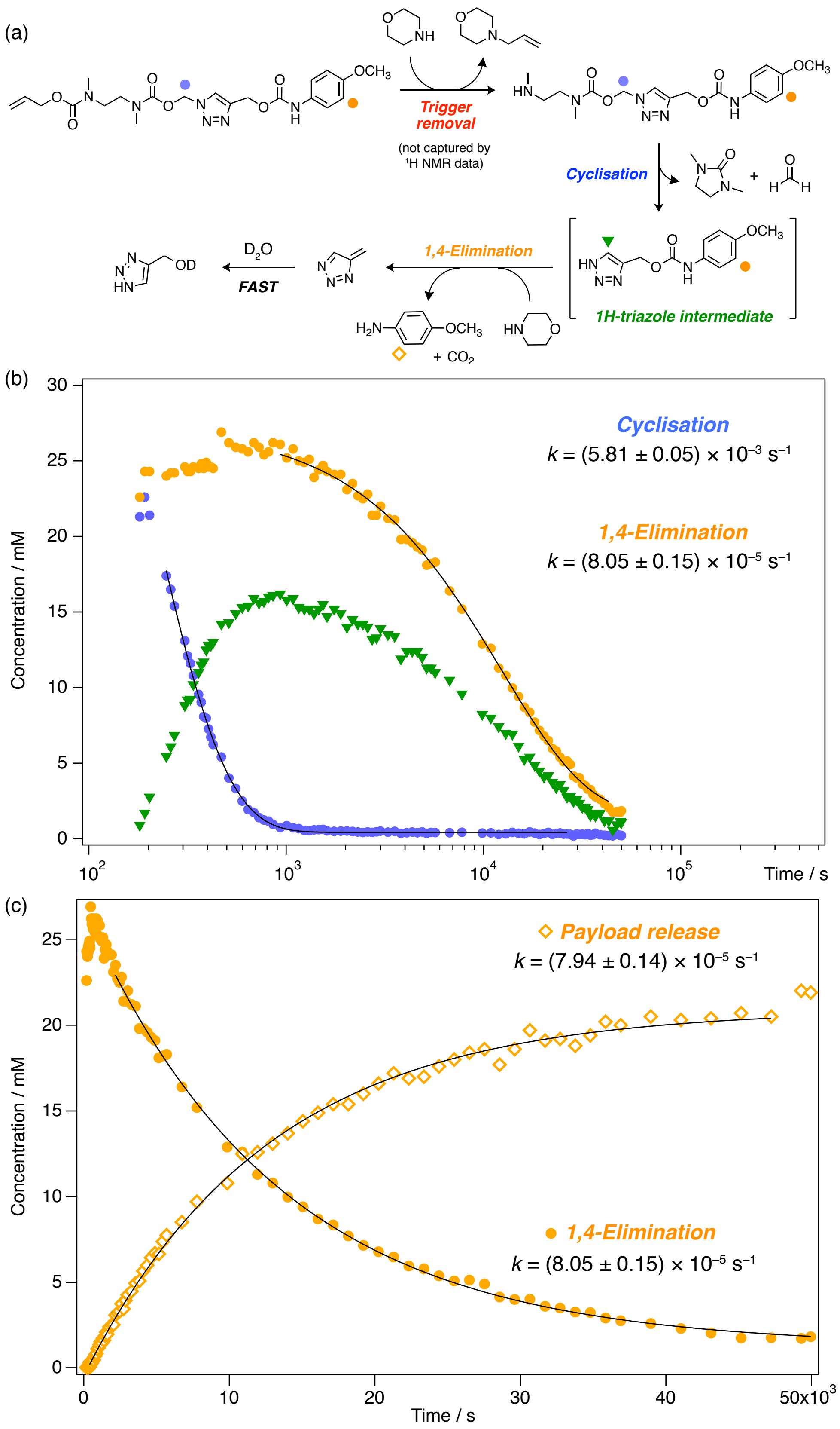

Figure S89. (a) Proposed self-immolation mechanism for model **1a** in DMSO-d_6/D_2O. Colored markers denote ^{1}H nuclei monitored to obtain kinetics traces in (b) and (c). **(b)** NMR kinetics traces (DMSO-d_6/D_2O 8/2 *v*/*v*, 333 K) showing the time-dependent product distribution during self-immolation of model **1a.** Black lines represent monoexponential fits according to the pseudo-first-order model described in Equation S4. **(c)** Kinetics traces comparing the rates of 1,4-elimination (product loss) and payload release.

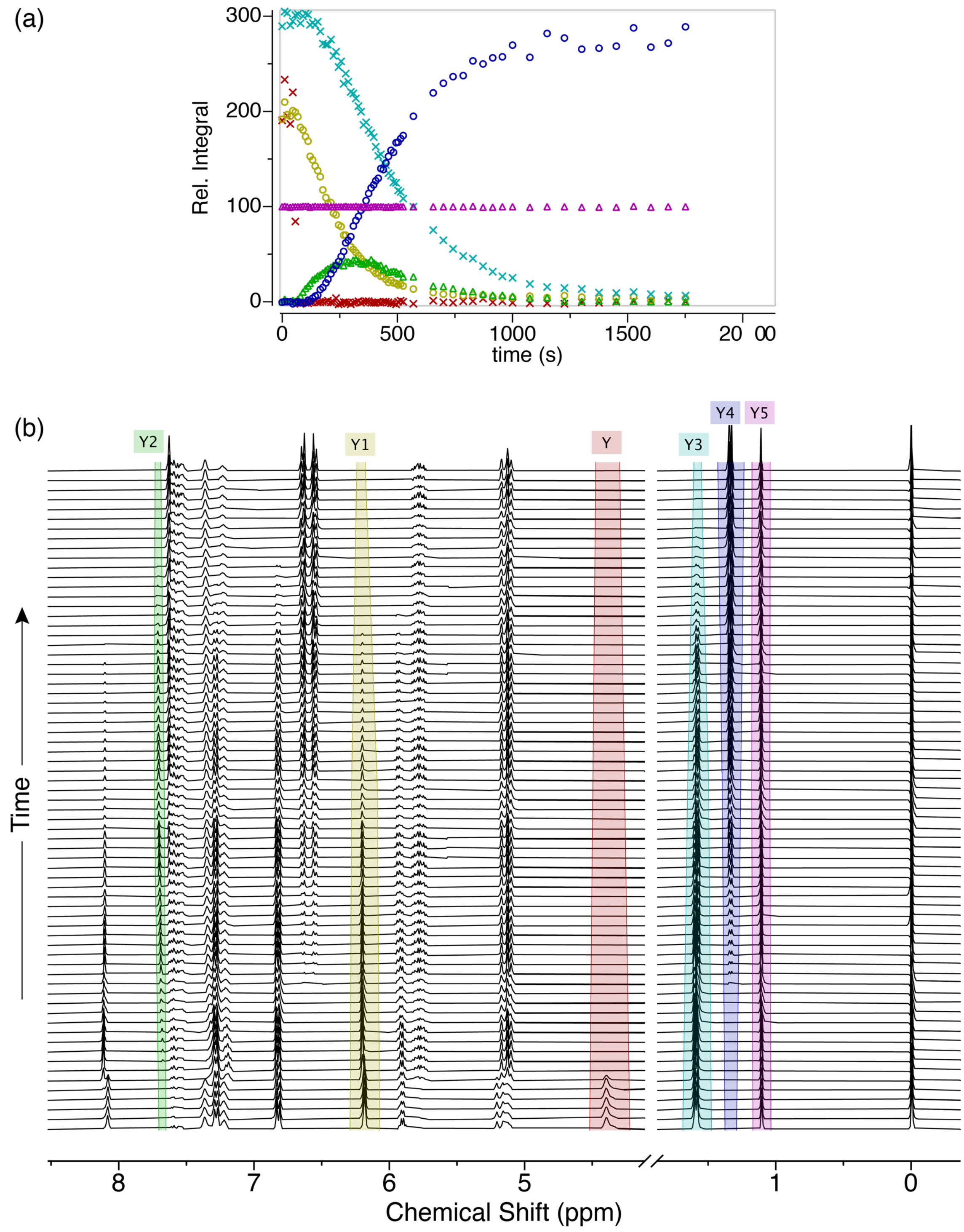

Figure S90. NMR kinetics for self-immolation of model **1b** in DMSO-d_6/D_2O 8:2 *v*/*v* at 60 °C. **(a)** Relative integral plotted against time. **(b)** Stacked ^{1}H NMR spectra (partial) showing integrated regions for kinetics analysis (t_0 shown at bottom).

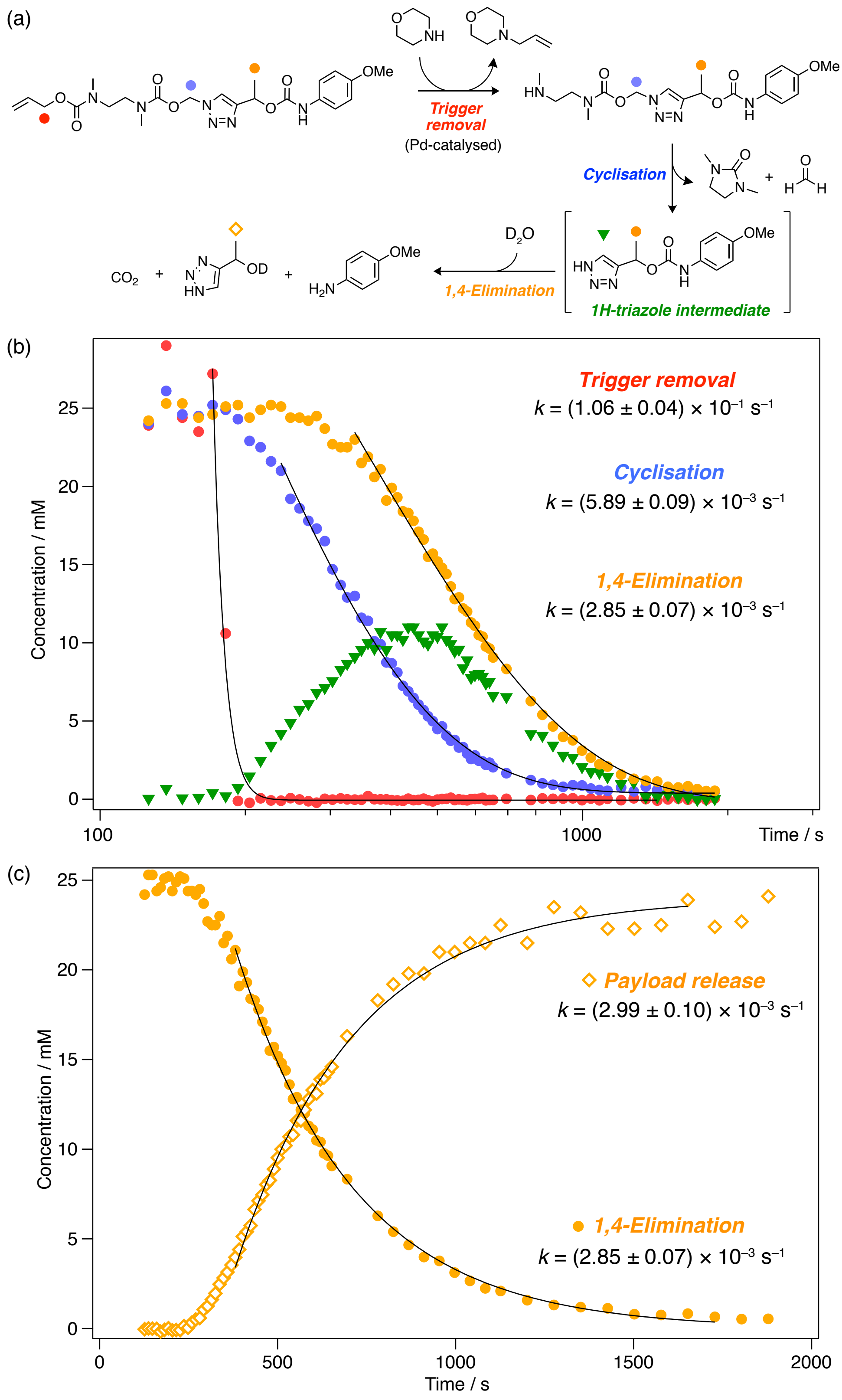

Figure S91. (a) Proposed self-immolation mechanism for model **1b** in DMSO-d_6/D_2O. Colored markers denote ^{1}H nuclei monitored to obtain kinetics traces in (b) and (c). **(b)** NMR kinetics traces (DMSO-d_6/D_2O 8/2 *v*/*v*, 333 K) showing the time-dependent product distribution during self-immolation of model **1b.** Black lines represent monoexponential fits according to the pseudo-first-order model described in Equation S4. **(c)** Kinetics traces comparing the rates of 1,4-elimination (product loss) and payload release.

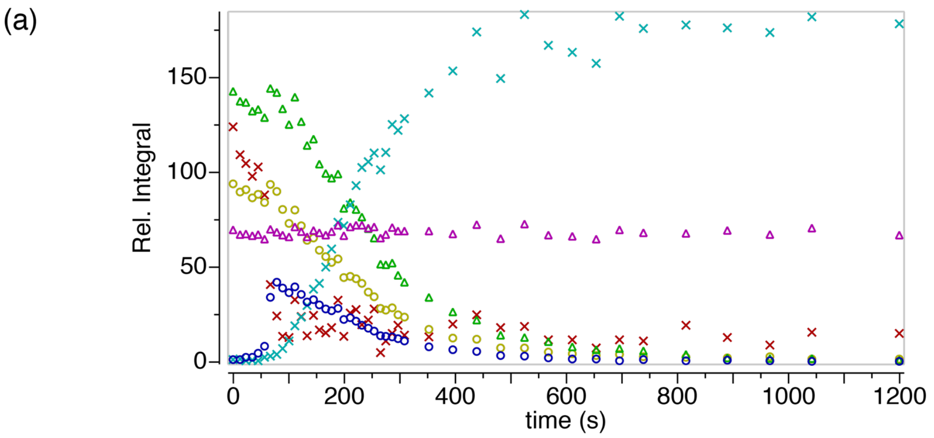

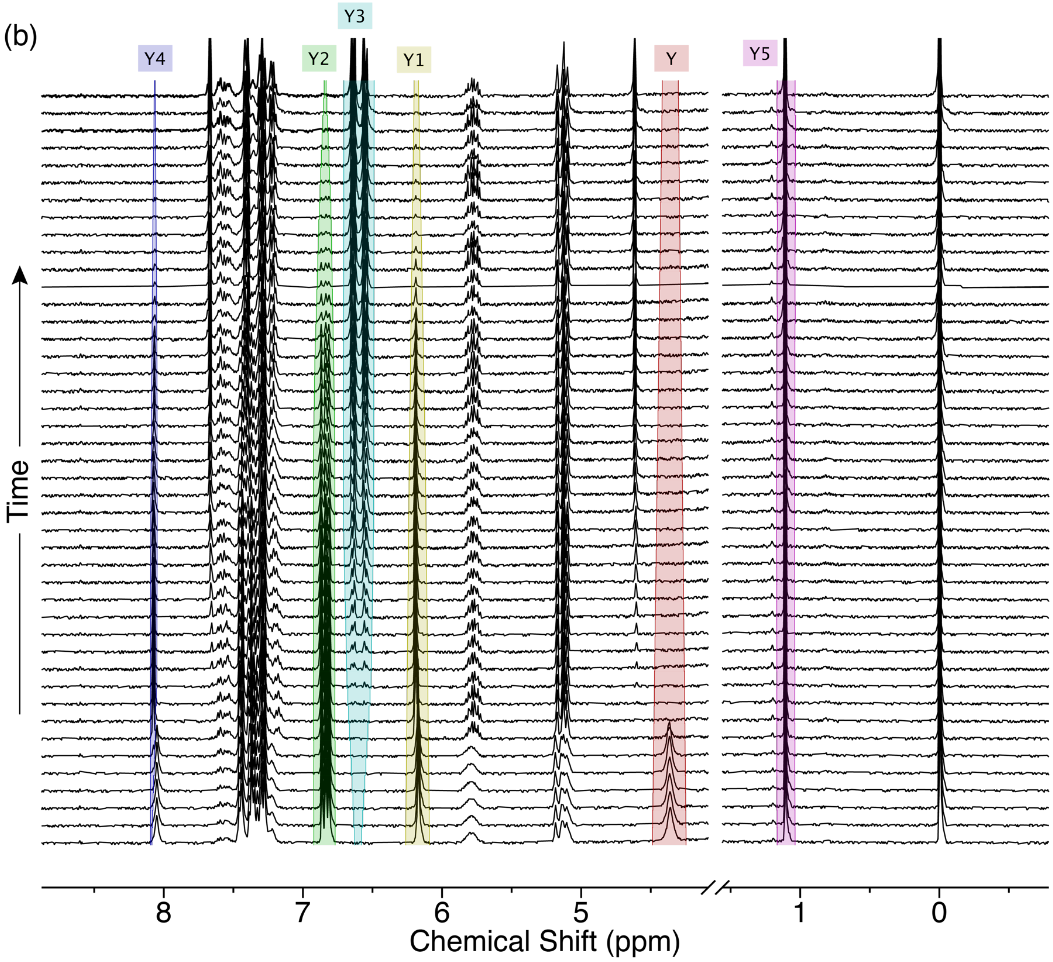

Figure S92. NMR kinetics for self-immolation of model **1d** in DMSO-d_6/D_2O 8:2 *v*/*v* at 60 °C. **(a)** Relative integral plotted against time. **(b)** Stacked ^1H NMR spectra (partial) showing integrated regions for kinetics analysis (t_0 shown at bottom).

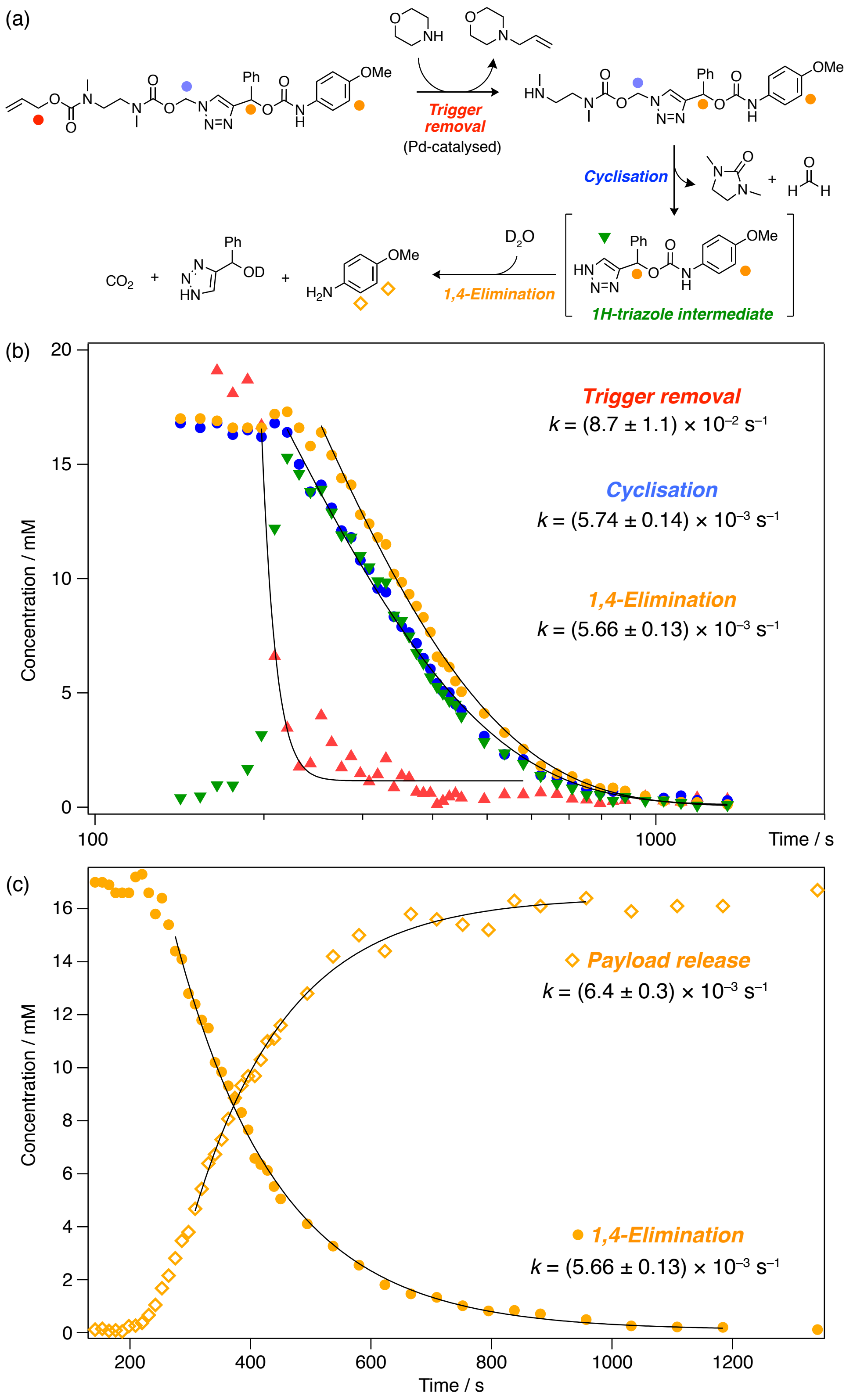

Figure S93. (a) Proposed self-immolation mechanism for model **1b** in DMSO-d_6/D_2O. Colored markers denote ^{1}H nuclei monitored to obtain kinetics traces in (b) and (c). **(b)** NMR kinetics traces (DMSO-d_6/D_2O 8/2 *v*/*v*, 333 K) showing the time-dependent product distribution during self-immolation of model **1b.** Black lines represent monoexponential fits according to the pseudo-first-order model described in Equation S4. The fast rate of trigger removal resulted in poor fitting to the decay model; therefore, the measured rate constant is an indicative value only. **(c)** Kinetics traces comparing the rates of 1,4-elimination (product loss) and payload release.

S8. Acid/base-mediated switching in DMSO-d_6 (Models 1a, 1b and 1e)

General Procedure

A standard 5 mm NMR tube was charged with a solution of model compound (~16 µmol, 1.0 equiv.), morpholine (70 µL, 0.82 mmol, 50 equiv.) and an internal concentration standard (1,3,5-trioxane or hexamethyldisilane, typically ~3 mM total concentration) in DMSO-d_6 (552 µL). The tube was inserted into the NMR spectrometer, equilibrated at 60 °C until stable (~5 min) then the spectrometer was tuned and matched, locked and shimmed. An initial spectrum was collected for concentration calibration (typically *ds* = 2, *ns* = 8, running *zg30* pulse with a *D1* recycle delay of 2 s to ensure complete longitudinal relaxation of the sample between each scan to enable quantitative integration of the spectrum). The tube was ejected from the spectrometer, a suspension of $Pd(PPh_3)_4$ (1.3 mg, 1.2 µmol) in DMSO-d_6 (100 µL) added and the tube mixed rapidly before returning the sample to the spectrometer (sample was outside spectrometer for <1 min). Upon returning to the spectrometer, the sample was re-shimmed and kinetics timepoints were collected immediately. The frequency at which spectra were collected was controlled by varying the number of dummy scans.

To pause the self-immolation cascade for the first time, the NMR tube was ejected from the spectrometer, an aliquot of trifluoroacetic acid (6.0 µL, 82 µmol, 5.0 equiv.) added, the tube inverted rapidly to mix then returned to the spectrometer within 1 min. The sample was re-shimmed and kinetics timepoints collected immediately. After 45 min, three sequential attempts were made to reactivate the cascade by adding Cs_2CO_3 (11 mg, 33 µmol, 2.0 equiv.) and mixing the sample then re-inserting the sample and checking the kinetics trajectory for 10 min, then adding a second lot of Cs_2CO_3 (11 mg, 33 µmol, 2.0 equiv.) and checking the kinetics trajectory for a further 10 min. Upon addition of a third lot of Cs_2CO_3 (11 mg, 33 µmol, 2.0 equiv.), the cascade was observed to restart and was allowed to proceed for a further 30 min before adding a second aliquot for trifluoroacetic acid (10 µL, 0.13 mmol, 8.0 equiv.). After 55 min in the dormant state, Cs_2CO_3 (50 mg, 0.16 mmol, 9.5 equiv.) was added to reactivate the cascade. The reaction was allowed to proceed to completion.

Due to the long duration of model **1a**'s self-immolation cascade, the tube was heated in a glycerol bath at 60 °C in between NMR measurements. When recording a measurement (performed at a probe temperature of 300 K), the tube would be out of the heating bath for no longer than 10 min at a time, which is negligible on the overall timescale of the experiment.

Data for the acid/base-mediated switching of models **1a**, **1b** and **1e** in DMSO-d_6 are shown in Figures Figure **S94**–Figure **S98** to demonstrate that the switching mechanism operates under all three kinetics regimes (days, hours and minutes) examined in this work. Pausing experiments were not performed for **1c**, **1d** and **1f** due to their very similar cascade behavior to **1e** (which served as a representative model system for the fast kinetics regime).

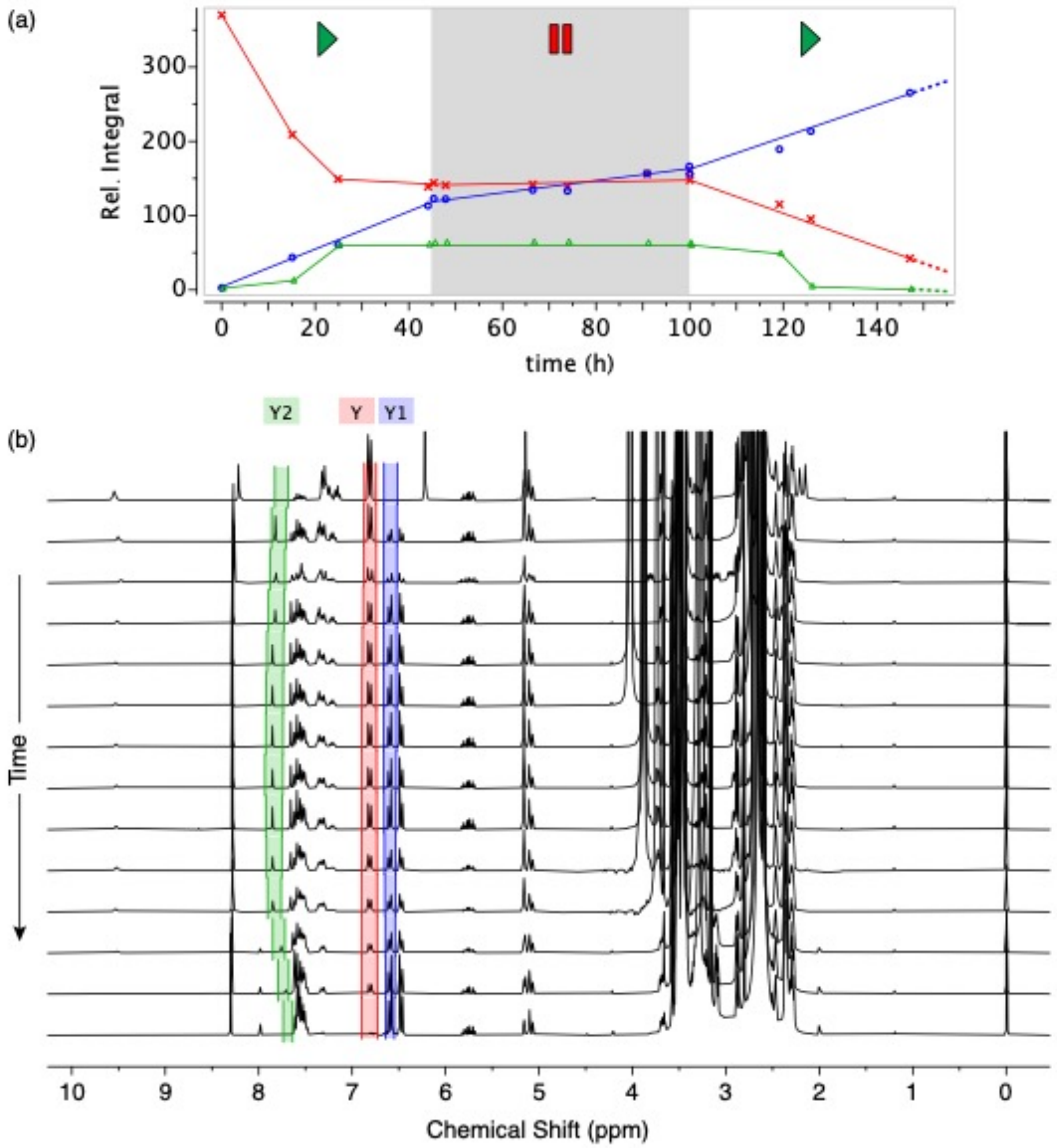

Figure S94. ^{1}H NMR acid/base-mediated switching experiment using model **1a** (300 MHz, DMSO-d_6, 333 K). Note that inconsistent spacing of the datapoints reflected the booking availability of the NMR spectrometer during the course of this experiment. **(a)** Relative integral plotted against time showing the payload phenylene resonances of the starting model (red), free anisidine (blue) and the 1*H*-triazole intermediate (green). The active and paused phases are denoted with pause and play icons, with the paused phase shaded in grey. **(b)** Stacked NMR spectra showing integrated regions, color-coded to match the trace in (a). Integrals have been normalized to the hexamethyldisilane internal standard (t_0 shown at top).

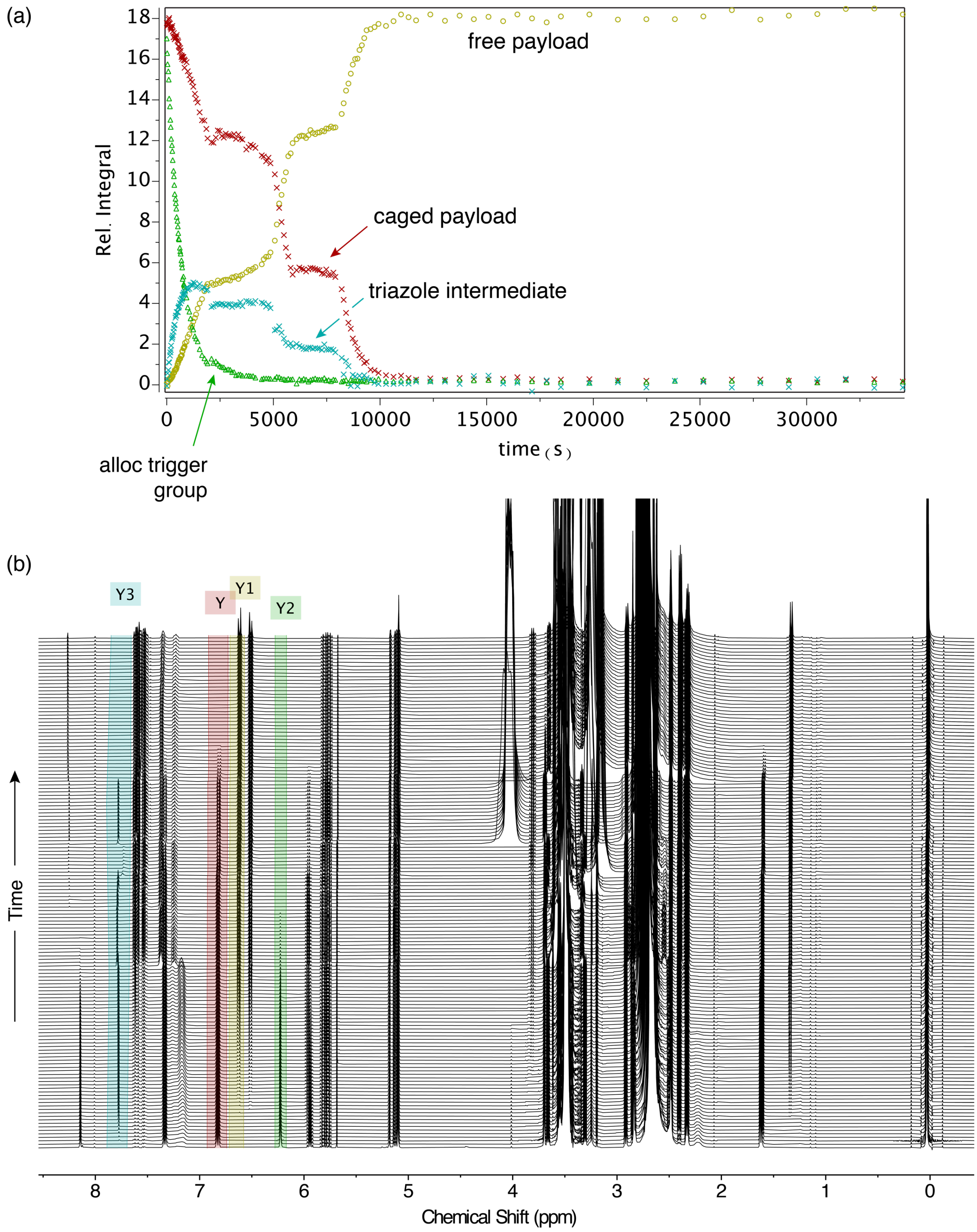

Figure S95. ^{1}H NMR acid/base-mediated switching experiment using model **1b** (400 MHz, DMSO-d_6, 333 K). **(a)** Relative integral plotted against time. **(b)** Stacked NMR spectra showing integrated regions, color-coded to match the trace in (a). Integrals have been normalized to the hexamethyldisilane internal standard (t_0 shown at bottom).

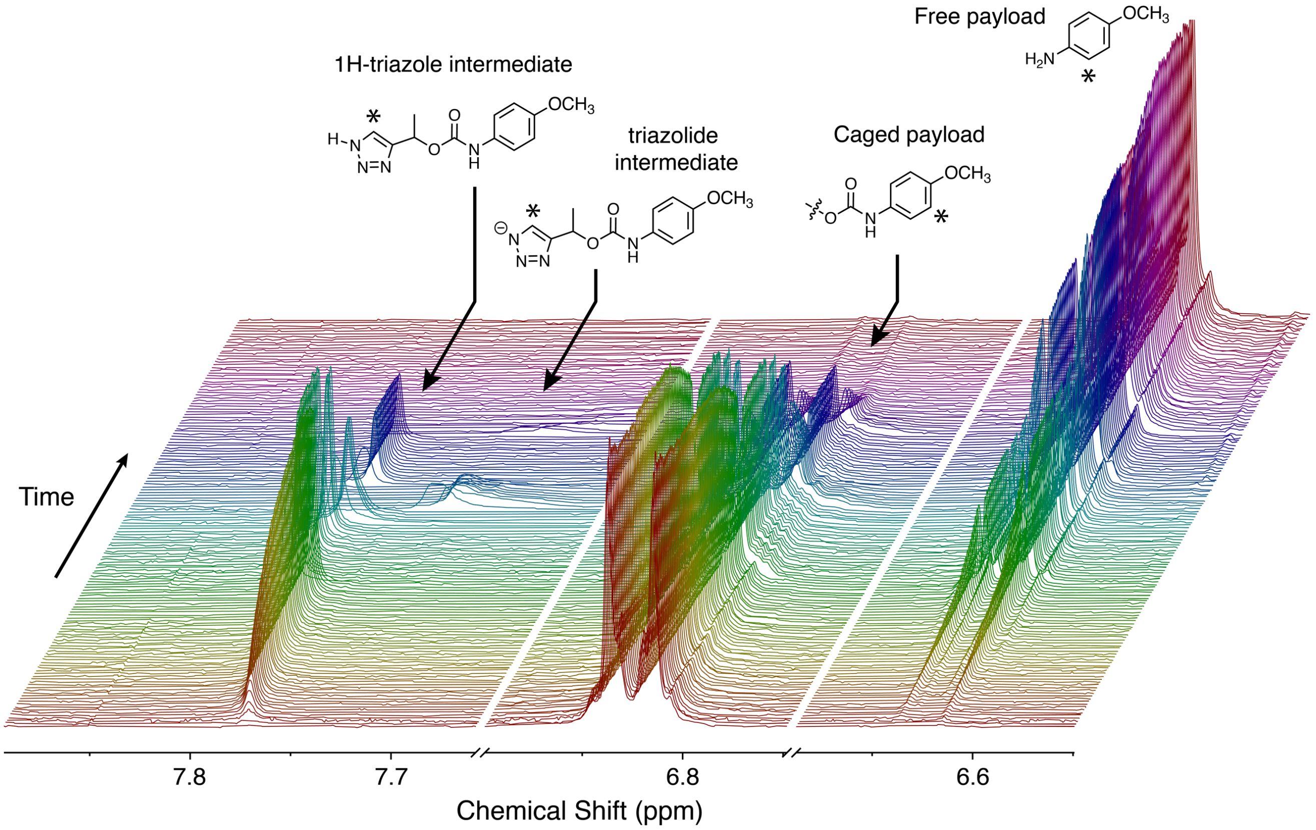

Figure S96. Stacked ^{1}H NMR spectra (400 MHz, DMSO-d_6, 333 K) showing changes over time in the peak intensities of key species present during acid/base-mediated pausing/reactivation of the self-immolation cascade of model **1b**. Asterisks on the structures denote the nuclei being tracked in the spectra for each species. The equilibrium drawn between the 1*H*-triazole and triazolide forms of intermediate **4b** illustrates that the observed peak position is a weighted average of the triazole aromatic ^{1}H resonance that reflects the position of equilibrium between these two species in the fast-exchange NMR regime. Thus, under acidic conditions intermediate **4b** is assumed to be fully protonated and thus 100% of the population is in the 1*H*-triazole form. Under basic conditions, the equilibrium lies somewhere between the 1*H*-triazole form and the fully deprotonated triazolide form (hence the up-field shift), with the position of equilibrium depending on the strength of the base present.

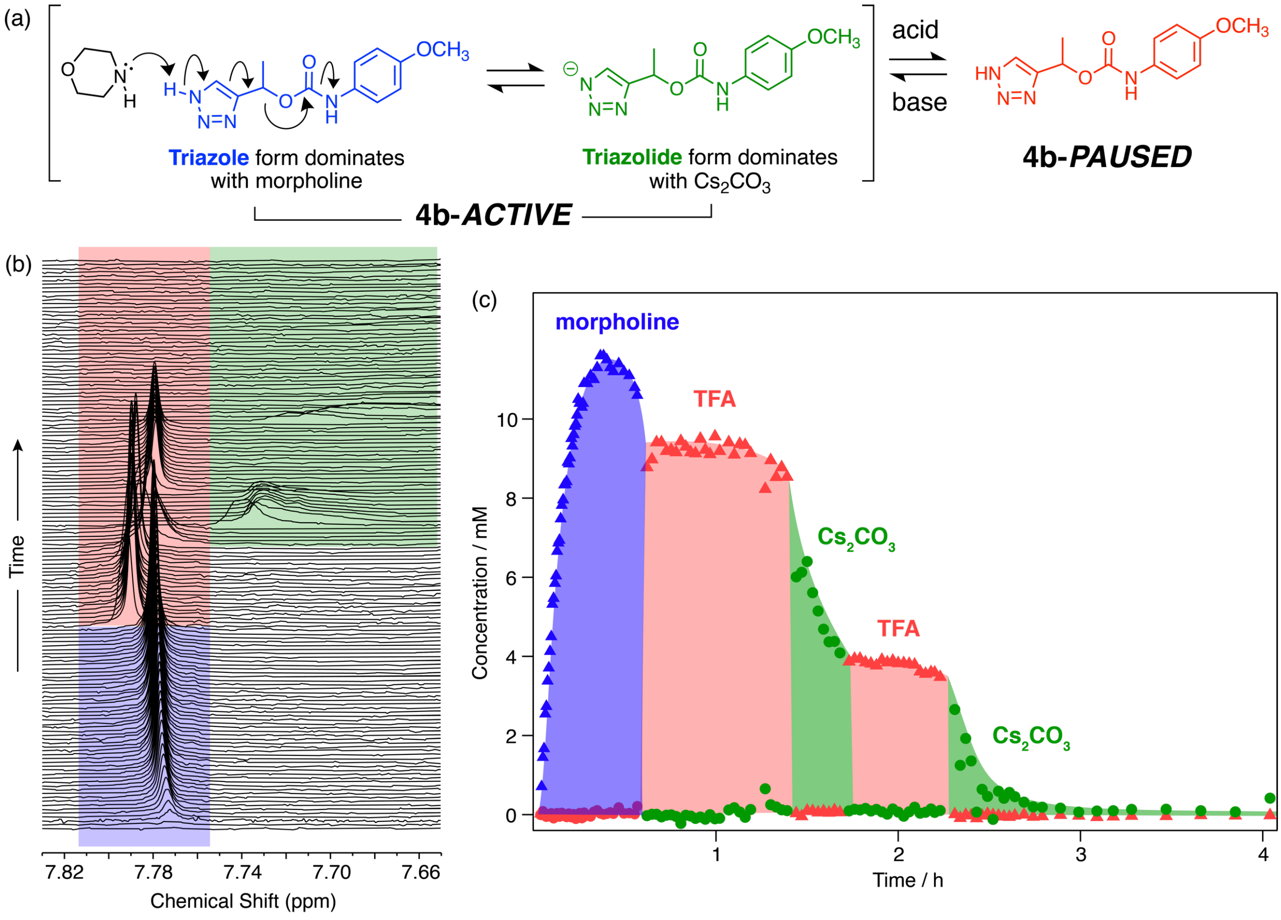

Figure S97. (a) Reaction scheme showing the relationship between **4b**-*ACTIVE*, which exists in equilibrium between its triazole and triazolide forms, and the fully protonated **4b**-*PAUSED*. We distinguish between the protonated 1*H*-triazole form of **4b**-*ACTIVE* and **4b**-*PAUSED* (which is essentially the same species) by the presence of base in the former case, which can give rise to a concerted deprotonation-elimination mechanism. The distinction arises from the fact that **4b**-*ACTIVE* readily undergoes 1,4-elimination, whereas **4b**-*PAUSED* does not. (b) Stacked ^{1}H NMR spectra (400 MHz, DMSO-d_6, 333 K) showing changes in the peak position of the aromatic triazole proton signal of intermediate **4b** (derived from the self-immolation of model **1b**) in the presence of morpholine (blue), TFA (red) and Cs_2CO_3 (green). Under acidic conditions (red), fully protonated **4b**-*PAUSED* is the dominant species. However, under basic conditions (blue and green) **4b**-*ACTIVE* is the dominant species. Depending on the strength of the base, **4b**-*ACTIVE* exists in a fast-exchange equilibrium between protonated and deprotonated forms, which is reflected by the position of the aromatic triazole proton resonance. In the presence of morpholine, the position of equilibrium lies closer to the 1*H*-triazole form (inferred from the similarity in chemical shift to the fully-protonated **4b**-*PAUSED* triazole resonance), whereas Cs_2CO_3 shifts the equilibrium toward the triazolide form (inferred from the broadening and up-field shift in the triazole proton resonance). (c) Plot of the concentrations of **4b**-*ACTIVE* and **4b**-*PAUSED*, color-coded to match the NMR spectra shown in (b), which is also colored according to the conditions present.

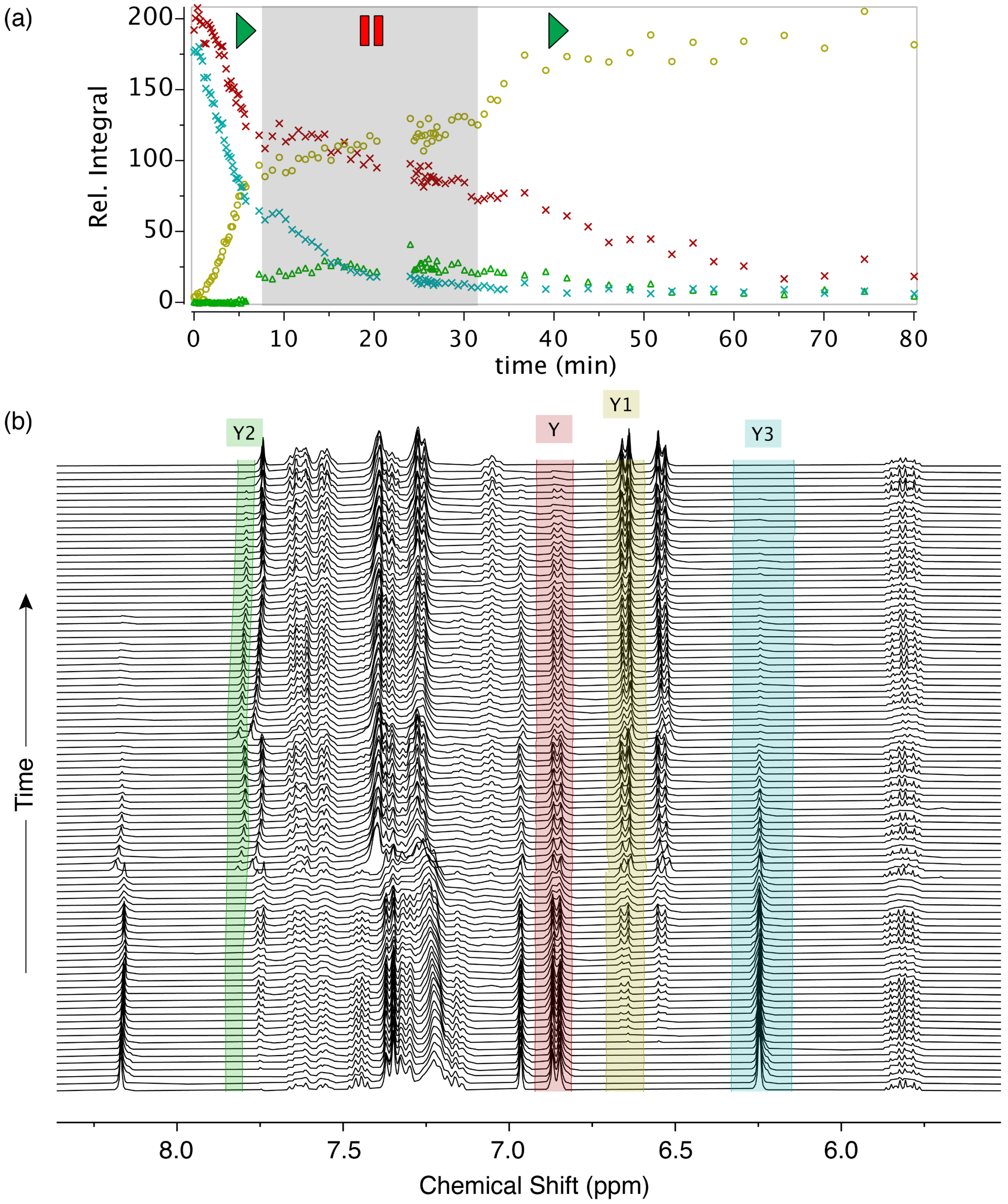

Figure S98. ^{1}H NMR acid/base-mediated switching experiment using model **1e** (400 MHz, DMSO-d_6, 333 K). **(a)** Relative integral plotted against time showing the payload phenylene resonances of the starting model (red), free anisidine (yellow), the 1*H*-triazole intermediate (green), and the secondary amine intermediate formed after trigger removal (blue). The active and dormant/slowed phases are denoted with pause and play icons, with the 'paused' phase shaded in grey. The data illustrate that this compound does not enter a stable paused phase. **(b)** Stacked NMR spectra showing integrated regions, color-coded to match the trace in (a). Integrals have been normalized to the hexamethyldisilane internal standard (t_0 shown at bottom).

S9. Acid/base-mediated switching in DMSO-d_6/D_2O 8:2 (Models 1a and 1b)

Typical dynamic switching experiment

A standard 5 mm NMR tube was charged with a DMSO solution of model **1b** (145 µL, 84.4 mM; ~12 µmol, 1.0 equiv.), morpholine (53 µL, 0.61 mmol, 50 equiv.) and D_2O containing *t*BuOH as an internal concentration standard (87 µL, 15.6 mM)) in DMSO-d_6 (104 µL). The tube was inserted into the NMR spectrometer, equilibrated at 60 °C until stable (~5 min) then the spectrometer was tuned and matched, locked and shimmed and an initial spectrum recorded. The tube was ejected from the spectrometer, a suspension of $Pd(PPh_3)_4$ (1 mg, 0.9 µmol) in DMSO-d_6 (100 µL) added and the tube mixed rapidly before returning the sample to the spectrometer (sample was outside spectrometer for <1 min). Upon returning to the spectrometer, the sample was re-shimmed and kinetics timepoints were collected immediately. The frequency at which spectra were collected was controlled by varying the number of dummy scans.

To pause the self-immolation cascade, the NMR tube was ejected from the spectrometer, an aliquot of aqueous hydrochloric acid (10.2 M, 24 µL, 0.24 mmol, 20 equiv.) was added and the tube inverted rapidly to mix then returned to the spectrometer within 1 min. The sample was re-shimmed and kinetics timepoints collected immediately. After ~5 min, the sample was ejected again and an aliquot of NaOH in D_2O (19.5 M, 13 µL, 0.26 mmol, 21 equiv.) was added to restart the cascade. The tube was inverted rapidly to mix then returned to the spectrometer within 1 min. Kinetics timepoints were collected immediately without re-shimming the sample. After an additional 5 min, a second aliquot of aqueous HCl was added (10.2 M, 30 µL, 0.31 mmol, 25 equiv.) and the tube inverted rapidly to mix then returned to the spectrometer within 1 min. The sample was re-shimmed and kinetics timepoints collected immediately. A final aliquot of NaOH in D_2O (19.5 M, 19 µL, 0.37 mmol, 30 equiv.) was added to restart the cascade a final time. The cascade was observed to restart and was allowed to proceed until the end of the NMR session, at which time >90% payload release has been achieved. Data for the acid/base-mediated switching of models **1a** and **1b** DMSO-d_6/D_2O (8:2 *v*/*v*) are shown Figures S99 and S100. Pausing experiments were not performed for any models in the fast kinetics regime due to their extremely fast cascades, which precluded aliquot additions and re-shimming on a timescale amenable to dynamic switching.

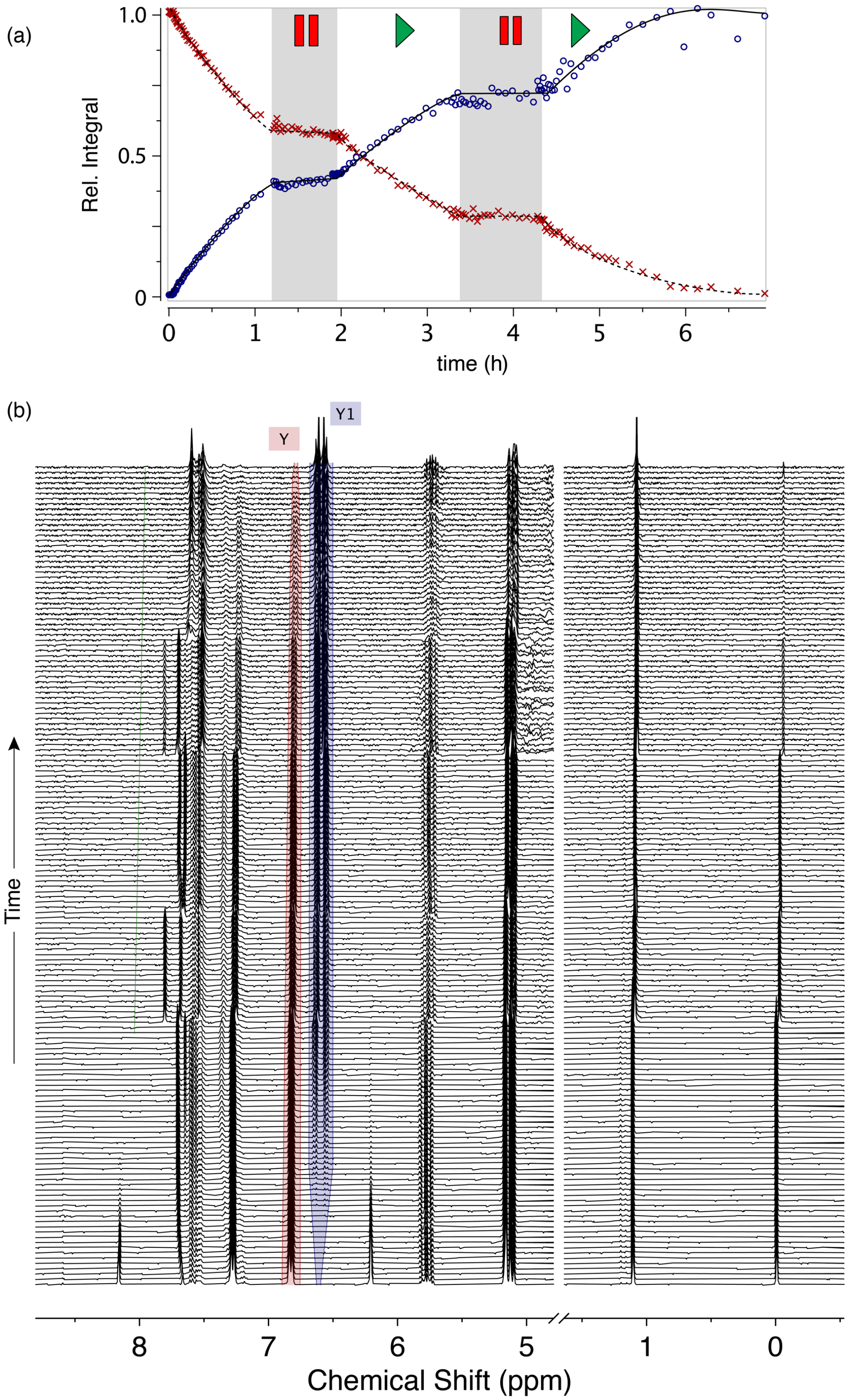

Figure S99. ^{1}H NMR acid/base-mediated switching experiment using model **1a** (400 MHz, DMSO-d_6/D_2O, 333 K). **(a)** Relative integral plotted against time showing the payload phenylene resonances of the starting model (red) and free anisidine (blue). Active and paused phases are denoted with pause and play icons, with the 'paused' phases shaded in grey. **(b)** Stacked NMR spectra showing integrated regions, color-coded to match the trace in (a). Integrals have been normalized to the *t*BuOH internal standard (t_0 shown at bottom).

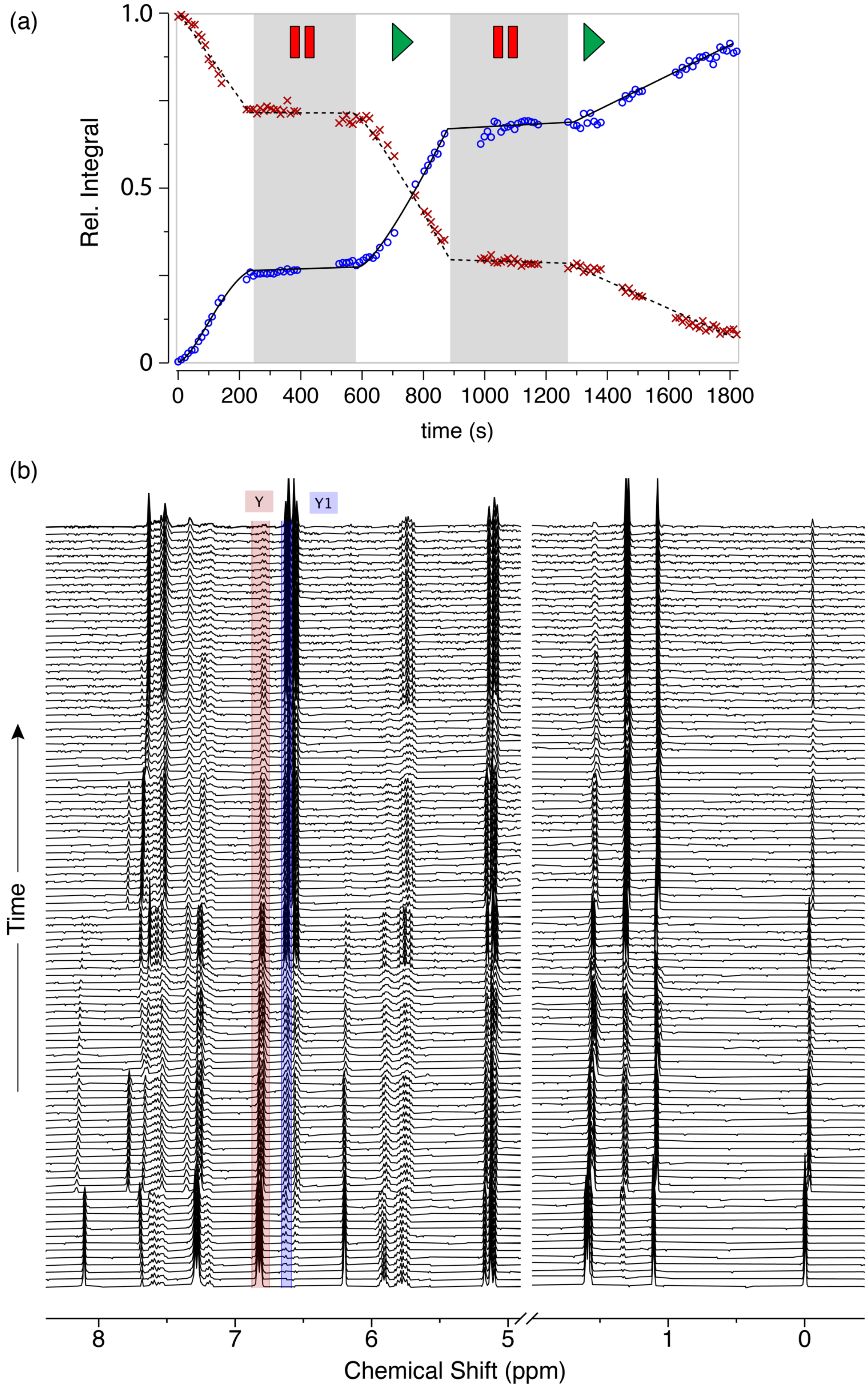

Figure S100. ^{1}H NMR acid/base-mediated switching experiment using model **1b** (400 MHz, DMSO-d_6/D_2O, 333 K). **(a)** Relative integral plotted against time showing the payload phenylene resonances of the starting model (red) and free anisidine (blue). Active and paused phases are denoted with pause and play icons, with the 'paused' phases shaded in grey. **(b)** Stacked NMR spectra showing integrated regions, color-coded to match the trace in (a). Integrals have been normalized to the *t*BuOH internal standard (t_0 shown at bottom).

S10. References

1. Pittelkow, M.; Lewinsky, R.; Christensen, J. B., Selective Synthesis of Carbamate Protected Polyamines Using Alkyl Phenyl Carbonates. *Synthesis* **2002,** *2002*, 2195-2202.
2. Rao, H.; Damian, M. S.; Alshiekh, A.; Elmroth, S. K. C.; Diederichsen, U., Design, synthesis and DNA interactions of a chimera between a platinum complex and an IHF mimicking peptide. *Org. Biomol. Chem.* **2015,** *13*, 11704-11713.
3. Chassaing, S.; Kueny-Stotz, M.; Isorez, G.; Brouillard, R., Rapid Preparation of 3-Deoxyanthocyanidins and Novel Dicationic Derivatives: New Insight into an Old Procedure. *Eur. J. Org. Chem.* **2007,** *2007*, 2438-2448.
4. Hoekstra, W., J.; Yates, C., M Metalloenzyme inhibitor compounds. WO2013/90210, 20 June, 2013.
5. Petrone, D. A.; Isomura, M.; Franzoni, I.; Rössler, S. L.; Carreira, E. M., Allenylic Carbonates in Enantioselective Iridium-Catalyzed Alkylations. *J. Am. Chem. Soc.* **2018,** *140*, 4697-4704.
6. Ren, L.; Jiao, N., PdCl2 catalyzed efficient assembly of organic azides, CO, and alcohols under mild conditions: a direct approach to synthesize carbamates. *Chem. Commun.* **2014,** *50*, 3706-3709.
